# Quantification of the actual composition of polymeric nanocapsules: a quality control analysis

**DOI:** 10.1007/s13346-022-01150-5

**Published:** 2022-03-18

**Authors:** Germán Berrecoso, José Crecente-Campo, María José Alonso

**Affiliations:** 1 Center for Research in Molecular Medicine and Chronic Diseases (CiMUS), Universidade de Santiago de Compostela, 15782 Santiago de Compostela, Spain; 2 Department of Pharmacology, Pharmacy, and Pharmaceutical Technology, School of Pharmacy, Universidade de Santiago de Compostela, 15782 Santiago de Compostela, Spain; 3 IDIS Research Institute, Universidade de Santiago de Compostela, 15782 Santiago de Compostela, Spain

**Keywords:** Polymeric nanoparticles, Nanocapsules, Mass spectrometry

## Abstract

Nanocapsules (NCs) are drug delivery nanosystems that contain an oily core, stabilized by a surfactant, and surrounded by a polymeric shell. The assembling of the components is based on physical and physicochemical forces, and, hence, usually, only a fraction of each component is finally part of the NCs’ structure, while the remaining amount might be solubilized or forming micelles in the NCs’ suspending medium. Usually, reports on the characterization of nanostructures simply indicate the association efficiency of the loaded drugs instead of their complete final composition. In this work, we have developed a liquid chromatography (LC) mass spectrometry (MS) methodology that allows the quantification of all the components of a series of NCs prepared by different techniques, namely dl-α-tocopherol; d-α-tocopherol polyethylene glycol 1000 succinate; benzethonium; lecithin; hexadecyltrimethylammonium; 1,2-dioleoyl-3-trimethylammoniumpropane; caprylic/capric triglycerides; macrogol 15-hydroxystearate; polysorbate 80; polysialic acid; hyaluronic acid; and polyethylene glycol polyglutamic acid. The LC–MS method was validated in terms of linearity (0.9383 < *r*^2^ < 0.9997), quantification limits, and recoveries of the isolated NCs’ and waste fractions. The final composition of the isolated NCs was found to strongly depend on their composition and preparation technique. In our view, the rigorous quantification of the exact composition of nanosystems is essential for the progress of nanotechnology. This quantitative analysis will allow researchers to draw more accurate conclusions about the influence of the nanosystems’ composition on their biological performance.

**Graphical abstract:**

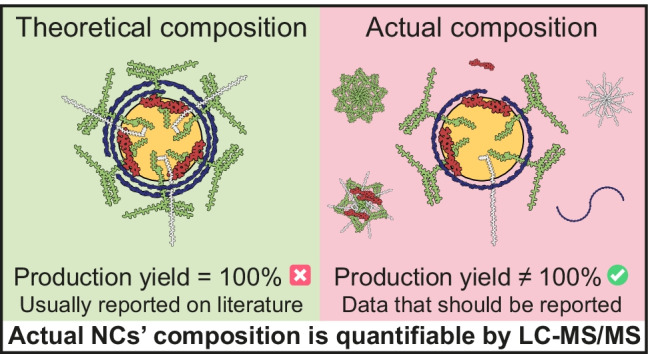

**Supplementary Information:**

The online version contains supplementary material available at 10.1007/s13346-022-01150-5.

## Introduction

NCs are nanosystems composed of an oily core, a stabilizing layer of one or more surfactants, and a polymeric shell. They can be produced by easy, fast, and low-energy input techniques, such as solvent displacement (SD) [1] and self-emulsification (SE) [2]. These techniques enable the assembling of the components in the form of a nanocapsular structure, where the incorporation of the different components is driven by several mechanisms, such as the solubility of molecules in the formulation media, the ionic and/or hydrophobic interactions among different molecules, and the self-assembling of amphiphilic substances into micellar structures. In addition, some isolation techniques that are usually employed to remove organic solvents and the non-associated fraction of drugs can promote aggregation or caking, as well as the elimination of some of the particles, reducing, even more, the actual production yield in the final formulation [3]. For example, when using ultracentrifugation for the purification of nanostructures, sedimentation or flotation speed under centrifugal forces is dictated by the Stokes’ law, this being proportional to the difference between the density of the medium and the density of the particles and to the square of the particle diameter [4]. This means that, in a polydisperse population, particles with a smaller size or with a density closer to that of the media might remain in suspension and not be properly separated. Consequently, the production yields are usually reported to be significantly lower than 100% [5–7].

Most of the published works in the nano-drug delivery field simply evaluate the association efficiency (AE) of the components with biological activity or targeting capabilities; however, very rarely do authors report on the NCs’ final composition. In this regard, some authors choose to chemically modify some NCs’ components with fluorophores in order to determine their AE; however, this method erroneously relies on the premise that the incorporation of a fluorophore does not alter the incorporation of the labeled component. Finally, other assays based on absorbance are described for both, the quantification of some unaltered compounds, such as dl-α-tocopherol (285 nm) [8], and, derivatized products, such as polysaccharides reacting with carbazole/H_2_SO_4_ (530 nm) [9]. Nevertheless, the complex formulation matrixes usually interfere with these methodologies, and thus, it is commonly necessary to perform chromatography-based purifications. Taking all into account, a chromatography separation method followed by a robust and highly specific quantification technique, such as MS/MS, was thought to be an ideal methodology to face the NCs’ composition characterization challenge.

Although several LC–MS/MS methods have been published for the individual quantification of some of the components used in the evaluated NCs, an all-in-one methodology for the quantification of the total NC composition has not been published yet. Based on this premise, the objective of this work has been the precise quantification of all the oils, surfactants, and polymers composing the actual structure of five SD and SE NCs. Overall, we demonstrate that, despite the chemical heterogenicity among the set of the NCs’ components, it is plausible to quantify their exact composition by LC–MS/MS before and after NCs’ isolation.

## Materials and methods

### Materials

Materials used are as follows: dl-α-tocopherol (MilliporeSigma, Burlington, MA, USA); d-α-tocopherol polyethylene glycol 1000 succinate (TPGS) (Antares Health Products, Jonesborough, TN, USA); benzethonium chloride (BZT) (Spectrum Chemical Mfg. Corp., New Brunswick, NJ, USA); lecithin (Epikuron™ 145 V; batch phosphatidylcholine content: 51.7% w/w) (Cargill, Wayzata, MN, USA); hexadecyltrimethylammonium bromide (CTAB) (Sigma-Aldrich, St. Louis, MO, USA); 1,2-dioleoyl-3-trimethylammonium-propane chloride (DOTAP) (Avanti Polar Lipids, Alabaster, AL, USA); caprylic/capric triglycerides (Miglyol® 812 N) (IOI Oleo GmbH, Hamburg, Germany); Kolliphor® HS15 (BASF SE, Ludwigshafen, Germany); polysorbate 80 (Tween® 80) (Merck KGaA, Darmstadt, Germany); polysialic acid 26 kDa (PSA) (Serum Institute of India, Pune, India); hyaluronic acid 41–65 kDa (HA) (Lifecore Biomedical, Chaska, MN, USA); ethanol absolute (Scharlau, Barcelona, Spain); polyethylene glycol (5 kDa) polyglutamic acid (10 u) (PEG·PGA) (Polypeptide Therapeutic Solutions, Paterna, Spain); acetone and phosphate-buffered saline (PBS) tablets (Thermo Fisher Scientific, Waltham, MA, USA); sulfuric acid 96% and hydrochloric acid 37% (Panreac, Quimica, Castellar del Vallès, Spain).

### Nanocapsule preparation

For SD-*l-*PEG-HA, 500 μL of oil phase (27 mg·mL^−1^ dl-α-tocopherol, 8 mg·mL^−1^ TPGS, and 1 mg·mL^−1^ BZT in ethanol) was poured directly to 1.5 mL of aqueous phase (HA 1.33 mg·mL^−1^) under magnetic stirring at 700 rpm. Stirring was kept until the final volume was lower than 1.5 mL. Then, the volume was completed to 2 mL using ultrapure water. The sample was ultracentrifuged at ~ 110,000* g* (40,000 rpm) for 1 h at 15 °C (OptimaTM L-90 K Ultracentrifuge equipped with a 70.1 Ti rotor, and 10 mL open-top polycarbonate tubes (16 × 76 mm, Ref. 355,630) Beckman Coulter, Brea, CA, USA). The infranatant was taken using a syringe and kept separately. The cream-like supernatant was resuspended to 750 μL with ultrapure water.

For SD-noPEG-HA and SD-noPEG-PSA, 5 mL of oil phase (2.96 mg·mL^−1^ of caprylic/capric triglycerides, 0.75 mg·mL^−1^ of lecithin, and 0.15 mg·mL^−1^ of CTAB in a mixture of acetone and ethanol 19:1) was added to 10 mL of 0.25 mg·mL^−1^ aqueous solution of the polymer (HA or PSA) dropwise using a Pasteur pipette under magnetic stirring at 700 rpm. Acetone was removed using a rotavapor until the sample volume was 5 mL. Then, it was ultracentrifuged at ~ 43,000* g* (25,000 rpm) for 30 min at 15 °C. The infranatant was withdrawn using a syringe and kept separately. The cream-like supernatant was resuspended to 750 μL with ultrapure water.

For SE-*b*-PEG-HA, the oil phase was composed of a mixture of 295 mg of caprylic/capric triglyceride, 290 mg of polysorbate 80, and 1 mg of BZT. 4.375 mL of aqueous phase (0.286 mg·mL^−1^ of HA and 2.86 mg·mL^−1^ of Kolliphor® HS15 in PBS 1 ×) was quickly poured over the oil phase at 1100 rpm using a micropipette. The sample was isolated using a tangential flow filtration (TFF) machine (KrosFlo® KR2i, Repligen, Waltham, MA, USA) equipped with a 500-kDa cutoff-modified polyethersulfone column. The resulting ~ 5 mL nanoparticle solution was diafiltered 3 times with PBS as exchanging buffer at a speed of 9 mL·min^−1^.

For SD-noPEG-PEG·PGA, 300 μL of oil phase (45 mg·mL^−1^ dl-α-tocopherol and 1.66 mg·mL^−1^ DOTAP in ethanol) was poured directly to 1.5 mL of PEG·PGA 1.33 mg·mL^−1^ in water under magnetic stirring at 700 rpm. Stirring was kept until the final volume was lower than 1.0 mL and the volume was completed to 2 mL using ultrapure water. Then, it was ultracentrifuged at ~ 110,000* g* (40,000 rpm) for 1 h at 15 °C. The infranatant was withdrawn using a syringe and kept separately. The cream-like supernatant was resuspended to 750 μL with ultrapure water.

### Equipment

The LC–MS/MS system consisted of a Waters Acquity H-Class UPLC (Waters Corporation, Milford, MA, USA) equipped with a 50 μL extension loop, and a Waters Xevo Triple Quadrupole Detector. A Kinetex® 1.7 μm C18 100 Å 50 × 2.1-mm column (Phenomenex, Torrance, CA, USA) was used for the analysis of all components but digested PEG·PGA, where a Kinetex® 1.7 μm HILIC 100 Å 50 × 2.1-mm column was used instead.

### Sample preparation

The NCs’ composition analysis was carried out in two steps, with different sample treatments.

First, all the compounds but the polymers were quantified after dilution and dissolution of each sample with acetonitrile. For SD-*l-*PEG-HA, the non-isolated formulation, as well as the infranatant solutions after ultracentrifugation, were diluted 3750 × , while the cream-like supernatant was diluted 10,000 × . For SD-noPEG-HA and SD-noPEG-PSA, the formulations, before rotary evaporation, were diluted 500 × ; the infranatant solutions after ultracentrifugation were diluted 667 × ; and the cream-like supernatant was diluted 5000 × . For SE-*b-*PEG-HA, the formulations before filtration and the retentate were diluted 25,000 × , and the permeate was diluted 8333 × . For SD-noPEG-PEG·PGA, the non-isolated formulation, as well as the infranatant solutions after ultracentrifugation, were diluted 4000 × , while the cream-like supernatant was diluted 10,000 × .

Polymers were digested in acidic conditions. For SD-*l-*PEG-HA, 60 μL of formulation before centrifugation, infranatant, and supernatant was mixed with 40 μL of ethanol and heated in a water bath at 68 °C in a 2-mL microtube. For SD-noPEG-HA and SD-noPEG-PSA, 69.5 μL of formulation before centrifugation were mixed with 13.67 μL of water and 16.83 μL of ethanol. In the case of SE-*b-*PEG-HA, 60 μL of formulation before filtration and retentate were mixed with 40 μL of ethanol. One hundred eighty microliters of permeate was mixed with 20 μL of ethanol. In all cases but the SE-*b-*PEG-HA permeate, 125 μL of 4 M sulfuric acid pre-heated at 68 °C was added to each tube. Digestions were carried out for exactly 60 s, then neutralized with 1 mL of 1 M aqueous solution of NaOH; shaken for 10 min at 2000 rpm; and centrifuged at 20,817* g*, 10 min, 20 °C. In the case of the SE-*b-*PEG-HA permeate, where more sample volume was needed due to the low HA concentration, 250 μL of 4 M aqueous sulfuric acid and 400 μL of sodium hydroxide 5 M were used instead. Consequently, monomers, dimers, and oligomers of these carbohydrates were generated.

Finally, for SD-noPEG-PEG·PGA, 85 μL of formulation before ultracentrifugation, infranatant, and 1.63 × diluted cream-like supernatant in ultrapure water were mixed with 15 μL of ethanol and 100 μL of HCl 37% in a screw-capped 1.5-mL microtube. Tubes were shaken and put in an oil bath at 110 °C for 24 h. Then, 800 μL of NaOH 1.5 M were added to each vial. PEG·PGA samples were shaken and centrifuged as abovementioned for other polymers.

All samples were filtered through Millex®-GV 0.22 μm PVDF filter units (Merk, Kenilworth, NJ, USA) after their first dilution with acetonitrile or after the centrifugation step performed following the polymer digestion.

### Standard preparation

Calibration curves for all substances were made together, respecting the ratios between them in the original sample. Eight concentration levels were tested in all cases but polymers, where 6 levels were used instead. All calibrating samples were also filtered through 0.22 μm PVDF filters after their first dilution with acetonitrile or after neutralization of their acidic digestion. All vials were analyzed three times in each replicate. Calibration curves for non-polymeric compounds were prepared including the NCs’ coating polymer at their maximum theoretical concentration to mimic the sample and correct the possible matrix effect the other substances might suffer from.

Calibration curves of HA, PSA, and PEG·PGA were done digesting a final volume of 100 μL (except for the permeate of SE-*b-*PEG-HA, which was 200 μL) containing all the other compounds present in the samples to quantify. In the case of the analysis of the bound and free fractions, artificial matrixes were built considering the results obtained in the quantification of the non-polymeric compounds. Solvent ratios were also fixed in the standard vials to match the sample treatments, being 60% water and 40% ethanol in all the cases of carbohydrate quantification but SD-noPEG formulations before isolation, which was 60% water, 22% acetone, and 18% ethanol; and the permeate of SE-*b*-PEG-HA, which was 90% water and 10% ethanol. The ethanol percentage of the calibrating solutions for PEG·PGA analysis was fixed to 15% (v/v). The digestion and following steps were carried out as abovementioned. Calibration ranges are detailed in Supplementary Table 1.

### Processing method

Chromatographic peaks were smoothed by the Savitzky-Golay method before their integration, and the calibration curves for linear fit were obtained with 1/ × weighting of the obtained areas.

### Chromatographic method

All the compounds but the polymers were analyzed with the following solvents: solvent A: 50 mM formic acid (HCOOH) (VWR International, Radnor, PA, USA) and 2 mM ammonium formate (NH_4_HCOO) (Sigma-Aldrich, St. Louis, MO, USA) aqueous solution, solvent B: 95% acetonitrile (Scharlau, Barcelona, Spain) and 5% water containing 50 mM HCOOH and 2 mM NH_4_HCOO (v/v), solvent C: water, solvent D: isopropyl alcohol (VWR International, Radnor, PA, USA). The injection volume was 5 μL; the column temperature was 50 °C; the sample temperature was 20 °C; and the flow rate was 0.400 mL·min^−1^. The chromatographic method varied with the formulation, and it is summarized in Supplementary Table 2.

The carbohydrate fragments were analyzed based on the work of Vigliano et al. [10], in which solvent A was 50 mM HCOOH and 2 mM NH_4_HCOO aqueous solution, and solvent B was methanol (Thermo Fisher Scientific, Waltham, MA, USA). The injection volume was 50 μL. The column and sample temperatures were not modified, and the flow rate was 0.200 mL·min^−1^. The chromatographic method was common for HA and PSA (Supplementary Table 3). The flow was diverted to waste during the first 3.9 min to avoid the non-volatile salt Na_2_SO_4_ from entering the detector.

In the case of PEG·PGA, solvent A was 75 mM HCOOH and 5 mM NH_4_HCOO aqueous solution and solvent B was 90% acetonitrile and 10% water (v/v) containing 75 mM HCOOH and 5 mM NH_4_HCOO (Supplementary Table 4). The injection volume was 5 μL. The column was set at 35 °C and the flow rate at 0.300 mL·min^−1^. The flow was diverted to waste during the first 9 min.

Examples of these chromatograms are shown in Supplementary Table 5.

### Mass spectrometry method

In all the cases but the polymers, the MS/MS detector was tuned as follows: ionization mode to positive electrospray ionization [ESI ( +)], source capillary voltage: 3.50 kV, desolvation temperature: 600 °C, source temperature: 150 °C, desolvation gas flow: 900 L·h^−1^, cone gas flow: 10 L·h^−1^. In the case of HA and PSA, the following parameters were used instead: source capillary voltage: 3.20 kV, desolvation temperature: 450 °C, desolvation gas flow: 350 L·h^−1^. MS parameters for PEG·PGA analysis were source capillary voltage: 1.00 kV, desolvation temperature: 550 °C, desolvation gas flow: 1000 L·h^−1^, cone gas flow: 50 L·h^−1^. The MS and MS/MS spectra for each detected molecule are detailed in Supplementary Table 6, while the conditions used for the quantification of each molecule, including the parent’s and daughter’s structures, declustering potentials, and collision energies are summarized in Supplementary Table 7.

### Method validation

Method validation was performed following ICH guidelines [11]. Specificity was confirmed subjecting each compound to the LC–MS/MS method individually and checking there was no interference in the chromatograms of the rest of the molecules. Linearity was assessed through the analysis of the coefficients of determination (*r*^2^) and evaluation of the residual plots. The quantification limit was calculated based on the standard deviation of the blanks, as $LoQ=10\cdot {\sigma }_{x}\cdot {S}^{-1}$, where ${\sigma }_{x}$ is the standard deviation of the response of 10 blank injections and $S$ the slope of the calibration curve. Precision and accuracy were proved for three calibration levels (low, medium, and high), by three injections of three different vials, through the pooled relative standard deviations of the signals (pooled RStD%) and the relative differences of the estimated and actual concentrations (RE%) of the standards, respectively. Complementary, the amount of each substance in the formulation before isolation was checked and confirmed to match the theoretical composition. The same determination was carried out for the two fractions generated after isolation as well.

## Results and discussion

### Description of the different NCs

**Fig. 1:**
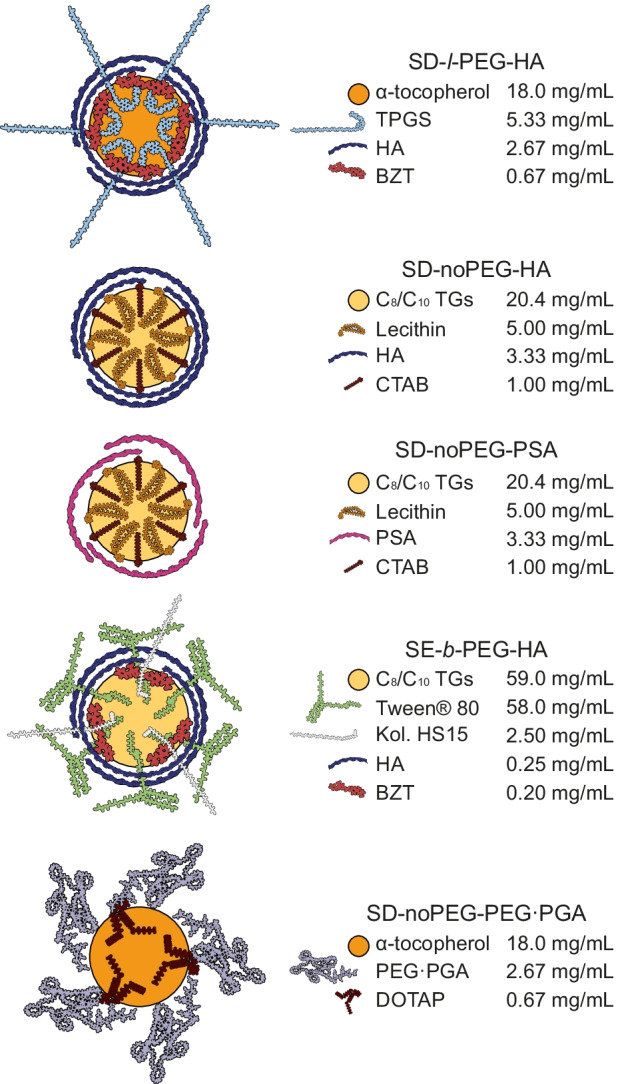
Schematic representation of the different prototypes of NCs and composition of the non-isolated samples. SD, solvent displacement; SE, self-emulsifying; *l*-PEG, polyethylene glycol (PEG) present in a linear disposition in the main surfactant; *b*-PEG, branched-disposed PEG on the main surfactant; noPEG, no PEGylated components. Polymers: HA, hyaluronic acid; PSA, polysialic acid; PEG·PGA, polyethylene glycol polyglutamic acid. PEGylated surfactants: TPGS, d-α-tocopherol polyethylene glycol 1000 succinate; Kol. HS15, Kolliphor® HS15, Tween® 80. Non-PEGylated surfactants: BZT, benzethonium; CTAB, hexadecyltrimethylammonium; DOTAP, 1,2-dioleoyl-3-trimethylammoniumpropane, lecithin. Oils: C_8_/C_10_ TGs, caprylic and capric acid triglycerides, α-tocopherol. The disposition of the components on the NC was assumed based on their physicochemical properties, mainly hydrophobicity and charge

Five different NCs with a particle size of approximately 150 nm were formulated by two commonly used formulation methods (SD and SE). A schematic representation of the tested NCs, as well as their theoretical composition, is presented in Fig. 1.

In total, two different oils (dl-α-tocopherol and Miglyol® 812 N), two polysaccharides (HA and PSA), one modified oligopeptide (PEG·PGA), and seven surfactants were tested. Surfactants were classified attending to the absence/presence of polyethylene glycol (PEG) moieties.

More specifically, four of the surfactants were not PEGylated (noPEG): benzethonium chloride, DOTAP, CTAB, and Epikuron™ 145 V. PEGylated surfactants were classified attending to the disposition of the PEG chains into two categories: di-block surfactants, containing a unique and lineal PEG chain (*l*-PEG), this was the case of d-α-tocopherol polyethylene glycol 1000 succinate and macrogol 15-hydroxystearate; and surfactants with branched PEG (*b*-PEG), containing several PEG chains, like polysorbate 80. NCs were denominated in a tri-block naming, indicating, firstly, their formulation technique (SD or SE); secondly, the nature of the predominant PEGylated surfactant (*l*-PEG, *b*-PEG, or noPEG); and lastly, their polymeric coating (HA, PSA, or PEG·PGA).

### Development of the LC–MS method

The development of the MRM (multiple reaction monitoring) methods of dl-α-tocopherol [12, 13], BZT [14, 15], DOTAP [16], and CTAB [17] was straightforward as they are easily fragmentable molecules with a unique and defined chemical structure. The rest of the components were more challenging.

First, some reagents are a mixture of several molecules. For example, Epikuron™ 145 V is a type of lecithin whose major components are phosphatidylcholines (PCs) of several molecular weights (MW). Since the proportion of each specific PC might change from batch to batch, choosing the most abundant PCs is of crucial relevance for the characterization of the NCs’ composition. Usefully, all PCs can be fragmented into a common daughter, phosphocholine (*m/z* 184.1) [18, 19]. A parent scan of this ion revealed that the most predominant PCs in the Epikuron™ 145 V batch used here possessed an MW of 756.6, 758.6, 780.6, and 782.5 Da.

An equal contribution of these molecules was assumed. Of note, some PC molecules present on Epikuron™ 145 V might be constitutional isomers, that is, they have the same atoms but arranged in a different order, which might cause broadening of their chromatographic peaks. The study of the exact identities within these MW is out of the scope of this work. Another example was Miglyol® 812 N, which is composed of a mixture of four different triglycerides of caprylic and capric fatty acids (C_8_-C_8_-C_8_, C_8_-C_8_-C_10_, C_8_-C_10_-C_10_, and C_10_-C_10_-C_10_ triglycerides). Moreover, in this last case, no parent molecules could be detected without the use of NH_4_HCOO-enriched mobile phases. NH_4_HCOO allows the detection of the parent molecules through the formation of an ammonium adduct, shifting up the detected *m/z* in 18 units [20]. Consequently, the NH_4_^+^ adducts of these four molecules were quantified. Since their concentration was similar in all the tested samples, an equal contribution was also assumed.

Some materials used during NCs’ preparation are complex mixtures of a series of slightly different molecules. This is the case of polysorbate 80, whose MS spectrum contained a vast number of different *m/z* signals, being *m/z* 804.6 the most intense. Since polysorbate 80 presented a predominant daughter of *m/z* 309.3 [21], we have quantified it through the MRM transition *m/z* 804.6 → 309.3. Similarly, the commercial product Kolliphor® HS15 is a mixture of macrogol-15-hydroxystearate (~ 70%) and unconjugated polyethylene glycol of different molecular weights (~ 30%). In this work, we have only quantified macrogol-15-hydroxystearate, as the unconjugated polyethylene glycol is very unlikely to form part of the NCs due to its high hydrophilicity. The MS/MS analysis of unconjugated polyethylene glycol is described elsewhere [22].

On other occasions, the molecule of interest may be labile and, consequently, be fragmented in the ESI source. This was the case for macrogol 15-hydroxystearate, where an in-source fragment of *m/z* 309.3 was detected [23]. Since this fragment could not be further fragmented in the collision cell, it was necessary to perform a pseudoMRM transition (*m/z* 309.3 → 309.3).

The quantification of the polymers was particularly challenging since, due to their high MW, they were not detectable by ESI ( +). To solve this problem, the polymers were chemically broken down into smaller fragments, being the last ones quantified. Hydrolysis of carbohydrates is described in the literature for several mechanisms including enzymatic, ultrasonic, thermal, oxidant, acidic, and alkali conditions [24]. HA and PSA were digested with H_2_SO_4_ to predominantly form hyaluronic acid dimers and dehydrated sialic acids of *m/z* 759.2 [10, 24, 25] and 274.1 [26, 27], respectively. Since no stable fragmentation was achieved, they were followed by SIM (selected ion monitoring). Similarly, proteins are reported to be hydrolyzed in acidic conditions, being hydrochloric acid 6 M, at high temperature (110 °C) for a long time (24 h) the classic method [28]. Acidic digestion was reported to be successful for the quantification of polyglutamic acid [29], but to the extent of our knowledge, no work has been published on PEG·PGA ESI ( +) LC–MS/MS quantification. The free amino acids originating after PEG·PGA digestion were quantified based on previous works for underivatized glutamic acid [30, 31].

Lastly, some molecules can be in-source fragmented and then fragmented again in the collision cell into granddaughters of the molecule of interest. This was the case of TPGS, where the MRM transition between the daughter ion *m/z* 557.4 and the granddaughter ion *m/z* 99.0 was used.

In general, calibration curves were found to be linear, with an average *r*^2^ of 0.985 (all *r*^2^ were within 0.9383 and 0.9997) and percentual relative residuals (%RRES) for each calibration level of less than ± 20% (Supplementary Table 8). In the case of the HA, PSA, and PEG·PGA, where the result of the digestion might be affected by the concentration of the other components, calibrating standards for the non-isolated formulation, isolated NCs (supernatant/retentate), and waste (infranatant/permeate) was designed incorporating the corresponding amount of all the previously quantified non-polymeric components.

The analysis of all the tested NCs’ components resulted to be both precise (repeatable) and accurate, being all intraday pooled RStD% < 9% and RE% < 13%.

The recoveries of all the substances of the NCs tested just after being formulated were close to 100% (Supplementary Table 9) of the theoretical composition, also suggesting the validity of the method.

Quantification limits (LoQs) for each substance were notoriously lower than the concentrations used in the calibration curves. For example, in the case of hyaluronic acid, the concentration of the lowest calibrating standard was, as an average, 400-fold of the LoQ. Additionally, the LoQs of BZT and CTAB resulted to be extraordinarily low, in the range of 200–700 pg·L^−1^.

The retention times of each molecule, calibration concentration range, *r*^2^, pooled RStD%, RE%, and LoQs are shown in Supplementary Table 1.

### Comparison between the different NCs

**Fig. 2:**
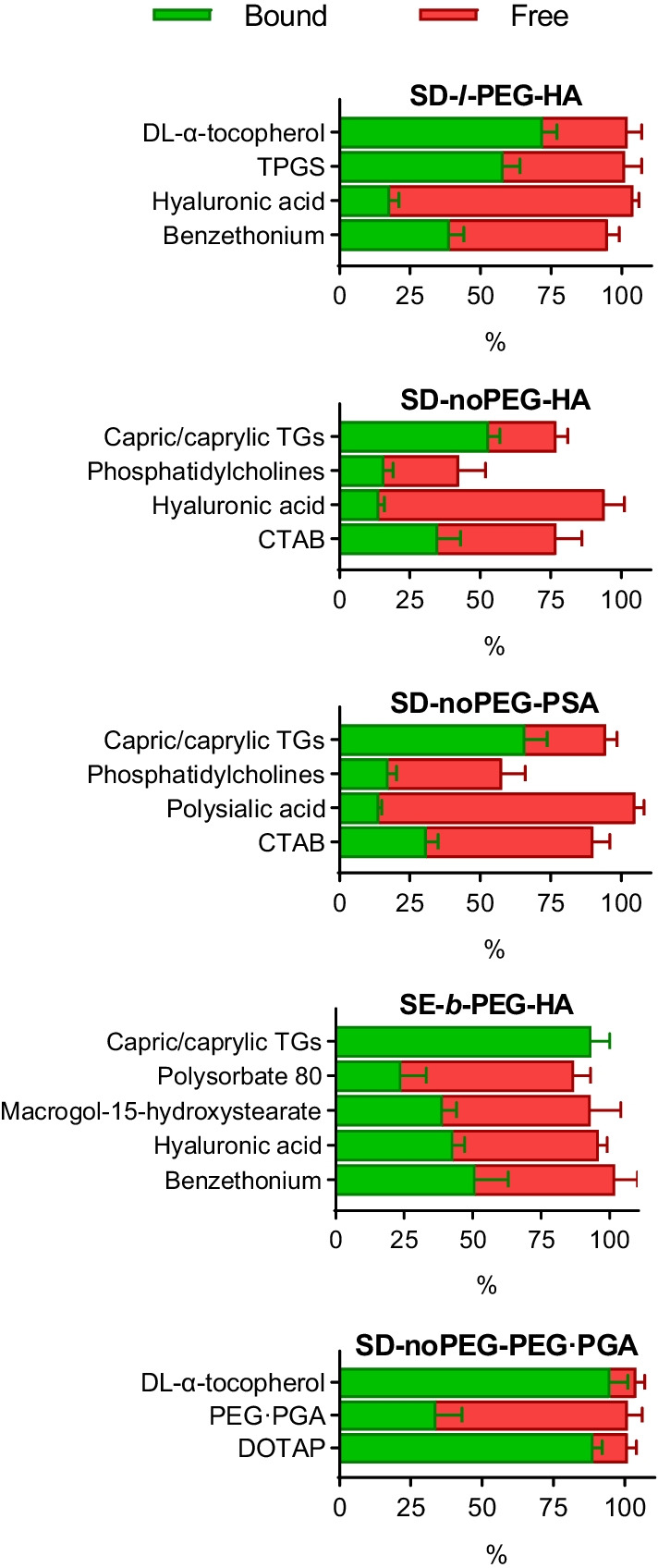
Detected associated (green) and non-associated (red) mass percentages out of the total added mass of each component of the tested NCs. TPGS, d-α-tocopherol polyethylene glycol 1000 succinate; TGs, triglycerides; CTAB, hexadecyltrimethylammonium; DOTAP, 1,2-dioleoyl-3-trimethylammonium-propane chloride; PEG·PGA, polyethylene glycol polyglutamic acid. Data were plotted as mean ± standard deviation (*n* = 3)

The NCs’ composition was initially evaluated before their isolation. At this stage, we evaluated the potential adsorption of biomaterials onto the vials, which resulted to be negligible. Another interesting checkpoint is the sum of the free and associated percentages after the purification step. In the case of SD-*l-*PEG-HA, SE-*b-*PEG-HA, and SD-noPEG-PEG·PGA, the mass of the free and associated fractions was, approximately, 100% of the initial, confirming the accuracy of the quantification method (Fig. 2). However, this was not the case for Epikuron™ 145 V in the SD-noPEG-HA and SD-noPEG-PSA, and Miglyol® 812 N in SD-noPEG-HA, due to the partial adsorption of these compounds on the rotavapor flask glass walls during the solvent-removal step. This loss was found to be constant between replicates (Supplementary Table 9).

The five tested NCs were compared in terms of surfactant, polymer, and PEG content. Sometimes, an excess of one or several components is necessary to promote the formation of the NCs or to avoid particle aggregation. As an example, SE NCs are reported to require a high surfactant concentration (30–60% w/w) to promote auto emulsification [32]. Consequently, components that are deliberately put in excess will present a low mass percentage in the isolated fraction. To compare the composition of different NCs, the amounts of surfactant and polymer per isolated particle, that is, the mass and molar ratio between these substances and the oils in the isolated formulation, were studied (Table 1).

**Table 1:** Mass and molar ratios of PEGylated surfactants, non-PEGylated surfactants, and polymer, referred to the oil quantity

Formulation	Mass ratio (mg/g of oil)			Molar ratio (mmol/mol of oil)		
	PEGylated surfactants	Non-PEGylated surfactants	Polymer	PEGylated surfactants	Non-PEGylated surfactants	Polymer
SD-*l-*PEG-HA	238	20	36	68.31	20.89	0.27
SD-noPEG-HA	N/A	73	45	N/A	86.64	0.40
SD-noPEG-PSA	N/A	58	34	N/A	84.94	0.40
SE-*b*-PEG-HA	265	2	2	105.62	2.26	0.02
SD-noPEG-PEG·PGA	N/A	35	53	N/A	22.45	3.64

*SD-l-PEG-HA*, lineal-polyethylene glycol-containing hyaluronic acid nanocapsules formulated by solvent displacement; *SD-noPEG-HA*, non-PEGylated hyaluronic acid nanocapsules formulated by solvent displacement; *SD-noPEG-PSA*, non-PEGylated polysialic acid nanocapsules formulated by solvent displacement; *SE-b-PEG-HA*, self-emulsifying branched-polyethylene glycol-containing hyaluronic acid nanocapsules; *SD-noPEG-PEG·PGA*, solvent displacement nanocapsules with non-PEGylated surfactants and a shell of polyethylene glycol polyglutamic acid; *N/A*, not applicable

The total amount of surfactant was found to be higher in the formulations containing PEGylated surfactants than in the ones incorporating non-PEGylated surfactants. This total amount of surfactant was particularly high for SE-*b-*PEG-HA NCs with 267 mg of surfactant per g of oil (108 mmol of surfactant per mol of oil). In the case of SD-*l-*PEG-HA, the surfactant association was intermediate, with 258 mg of surfactant per g of oil, equivalent to 89.2 mmol of surfactant per mol of oil. Lower surfactant association was observed for the rest of the systems, SD-noPEG-HA, SD-noPEG-PSA, and SD-noPEG-PEG·PGA NCs, this being 73, 58, and 35 mg of surfactant per g of oil (equivalent to 86.64, 84.94, and 22.45 mmol of surfactant per mol of oil), respectively.

The polymers forming the shell of the NCs may have different roles, such as the protection of their cargo from instability or degradation (i.e., PEG·PGA envelopment of mRNA nanocomplexes) [33], the improvement of their circulation time and their biodistribution [34], and their interaction with specific receptors (i.e., HA and CD44) [2]. Moreover, polymers can be further modified to include ligands for other receptors (i.e., tLyp1-HA and neuropilin receptors) [35]. Due to the remarkable importance of this layer, it is of crucial importance to quantify the polymer attachment to the NCs. Results showed that the amount of polymer attached was lower in the NC formulated by SE with 2 mg per g of oil (equivalent to 0.02 mmol per mol of oil) than the ones formulated by SD, ranging from 34 to 53 mg of polymer per g of oil (or, in molar ratio, ranging from 0.27 to 3.64 mmol per mol of oil). Among the NCs formulated by SD, no clear influence of PEGylation was observed on the polymer attachment. Of note, the highest polymer association was observed for SD-noPEG-PEG·PGA, with 53 mg per g of oil (equivalent to 3.64 mmol per mol of oil). It could be speculated that the polymer molecules that are not part of the NCs’ shell may be solubilized and/or form micelles in the suspending medium. In addition, complexation products between some components, especially polymers with counter-charged surfactants, might be formed, as it is described, for example, for CTAB and PGA [36]. Finally, a highly dense PEGylated NC surface (as in SE-*b-*PEG-HA) might hinder the electrostatic interactions between the polymers and the counter-charged surfactants present at the oil/water interphase.

PEG moieties play an important role in providing the NCs with stealth properties in terms of protein adsorption. PEG amount, length, and disposition were reported to affect the protein corona of nanosystems in terms of protein variety and amount [37]. SD-*l-*PEG-HA and SE-*b-*PEG-HA contained PEG moieties in their surfactants, while SD-noPEG-PEG·PGA presented PEG residues in its polymeric shell. PEG content was found to be higher when PEG moieties were placed on the surfactants than in the polyglutamic shell, being the PEG content 42, 159, and 188 mg of PEG per g of oil for SD-noPEG-PEG·PGA, SD-*l-*PEG-HA, and SE-*b*-PEG-HA, respectively. The higher PEG content of SE-*b*-PEG-HA and SD-*l-*PEG-HA suggests that these formulations will be less taken up by macrophages and will have longer circulating times than the other tested NCs.

The robustness of the isolation techniques can be also inferred through the AE results. The oil mass percentage in the isolated SE-*b-*PEG-HA, which was subjected to TFF, and SD-noPEG-PEG·PGA, which was ultracentrifuged, was close to 100%, suggesting there was no loss of NCs during both isolating procedures. However, this was not the case for the other three ultracentrifuged prototypes. There might be two main reasons for the reduced ultracentrifuge-based separation performance of these NCs. Firstly, since the separation of the NCs from the infranatant is manual, a partial supernatant aspiration is likely to occur, especially if the cream-like supernatant is not thick and tends to redisperse easily. Secondly, there may coexist NCs with smaller size or with a particle density closer to that of the separating media which, due to the bases of ultracentrifugation, will tend less to accumulate in the upper part of the centrifugation vial. However, in all the tested NCs, substance attachment resulted to be reproducible (< 20% standard deviation), meaning that these partial NC losses are systematic.

Taking all of this into consideration, the importance of reporting the real composition of NCs, considering the number of components that are part of nanostructure and those that may be solubilized or forming micelles in the medium, is clear. As discussed above, the majority of the works report only on the total amount of each component, independently if they are bound or unbound, and this can be totally misleading, making it difficult to draw clear conclusions. The composition of the five NCs evaluated in this work, once isolated, is detailed in Table 2.

**Table 2:** NCs’ composition after isolation

Formulation	Compound	Expected concentration (mg·mL^−1^)	Actual concentration (mg·mL^−1^) (mean ± StD) (*n* = 3)
SD-*l*-PEG-HA	dl-α-Tocopherol	18.00	12.99 ± 0.84
	d-α-Tocopherol polyethylene glycol 1000 succinate	5.33	3.09 ± 0.32
	Hyaluronic acid	2.66	0.47 ± 0.08
	Benzethonium	0.67	0.26 ± 0.03
SD-noPEG-HA	Medium-chain triglycerides*	20.40	10.55 ± 0.77
	Hyaluronic acid	3.33	0.47 ± 0.06
	Phosphatidylcholines**	5.00	0.42 ± 0.08
	Hexadecyltrimethylammonium	1.00	0.35 ± 0.08
SD-noPEG-PSA	Medium-chain triglycerides*	20.40	13.20 ± 1.60
	Phosphatidylcholines**	5.00	0.46 ± 0.07
	Polysialic acid	3.33	0.45 ± 0.04
	Hexadecyltrimethylammonium	1.00	0.31 ± 0.04
SE-*b*-PEG-HA	Medium-chain triglycerides*	59.00	55.01 ± 4.22
	Polysorbate 80	58.00	13.90 ± 5.26
	Macrogol 15-hydroxystearate***	2.50	0.69 ± 0.09
	Benzethonium	0.20	0.10 ± 0.02
	Hyaluronic acid	0.25	0.11 ± 0.01
SD-noPEG-PEG·PGA	dl-α-Tocopherol	18.00	17.07 ± 1.03
	PEG·PGA	2.67	0.91 ± 0.25
	DOTAP	0.67	0.59 ± 0.02

*SD-l-PEG-HA*, lineal-polyethylene glycol-containing hyaluronic acid nanocapsules formulated by solvent displacement; *SD-noPEG-HA*, non-PEGylated hyaluronic acid nanocapsules formulated by solvent displacement; *SD-noPEG-PSA*, non-PEGylated polysialic acid nanocapsules formulated by solvent displacement; *SE-b-PEG-HA*, self-emulsifying branched-polyethylene glycol-containing hyaluronic acid nanocapsules; *StD*, standard deviation. Solvent displacement nanocapsules with non-PEGylated surfactants and a shell of polyethylene glycol polyglutamic acid

*For quantification of the medium-chain triglycerides, a contribution of 25% of each quantified molecule was assumed; **Phosphatidylcholine concentration was corrected by its purity in the used batch of Epikuron™ 145 V (51.7%); ***Macrogol 15-hydroxystearate concentration was corrected by its purity in Kolliphor® HS15 (70%)

## Conclusions

A comprehensive methodology enabling the quantification of the components of five different NCs elaborated by solvent displacement of self-emulsification techniques was achieved. Despite their high chemical diversity, we have shown that it is possible to quantify different oils, surfactants, and polymers using only one LC–MS/MS equipment and two chromatographic columns. Association profiles were found to be highly formulation-specific, especially for PEG presence and polymer attachment. Finally, the exhaustive composition characterization achieved by this work opens a window for a deeper in vitro and in vivo data understanding, as well as for a wider rational design of polymeric nanocapsules.

## Supplementary Information

Below is the link to the electronic supplementary material.

Supplementary file1 (PDF 4526 KB)

## Data Availability

Validation of the calibration curves for each molecule and formulation; chromatographic methods and chromatograms; detailed mass spectrometry methods and MS and MS/MS spectra; and detected mass percentages in the non-isolated formulation, retentate/supernatant, and permeate/infranatant are presented in the Supporting Information section (PDF).
