# Supplementary material for: Quantification of the actual composition of polymeric nanocapsules: a quality control analysis
Source: Drug Deliv Transl Res. 2022 Mar 18;12(11):2865–74. doi: 10.1007/s13346-022-01150-5 (PMC9512864; doi:10.1007/s13346-022-01150-5)
Supplement: Supplementary file 1 — Supplementary file1 (PDF 4526 KB) [file 13346_2022_1150_MOESM1_ESM.pdf]

SUPPORTING INFORMATION | Quantification of the actual composition of polymeric nanocapsules. A quality control analysis

Supplementary Table 1. Validation of the calibration curves for each quantified substance and formulation

Formulation	Molecule	RT (min) (mean) (n=3)	Calibration curve range (ppb)	r^2 (mean ± StD) (n=3)	Precision (pooled RStD%) (%)	Accuracy (RE%) (%) (low; mid; high range)	LoQ (ppb) (mean ± StD) (n=3)
SD-*l*-PEG-HA	Benzethonium chloride	2.6	8.0 – 80.0	0.9959 ± 0.0016	4.4	3.9; -0.3; 2.4	0.70 ± 0.38
	TPGS	4.0	64.0 – 640.0	0.9854 ± 0.0040	8.6	1.9; -1.0; 2.5	1.90 ± 0.86
	DL-α-tocopherol	4.9	216.0 – 2160.0	0.9948 ± 0.0040	4.9	-1.6; 2.6; 0.0	1.16 ± 0.47
	Hyaluronic acid (whole)	4.9	19,590.0 – 68,570.0	0.9897 ± 0.0009	4.0	3.5; 1.0; 1.0	22.51 ± 9.16
	Hyaluronic acid (supernatant)	4.9	15,670.0 – 78,370.0	0.9889 ± 0.0039	4.3	5.3; 3.6; 3.6	26.65 ± 6.62
	Hyaluronic acid (infranatant)	4.9	17,140.0 – 58,780.0	0.9840 ± 0.0056	4.8	5.6; 1.7; 1.7	34.42 ± 14.95
SD-noPEG-HA	CTAB	1.6	43.2 – 144.0	0.9985 ± 0.0009	1.0	2.0; -0.2; 1.1	0.27 ± 0.13
	C_8-C_8-C_8 Triglyceride	1.8	881.3 – 2937.6 (*)	0.9880 ± 0.0016	3.2	5.9; -2.7; 3.4	5.99 ± 2.76
	C_8-C_8-C_{10} Triglyceride	1.9	881.3 – 2937.6 (*)	0.9859 ± 0.0040	3.0	6.1; -2.0; 4.3	8.22 ± 2.40
	C_8-C_{10}-C_{10} Triglyceride	2.0	881.3 – 2937.6 (*)	0.9852 ± 0.0031	3.7	5.6; -3.5; 3.7	8.18 ± 1.66
	C_{10}-C_{10}-C_{10} Triglyceride	2.0	881.3 – 2937.6 (*)	0.9792 ± 0.0036	5.0	4.2; -2.7; 3.1	1.63 ± 0.31
	Phosphatidylcholine 756.6 Da	2.0	216.0 – 720.0 (**)	0.9887 ± 0.0074	1.6	2.8; 5.4; -4.7	0.62 ± 0.44
	Phosphatidylcholine 758.6 Da	2.0	216.0 – 720.0 (**)	0.9812 ± 0.0018	3.4	4.2; 5.3; -4.1	0.14 ± 0.01
	Phosphatidylcholine 780.6 Da	2.0	216.0 – 720.0 (**)	0.9908 ± 0.0052	1.2	1.8; 4.8; -4.2	0.24 ± 0.19
	Phosphatidylcholine 782.5 Da	2.1	216.0 – 720.0 (**)	0.9880 ± 0.0086	1.2	2.6; 6.1; -4.9	0.06 ± 0.01
	Hyaluronic acid (whole)	4.9	6,120.0 – 10,200.0	0.9383 ± 0.0652	4.0	3.7; 1.3; 1.3	19.18 ± 2.49
	Hyaluronic acid (supernatant)	4.9	20,000.0 – 98,000.0	0.9876 ± 0.0039	4.8	8.1; 6.0; 6.0	32.28 ± 9.41
	Hyaluronic acid (infranatant)	4.9	10,000.0 – 29,000.0	0.9852 ± 0.0052	4.3	1.8; 2.4; 2.4	15.35 ± 1.82
SD-noPEG-PSA	CTAB	1.6	43.2 – 144.0	0.9952 ± 0.0015	1.5	3.7; -0.2; 0.5	0.20 ± 0.01
	C_8-C_8-C_8 Triglyceride	1.8	881.3 – 2937.6 (*)	0.9814 ± 0.0025	3.3	8.5; -1.6; 2.5	6.46 ± 1.65
	C_8-C_8-C_{10} Triglyceride	1.9	881.3 – 2937.6 (*)	0.9801 ± 0.0017	3.8	8.4; -2.1; 2.8	9.44 ± 2.86
	C_8-C_{10}-C_{10} Triglyceride	2.0	881.3 – 2937.6 (*)	0.9848 ± 0.0008	4.0	5.2; -0.8; 2.3	7.59 ± 1.38
	C_{10}-C_{10}-C_{10} Triglyceride	2.0	881.3 – 2937.6 (*)	0.9697 ± 0.0165	5.9	4.6; -2.8; 3.1	1.08 ± 0.05
	Phosphatidylcholine 756.6 Da	2.0	216.0 – 720.0 (**)	0.9611 ± 0.0100	2.9	7.8; 4.7; -11.6	0.91± 0.14
	Phosphatidylcholine 758.6 Da	2.0	216.0 – 720.0 (**)	0.9552 ± 0.0114	4.7	8.1; 3.2; -11.9	0.15± 0.02
	Phosphatidylcholine 780.6 Da	2.0	216.0 – 720.0 (**)	0.9720 ± 0.0092	1.7	7.1; 4.8; -9.1	0.31 ± 0.14
	Phosphatidylcholine 782.5 Da	2.1	216.0 – 720.0 (**)	0.9628 ± 0.0116	2.2	8.0; 4.8; -11.8	0.08 ± 0.01
	Polysialic acid (whole)	5.1	6,150.0 – 10,870.0	0.9788 ± 0.0115	1.4	2.4; 0.7; 0.7	86.30 ± 32.47
	Polysialic acid (supernatant)	5.1	4,900.0 – 81,630.0	0.9867 ± 0.0061	2.8	3.6; 12.8; 12.8	413.54 ± 424.46
	Polysialic acid (infranatant)	5.1	7,350.0 – 29,390.0	0.9929 ± 0.0054	1.7	3.6; 3.6; 3.6	245.55 ± 134.63

Supplementary Table 1. Validation of the calibration curves for each quantified substance and formulation (continuation)

Formulation	Molecule	RT (min) (mean) (n=3)	Calibration curve range (ppb)	r^2 (mean ± StD) (n=3)	Precision (pooled RStD%) (%)	Accuracy (RE%) (%) (low; mid; high range)	LoQ (ppb) (mean ± StD) (n=3)
SE-*b*-PEG-HA	Benzethonium chloride	2.5	1.0 – 9.6	0.9997 ± 0.0002	1.1	0.5; -4.7; 0.1	0.24 ± 0.03
	Macrogol-15-hydroxystearate	3.2	6.0 – 120.0 (***)	0.9992 ± 0.0001	2.0	-1.8; -0.6; -1.5	2.25 ± 1.74
	Polysorbate 80	4.4	487.2 – 2784.0	0.9990 ± 0.0002	0.8	3.9; -1.6; 1.4	11.61 ± 0.36
	C_8-C_8-C_8 Triglyceride	8.3	849.6 – 2832.0 (*)	0.9939 ± 0.0028	2.5	3.0; -2.1; 1.1	3.32 ± 1.59
	C_8-C_8-C_{10} Triglyceride	8.7	849.6 – 2832.0 (*)	0.9895 ± 0.0027	3.8	3.9; -1.5; 1.0	2.51 ± 1.14
	C_8-C_{10}-C_{10} Triglyceride	8.9	849.6 – 2832.0 (*)	0.9895 ± 0.0030	3.7	4.8; 4.7; 0.7	2.15 ± 1.82
	C_{10}-C_{10}-C_{10} Triglyceride	9.0	849.6 – 2832.0 (*)	0.9889 ± 0.0046	3.6	2.5; -4.4; 2.1	1.88 ± 1.17
	Hyaluronic acid (whole)	4.9	6,730.0 – 17,140.0	0.9863 ± 0.0004	2.4	3.2; 1.7; 1.7	15.53 ± 6.42
	Hyaluronic acid (retentate)	4.9	2,450.0 – 9,180.0	0.9658 ± 0.0164	3.3	8.7; 8.4; 8.4	14.21 ± 5.29
	Hyaluronic acid (permeate)	4.9	7,000.0 – 28,000.0	0.9901 ± 0.0049	3.9	4.0; 1.1; 1.1	35.22 ± 3.82
SD-noPEG-PEG·PGA	DL-α-tocopherol	2.9	288.8 – 2,887.5	0.9958 ± 0.0009	2.5	-7.5; 2.8; -3.5	6.33 ± 0.58
	1,2-dioleoyl-3-trimethylammoniumpropane chloride	3.6	7.5 – 75.0	0.9984 ± 0.0010	1.7	-2.5; -0.3; -0.8	1.26 ± 0.49
	PEG·PGA (whole)	9.8	23,700 – 132,200	0.9978 ± 0.0012	1.1	3.8; 1.0; 1.0	432.10 ± 360.61
	PEG·PGA (supernatant)	9.8	7,700 – 184,800	0.9976 ± 0.0017	1.3	2.8; 4.6; 4.6	666.06 ± 970.38
	PEG-PGA (infranatant)	9.8	4,800 – 113,300	0.9984 ± 0.0012	1.9	2.5; 1.1; 1.1	69.37 ± 39.55

SD-*l*-PEG-HA: lineal-polyethylene glycol-containing hyaluronic acid nanocapsules formulated by solvent displacement. SD-noPEG-HA: non-PEGylated hyaluronic acid nanocapsules formulated by solvent displacement. SD-noPEG-PSA: non-PEGylated polysialic acid nanocapsules formulated by solvent displacement. SE-*b*-PEG-HA: Self-emulsifying branched-polyethylene glycol-containing hyaluronic acid nanocapsules. SD-noPEG-PEG·PGA: Solvent displacement nanocapsules not containing PEGylated surfactants and with an outer shell of polyethylene glycol polyglutamic acid. StD: standard deviation. RT: retention time. RStD%: Relative standard deviation of the responses for each concentration level. RE%: relative differences between the estimated concentrations and the actual standard concentration. LoQ: limit of quantification. TPGS: D-α-tocopherol polyethylene glycol 1000 succinate. CTAB: Hexadecyltrimethylammonium bromide. (*): C_8-C_8-C_8, C_8-C_8-C_{10}, C_8-C_{10}-C_{10}, and C_{10}-C_{10}-C_{10} triglycerides calibration curves were built based on the amount of Miglyol® 812N. (**): Phosphatidylcholines' calibration curves were built based on the amount of Epikuron™ 145V. (***): macrogol-15-hydroxystearate calibration curves were built based on the amount of Kolliphor® HS15.

Supplementary Table 2. Chromatographic method for the analysis of non-polymeric substances

Formulation	Time (min)	A (%)	B (%)	C (%)	D (%)
SD-*l*-PEG-HA	0.00	70	30	0	0
	1.00	70	30	0	0
	1.10	0	100	0	0
	6.90	0	100	0	0
	7.00	0	0	100	0
	10.40	0	0	100	0
	10.50	70	30	0	0
	12.00	70	30	0	0
SD-noPEG-HA and SD-noPEG-PSA	0.00	90	10	0	0
	0.10	90	10	0	0
	0.11	0	40	0	60
	2.90	0	40	0	60
	2.95	0	0	100	0
	4.00	0	0	100	0
	4.05	90	10	0	0
	5.00	90	10	0	0
SE-*b*-PEG-HA	0.00	70	30	0	0
	0.20	70	30	0	0
	0.30	40	60	0	0
	5.30	24	76	0	0
	6.80	0	100	0	0
	8.50	0	30	0	70
	9.50	0	30	0	70
	9.60	0	0	100	0
	14.00	0	0	100	0
	14.10	70	30	0	0
	16.00	70	30	0	0
SD-noPEG-PEG·PGA	0.00	70	30	0	0
	1.00	70	30	0	0
	1.10	0	50	0	50
	3.00	0	50	0	50
	3.10	0	0	100	0
	4.50	0	0	100	0
	4.60	70	30	0	0
	6.00	70	30	0	0

A: 50 mM formic acid and 2 mM ammonium formate in water. B. 95 % acetonitrile and 5 % 2 mM ammonium formate aqueous solution (v/v) 50 mM formic acid. C: water. D: isopropyl alcohol. SD-*l*-PEG-HA: lineal-polyethylene glycol-containing hyaluronic acid nanocapsules formulated by solvent displacement. SD-noPEG-HA: non-PEGylated hyaluronic acid nanocapsules formulated by solvent displacement. SD-noPEG-PSA: non-PEGylated polysialic acid nanocapsules formulated by solvent displacement. SE-*b*-PEG-HA: Self-emulsifying branched-polyethylene glycol-containing hyaluronic acid nanocapsules.

Supplementary Table 3. Chromatographic method for the analysis of digested hyaluronic and polysialic acids

Time (min)	%A	%B
0.00	98	2
2.00	98	2
3.00	60	40
4.00	60	40
4.10	20	80
8.00	20	80
9.00	98	2
13.00	98	2

A: 50 mM formic acid and 2 mM ammonium formate in water, and B. methanol. The flow was diverted to waste from minute 0.00 to 3.90 to avoid the non-volatile salt Na_2SO_4 from entering the detector.

Supplementary Table 4. Chromatographic method for the analysis of digested PEG·PGA

Time (min)	%A	%B
0.00	0	100
6.00	0	100
6.10	5.9	94.1
10.00	17.6	82.4
12.00	29.4	70.6
12.10	0	100
15.50	0	100

A: 75 mM formic acid and 5 mM ammonium formate in water, and B: 75 mM formic acid and 5 mM ammonium formate in acetonitrile (90 %) and water (10 %) mixture (v/v). The flow was diverted to waste from minute 0.00 to 9.00 to avoid the non-volatile salt Na_2SO_4 from entering the detector.

Supplementary Table 5. Chromatograms of all the chromatographic methods and quantified molecules

Chromatographic method	Chromatogram
Hydrophobic substances of SD-*l*-PEG-HA	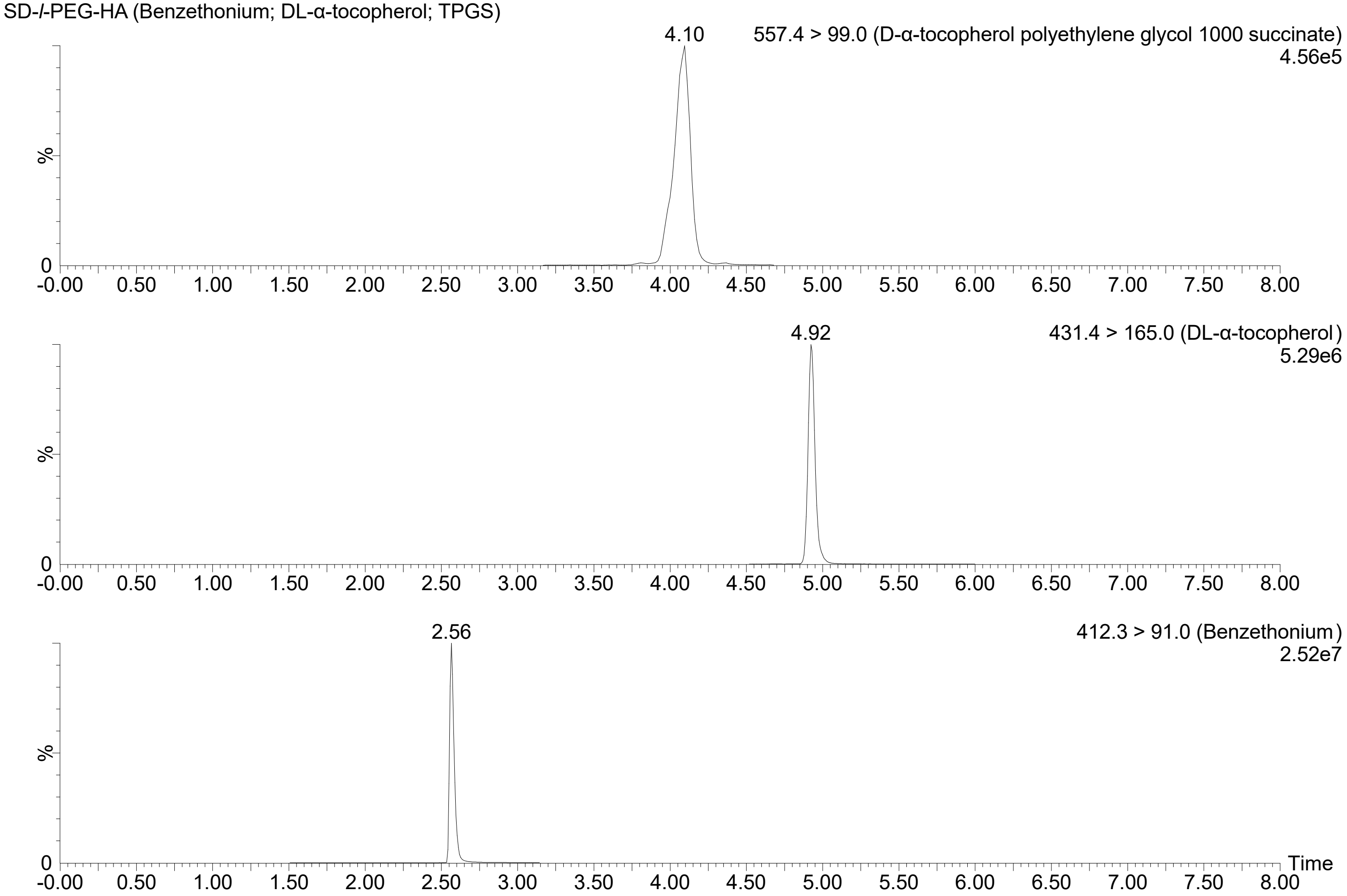

Supplementary Table 5. Chromatograms of all the chromatographic methods and quantified molecules (continuation)

Chromatographic event	Chromatogram
Hydrophobic substances of SD-noPEG-HA and SD-noPEG-PSA (run #1; CTAB and Miglyol® 812N)	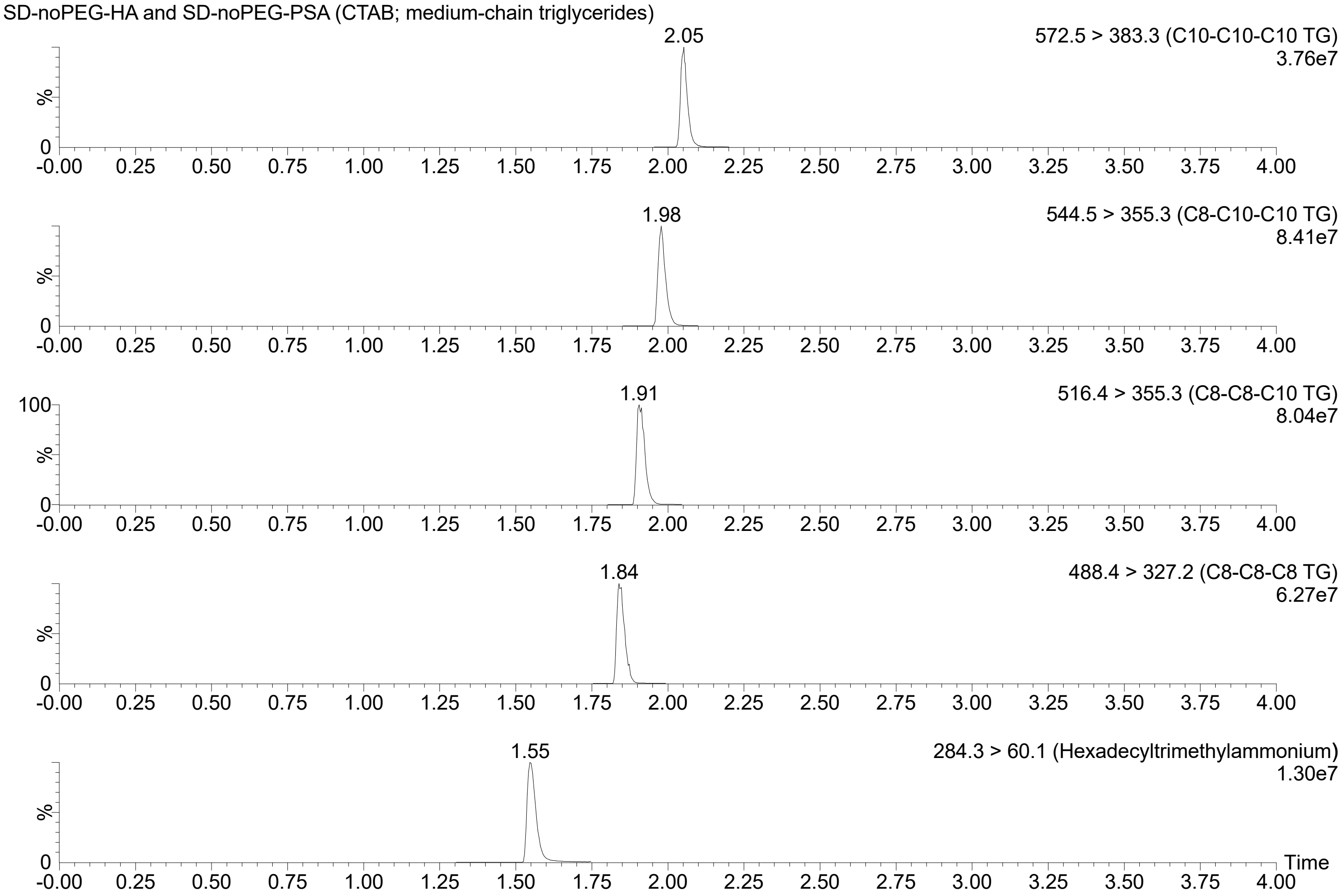

Supplementary Table 5. Chromatograms of all the chromatographic methods and quantified molecules (continuation)

Chromatographic event	Chromatogram
Hydrophobic substances of SD-noPEG-HA and SD-noPEG-PSA (run #2; Phosphatidylcholine)	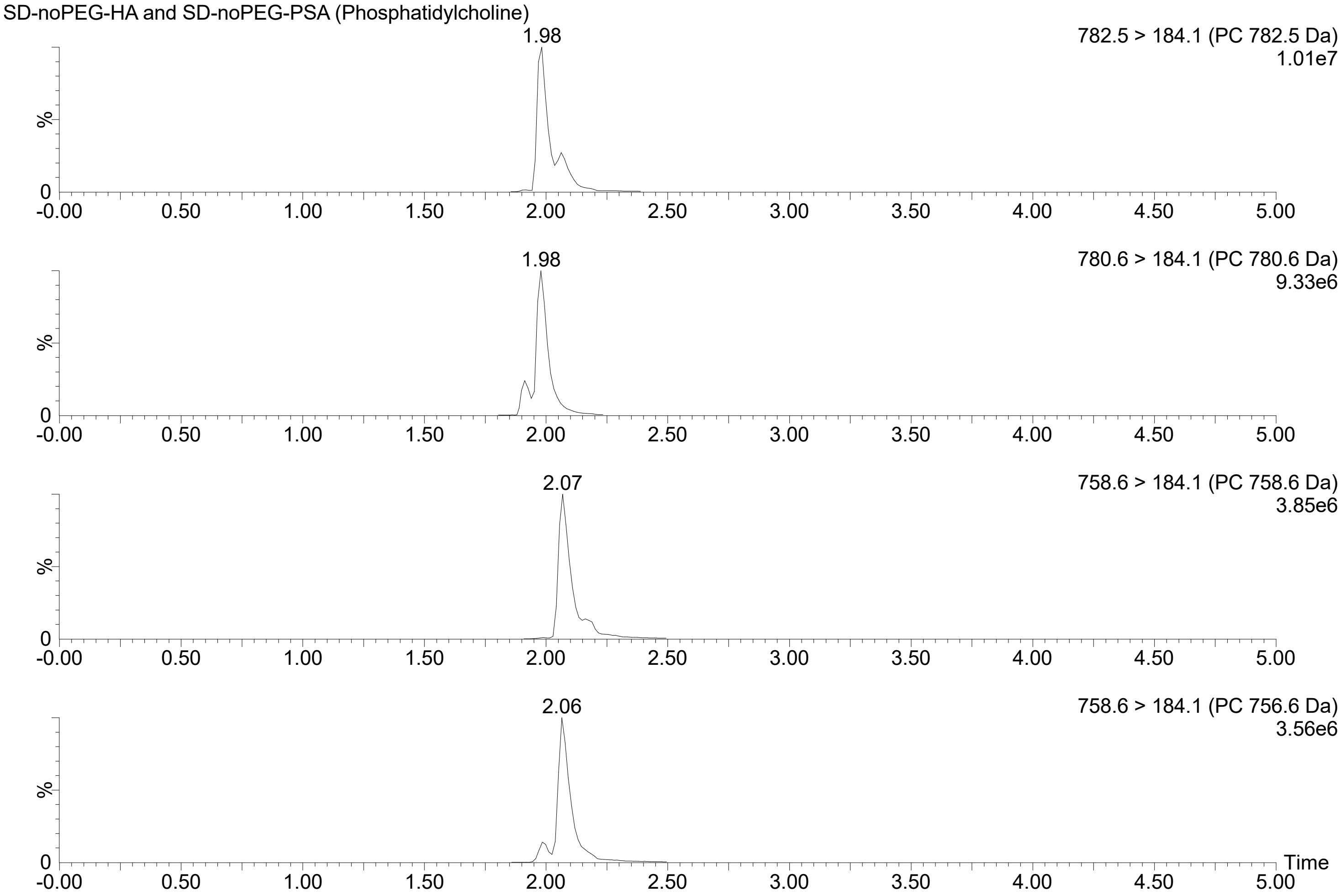

Supplementary Table 5. Chromatograms of all the chromatographic methods and quantified molecules (continuation)

Chromatographic event	Chromatogram
Hydrophobic substances of SE-*b*-PEG-HA	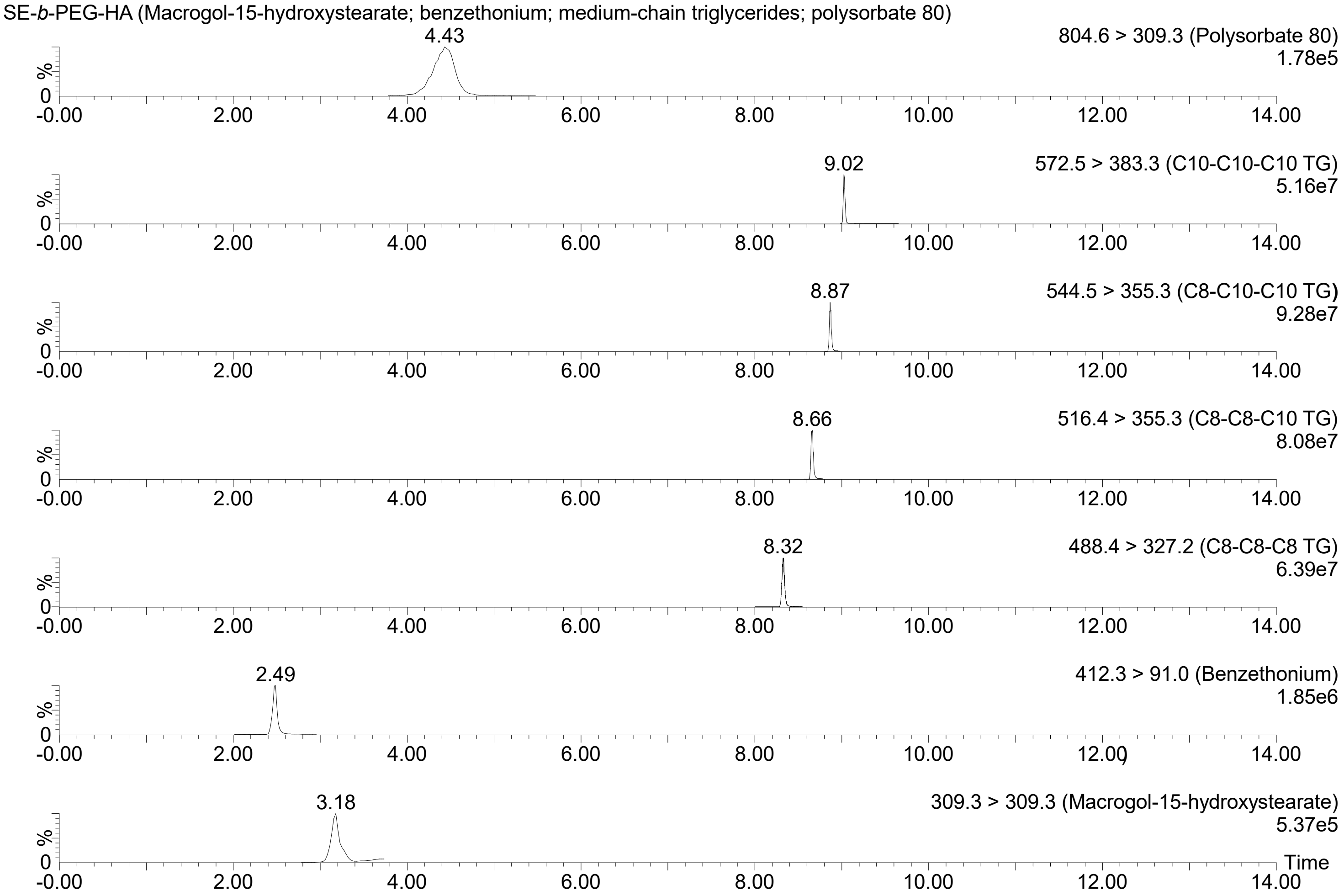

Supplementary Table 5. Chromatograms of all the chromatographic methods and quantified molecules (continuation)

Chromatographic event	Chromatogram
Hydrophobic substances of SD-noPEG-PEG·PGA	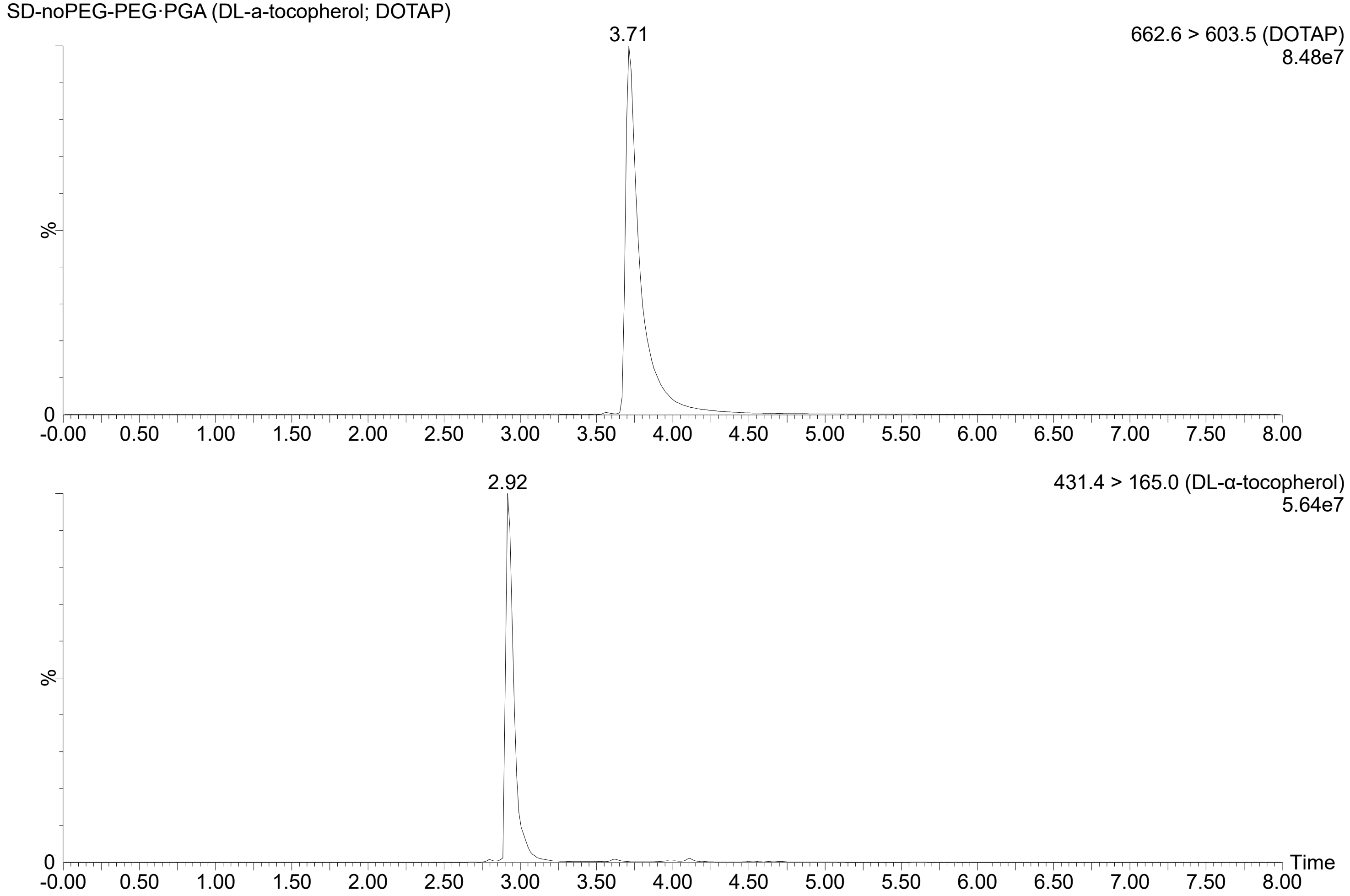

Supplementary Table 5. Chromatograms of all the chromatographic methods and quantified molecules (continuation)

Chromatographic event	Chromatogram
Digested hyaluronic acid	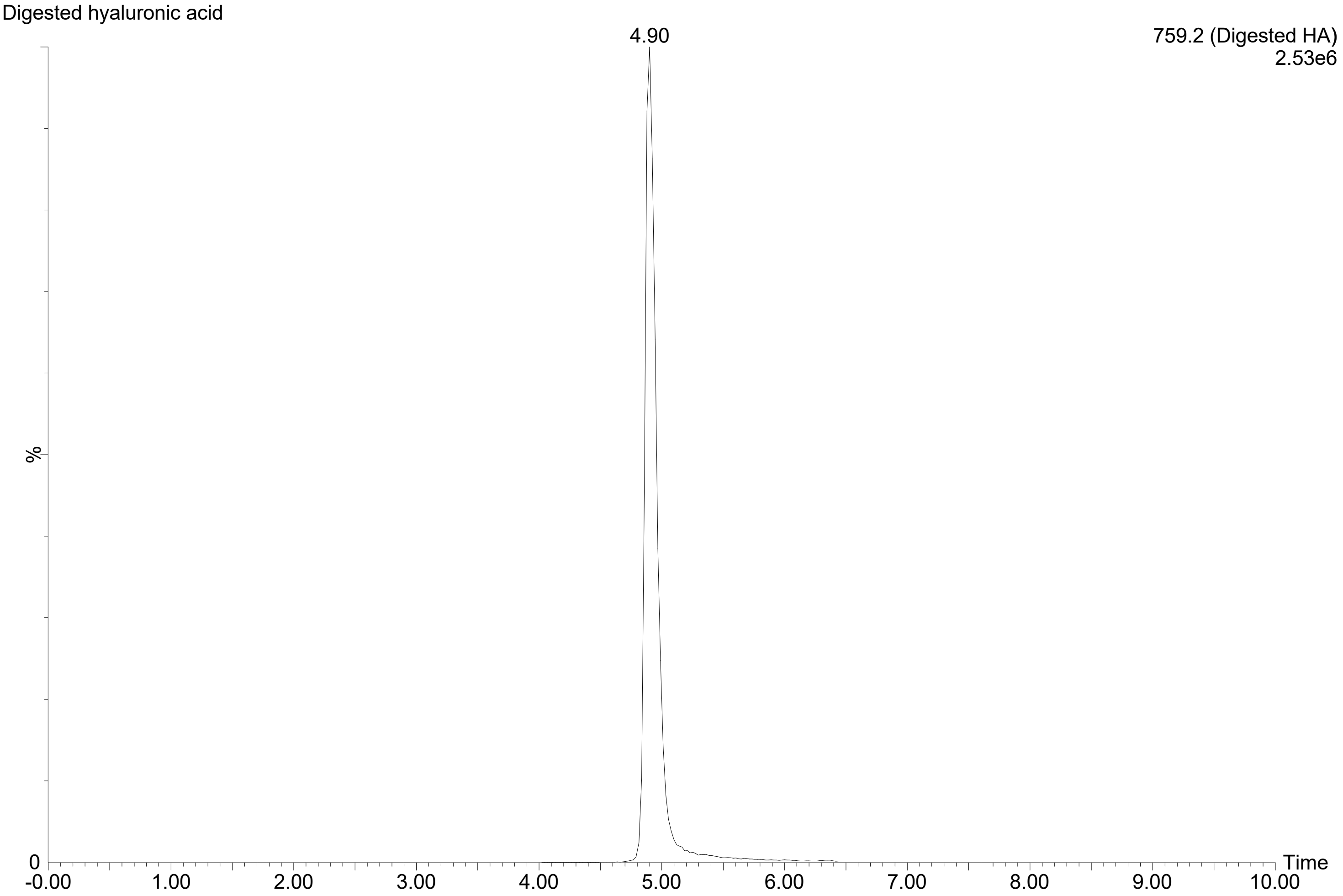

Supplementary Table 5. Chromatograms of all the chromatographic methods and quantified molecules (continuation)

Chromatographic event	Chromatogram
Digested polysialic acid	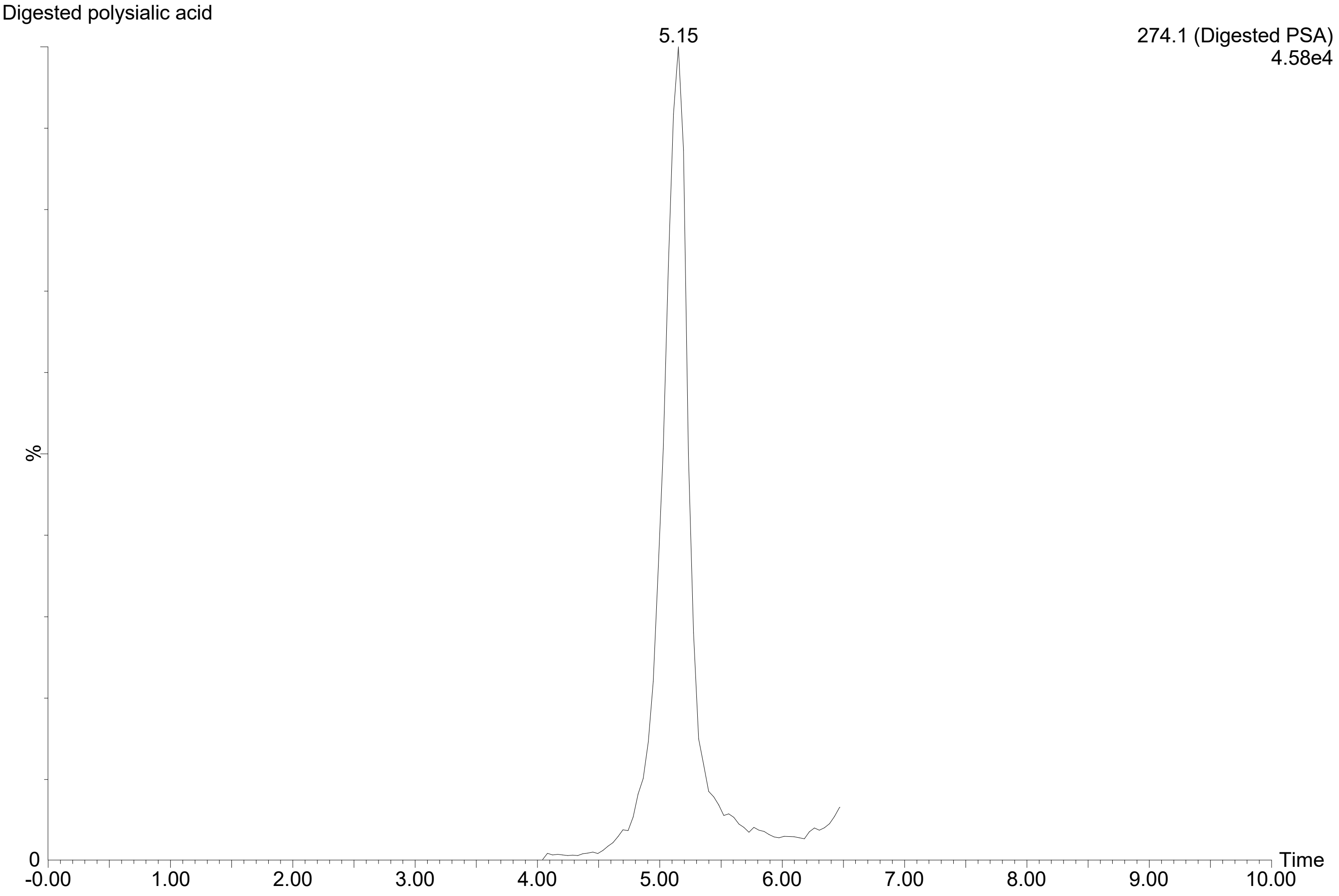

Supplementary Table 5. Chromatograms of all the chromatographic methods and quantified molecules (continuation)

Chromatographic event	Chromatogram
Digested polyethylene glycol poly-glutamic acid	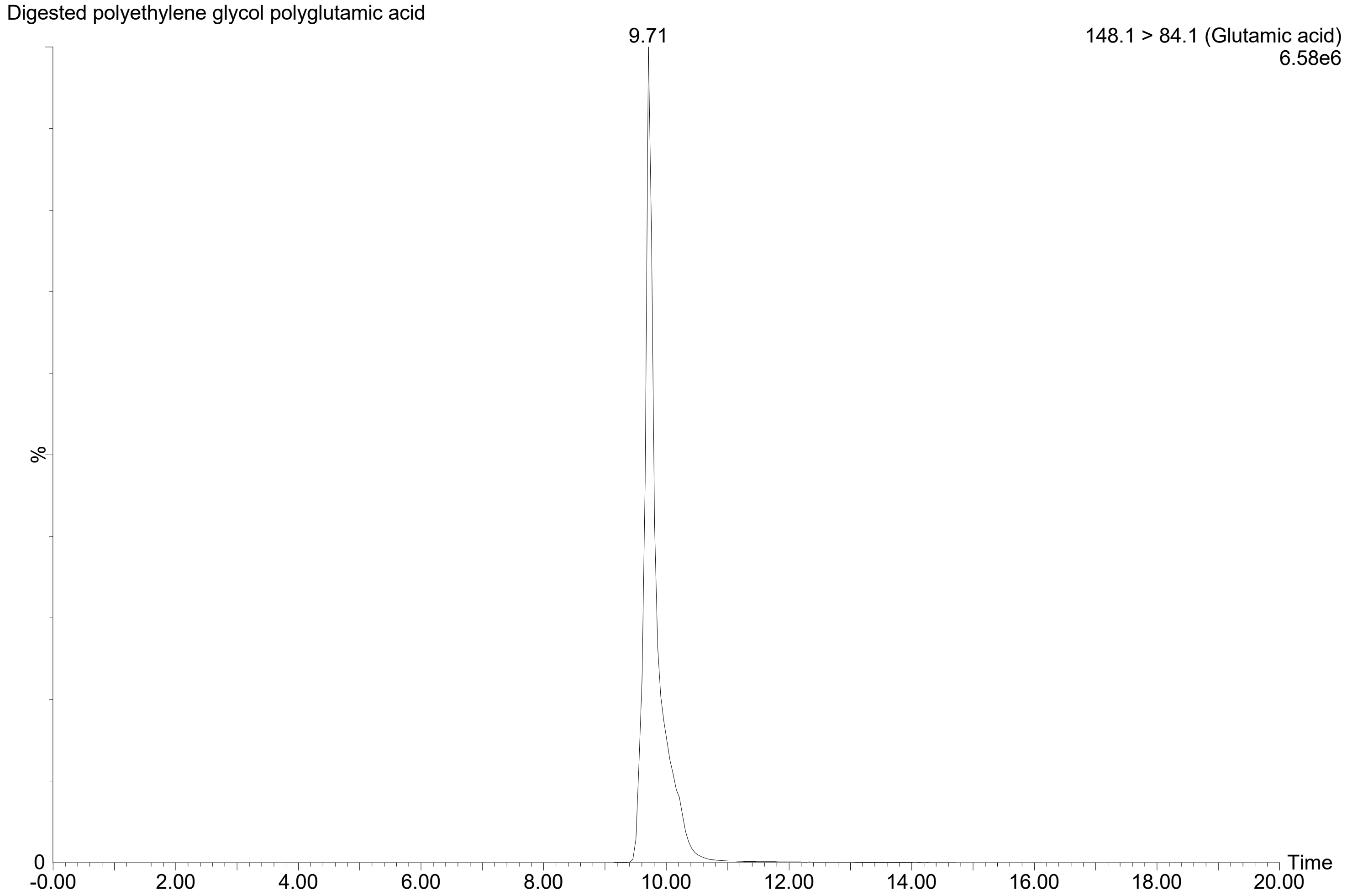

Supplementary Table 6. MS and MS/MS spectra for all the quantified substances

Substance	MS spectra	MS/MS spectra
DL-α-tocopherol	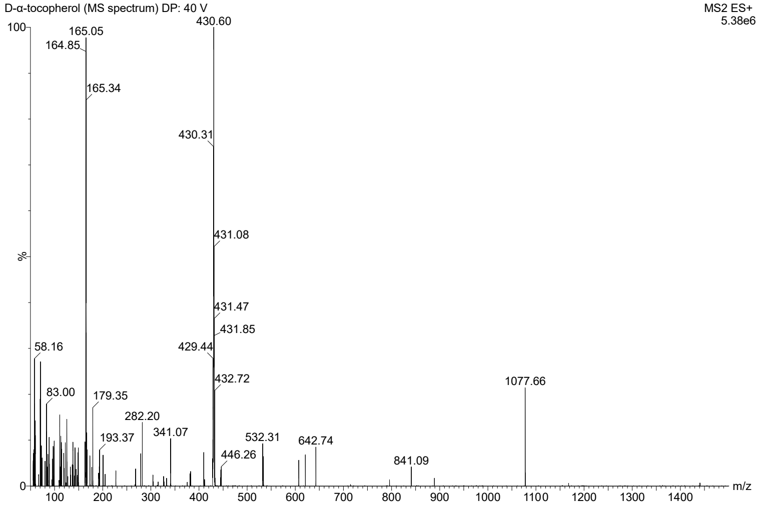	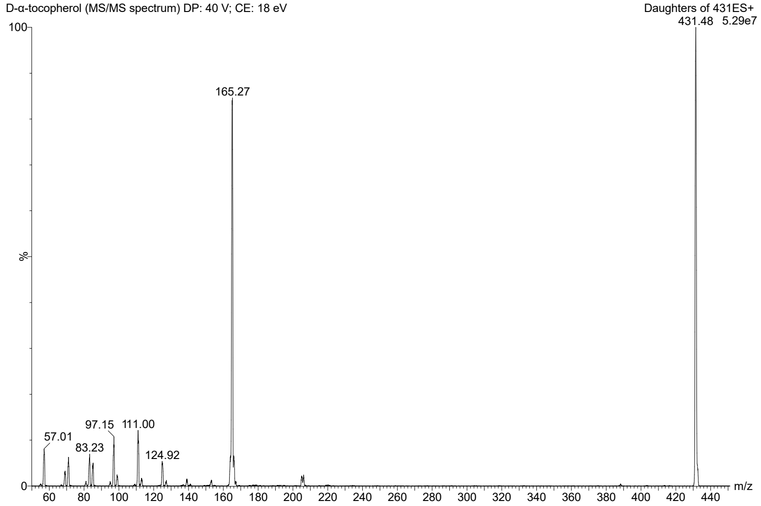
Benzethonium	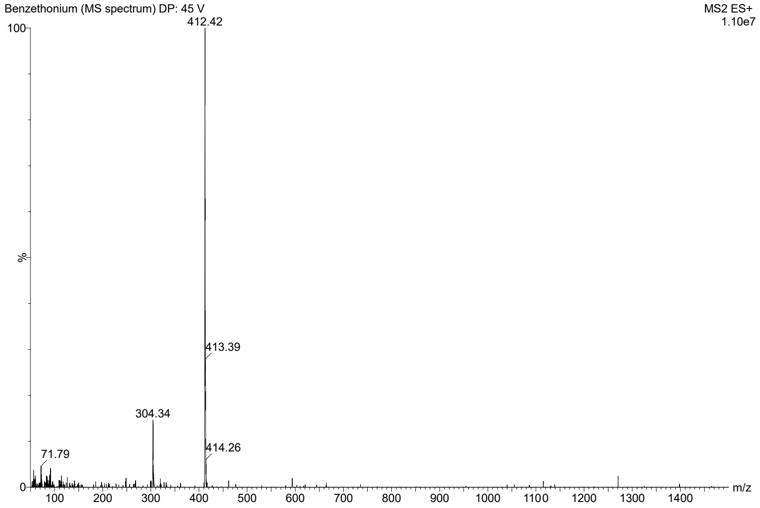	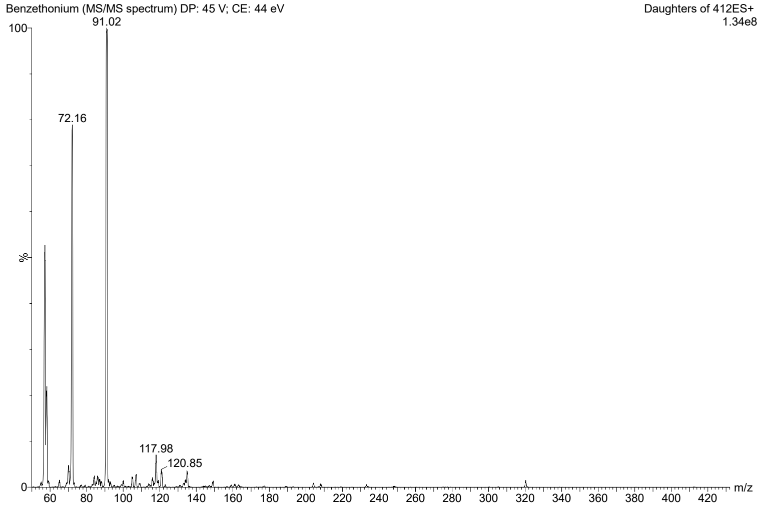

Supplementary Table 6. MS and MS/MS spectra for all the quantified substances (continuation)

Substance	MS spectra	MS/MS spectra
D-α-tocopherol polyethylene glycol 1000 succinate	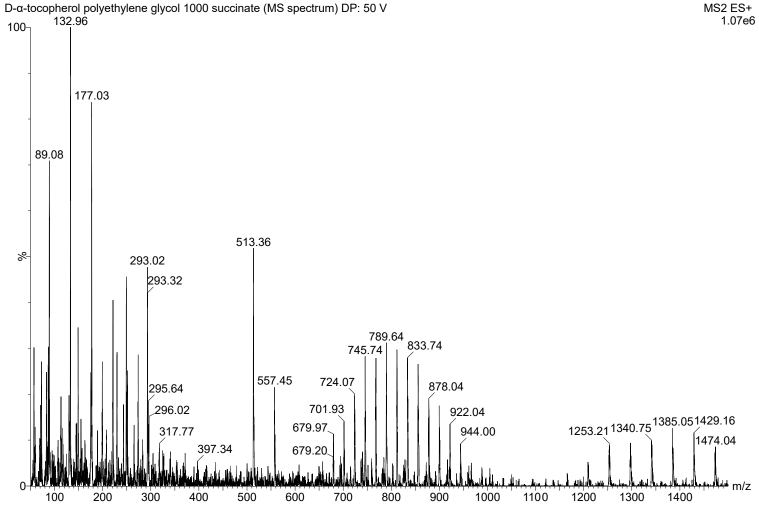	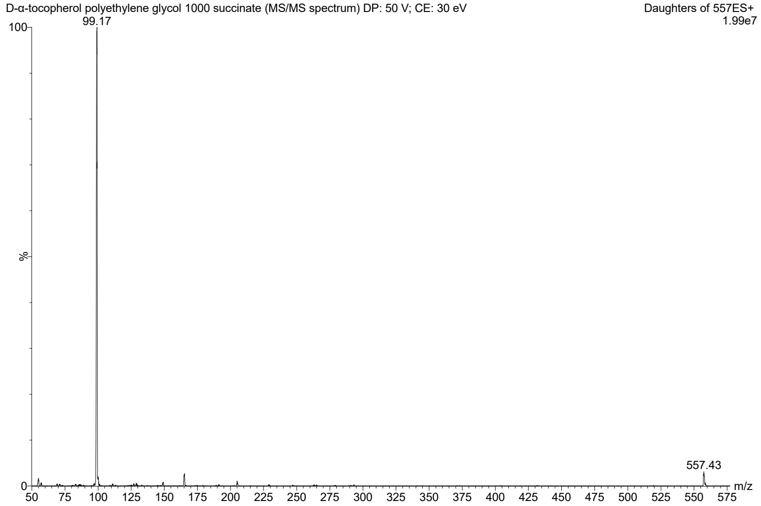
Hexadecyltrimethylammonium	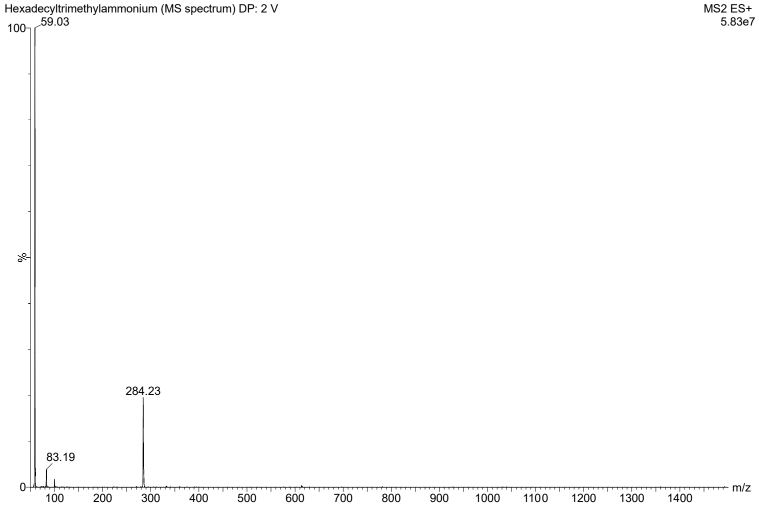	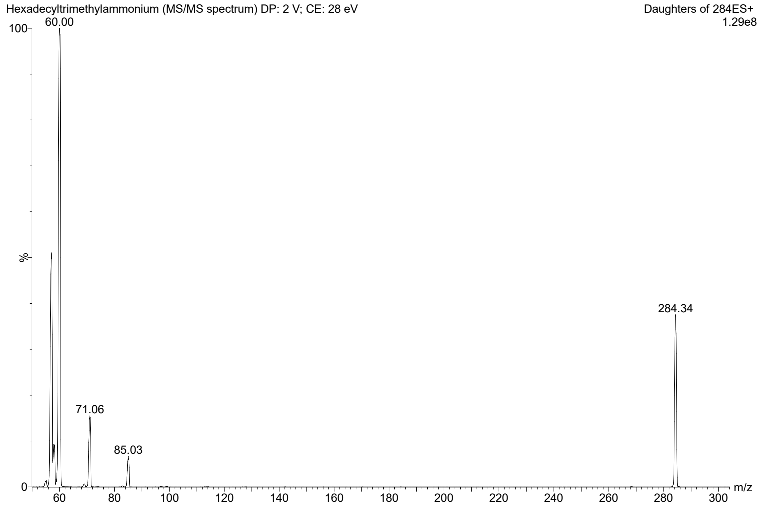

Supplementary Table 6. MS and MS/MS spectra for all the quantified substances (continuation)

Substance | MS spectra | MS/MS spectra

1,2-dioleoyl-3-trimethylammoniumpropane

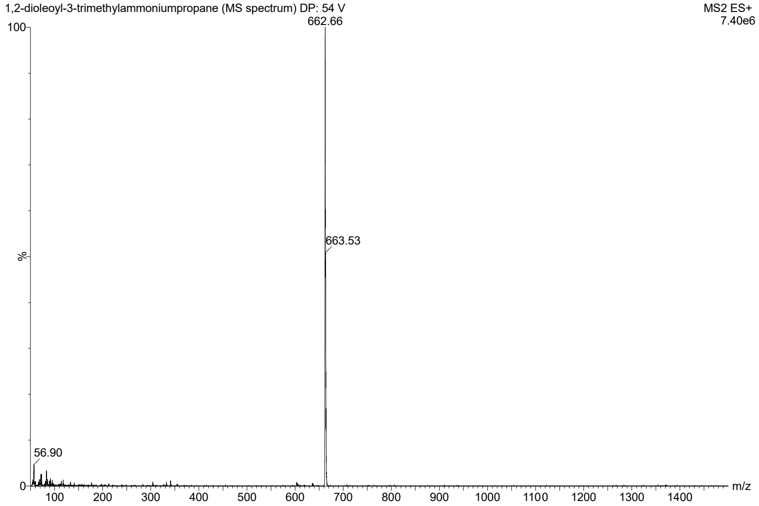

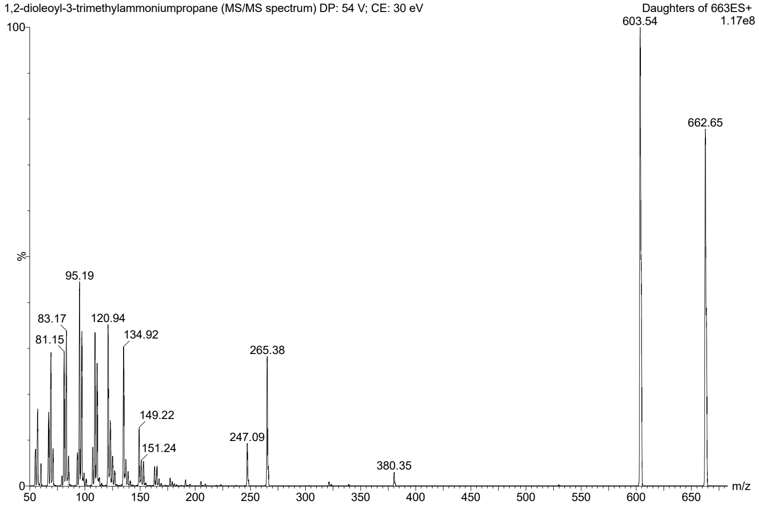

C_8-C_8-C_8 trigyleride (Miglyol® 812N)

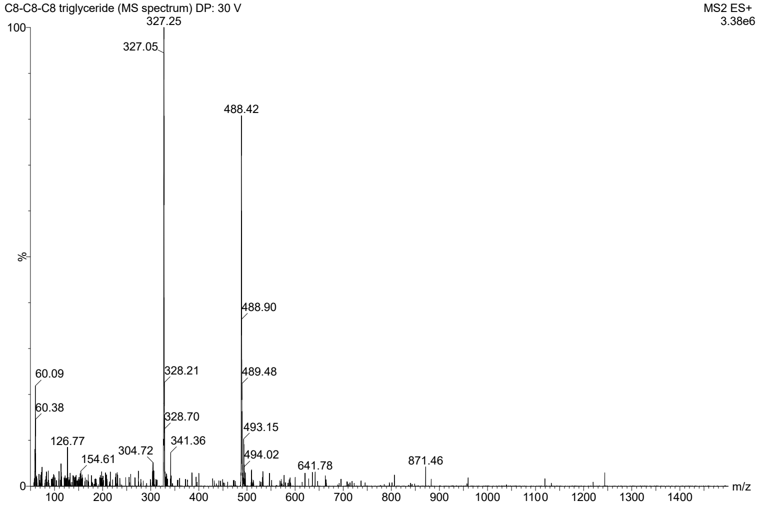

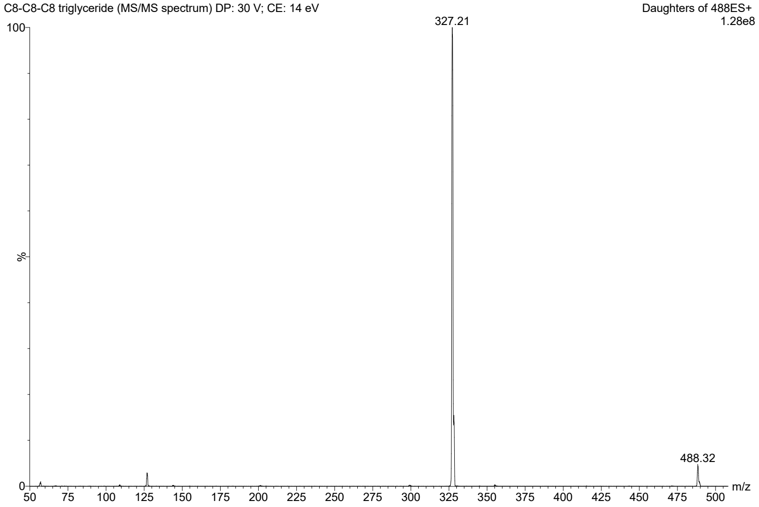

Supplementary Table 6. MS and MS/MS spectra for all the quantified substances (continuation)

Substance	MS spectra	MS/MS spectra
C_8-C_8-C_{10} trigyleride (Miglyol® 812N)	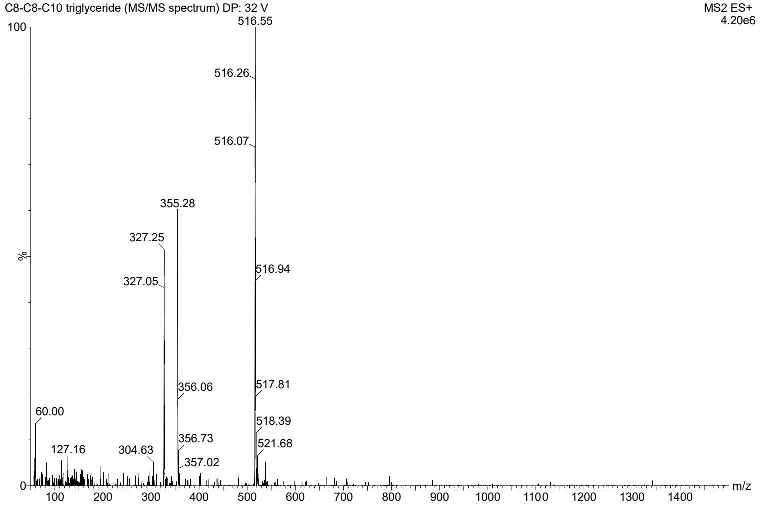	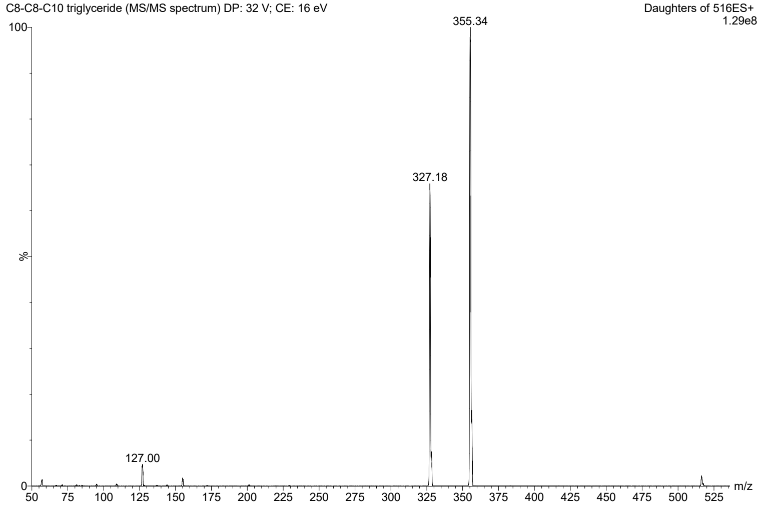
C_8-C_{10}-C_{10} trigyleride (Miglyol® 812N)	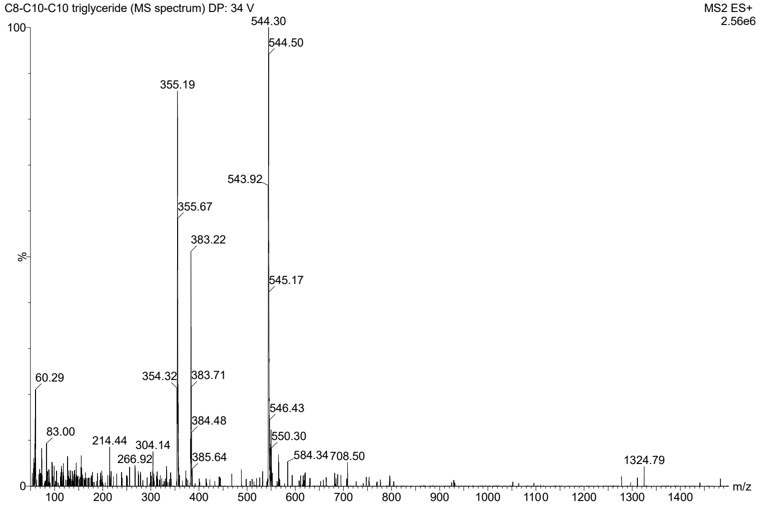	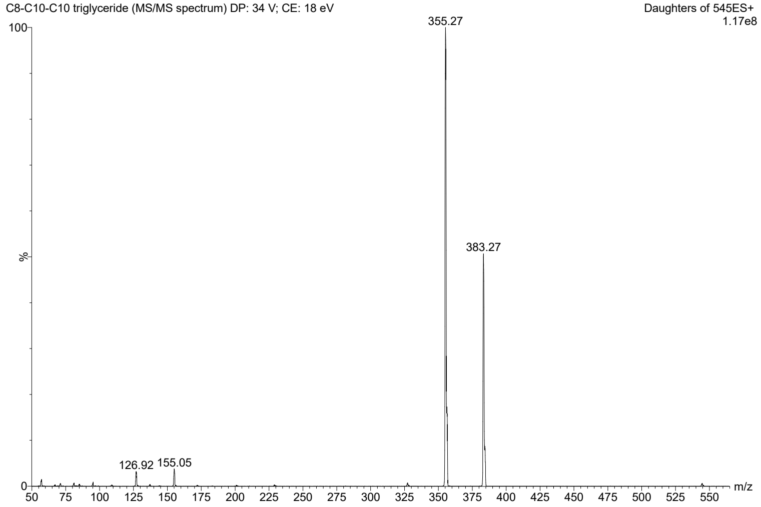

Supplementary Table 6. MS and MS/MS spectra for all the quantified substances (continuation)

Substance	MS spectra	MS/MS spectra
C_{10}-C_{10}-C_{10} trigyleride (Miglyol® 812N)	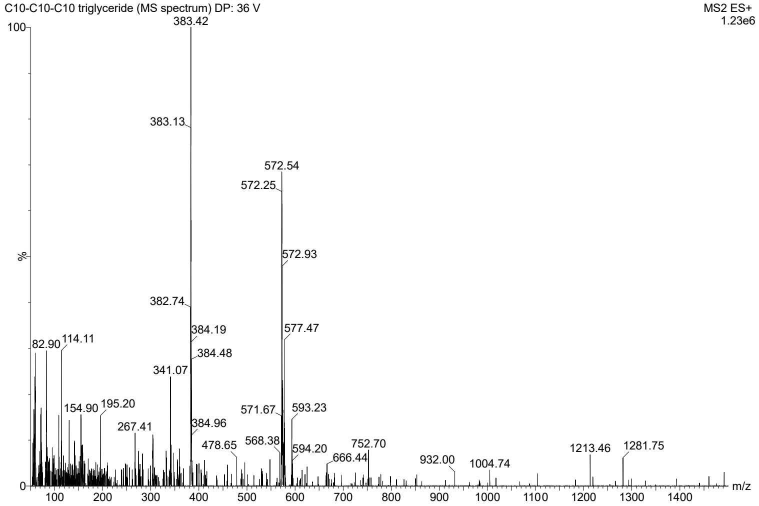	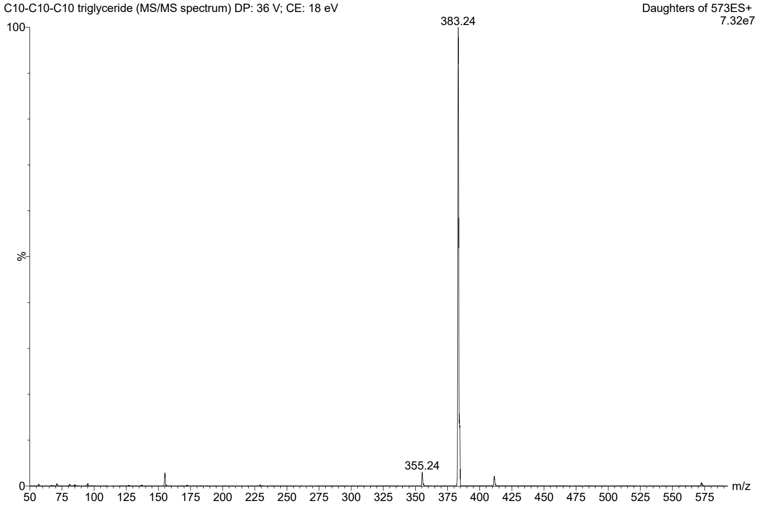
Phosphatidylcholine 756.5 Da	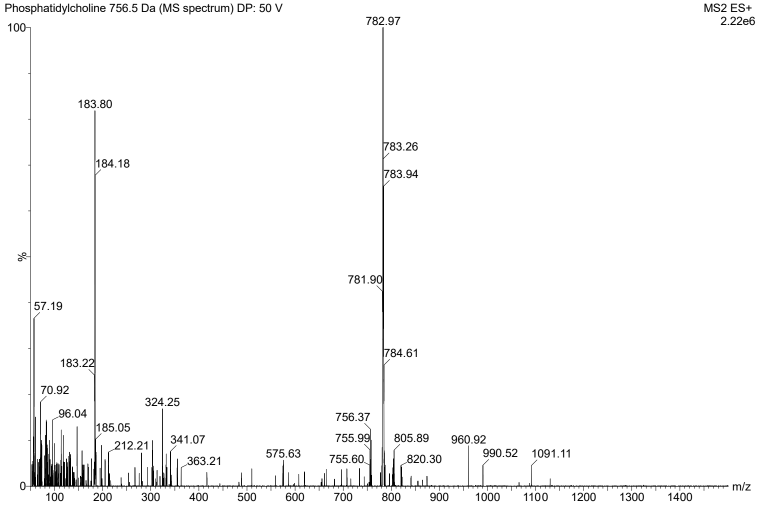	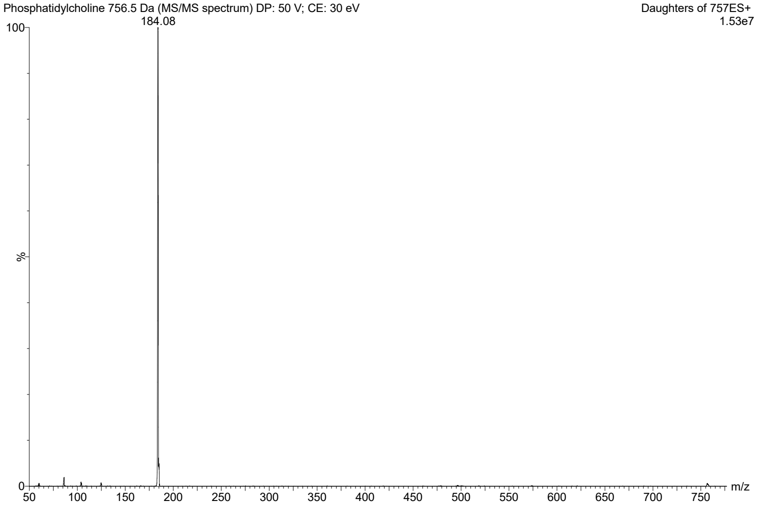

Supplementary Table 6. MS and MS/MS spectra for all the quantified substances (continuation)

<table>
<tr><th>Substance</th><th>MS spectra</th><th>MS/MS spectra</th></tr>
<tr><td>Phosphatidylcholine
758.5 Da</td><td>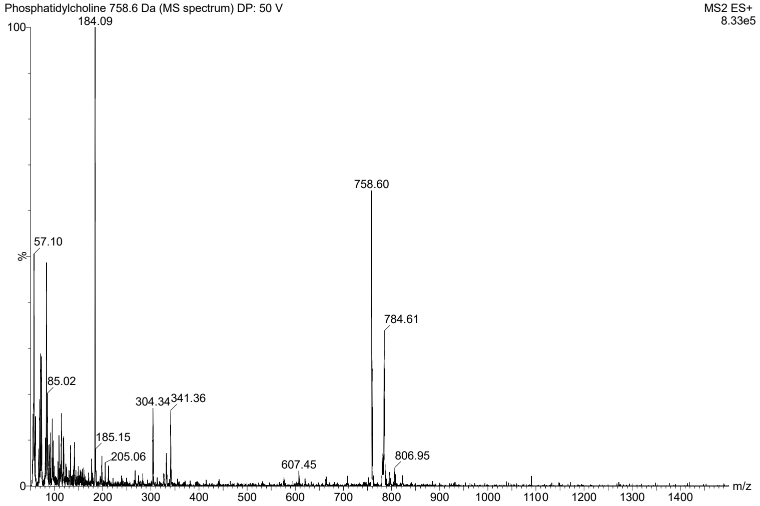
</td><td>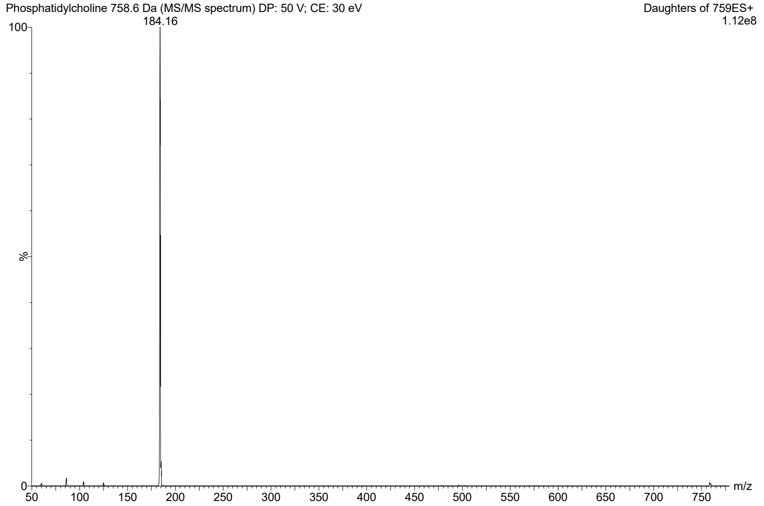
</td></tr>
<tr><td>Phosphatidylcholine
780.5 Da</td><td>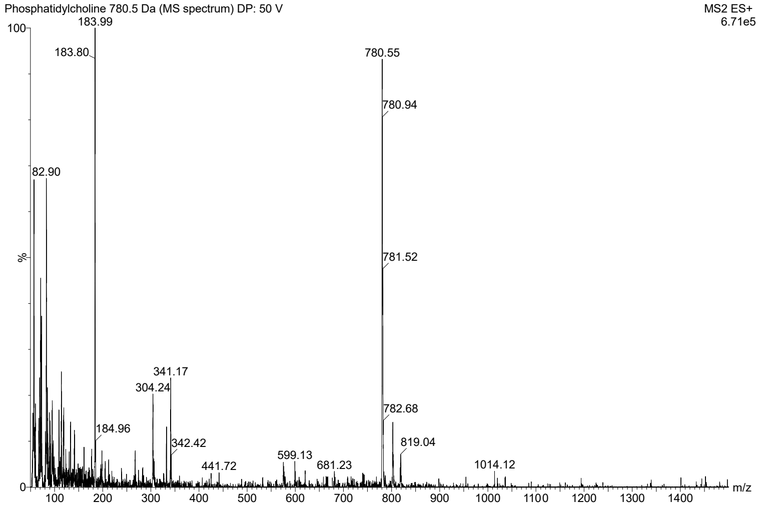
</td><td>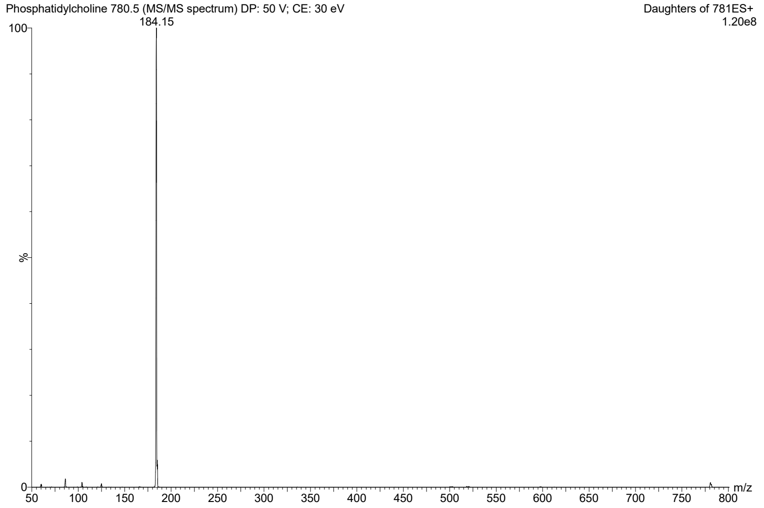
</td></tr>
</table>

Supplementary Table 6. MS and MS/MS spectra for all the quantified substances (continuation)

Substance	MS spectra	MS/MS spectra
Phosphatidylcholine 782.5 Da	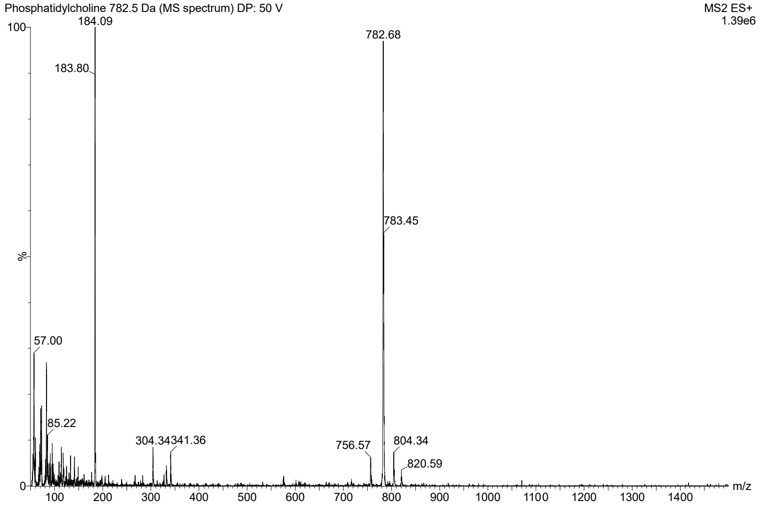	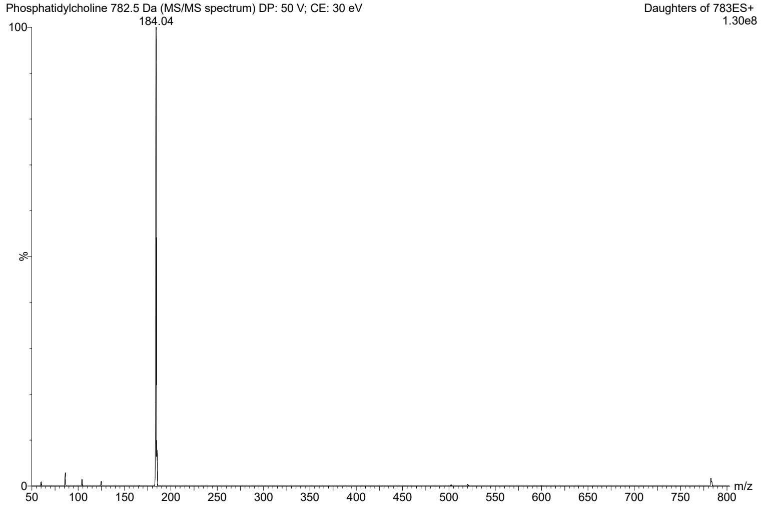
Macrogol-15-hydroxys-tearate	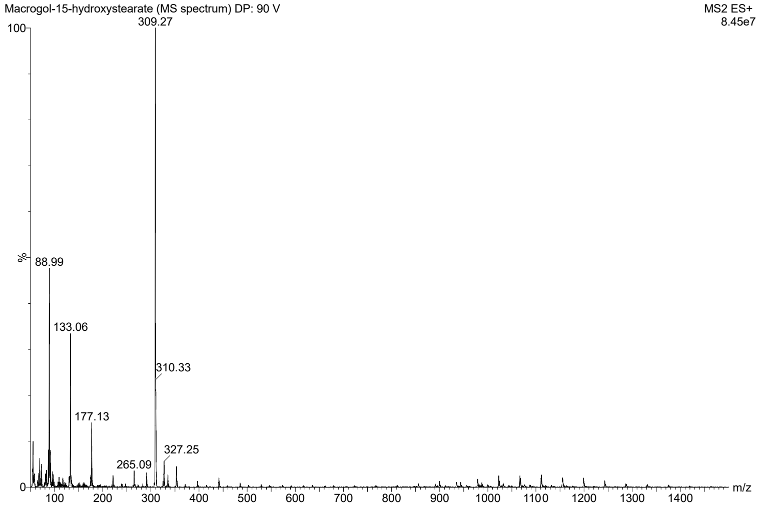	N/A

SUPPORTING INFORMATION | Quantification of the actual composition of polymeric nanocapsules. A quality control analysis

Supplementary Table 6. MS and MS/MS spectra for all the quantified substances (continuation)

Substance	MS spectra	MS/MS spectra
Polysorbate 80	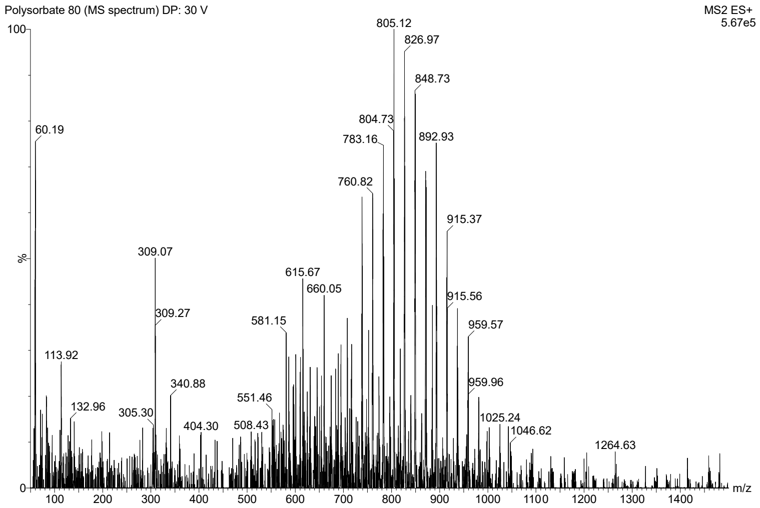	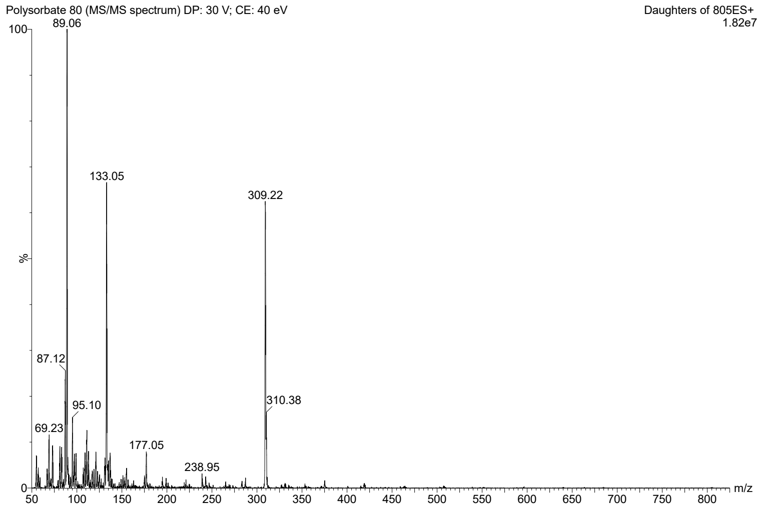
Digested hyaluronic acid	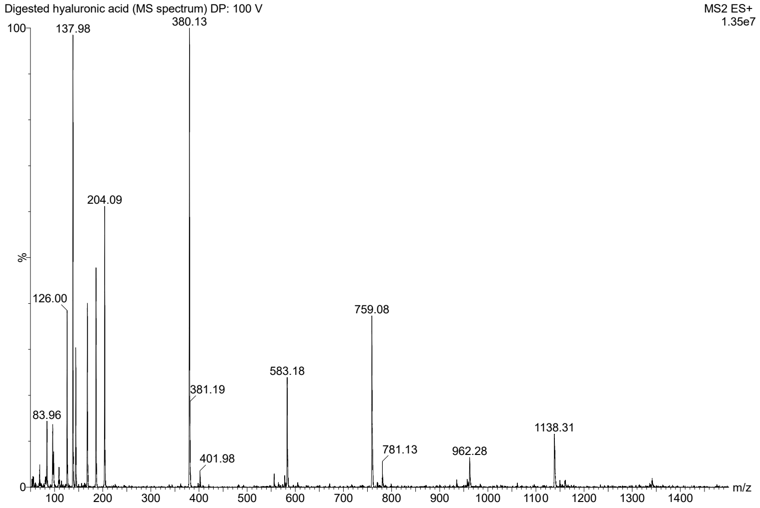	N/A

Supplementary Table 6. MS and MS/MS spectra for all the quantified substances (continuation)

Substance	MS spectra	MS/MS spectra
Digested polysialic acid	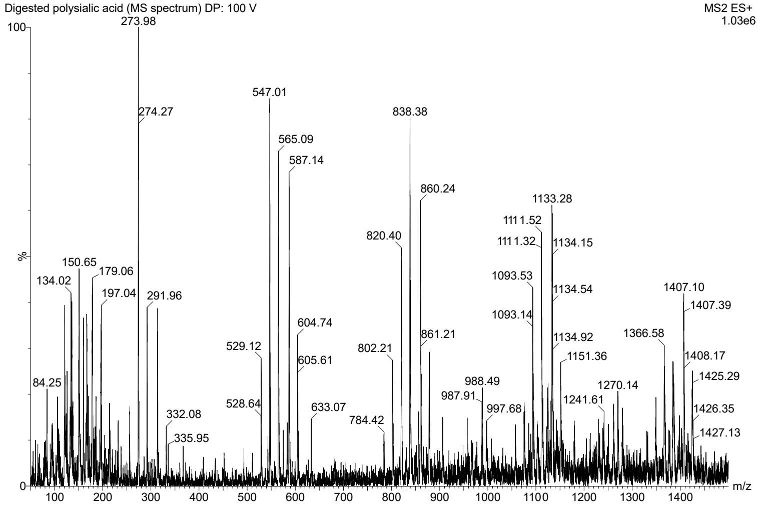	N/A
Digested polyethylene glycol polyglutamic acid	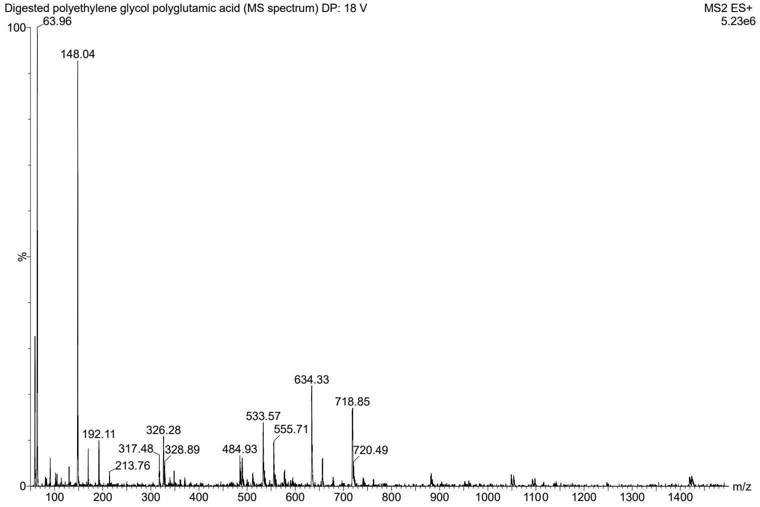	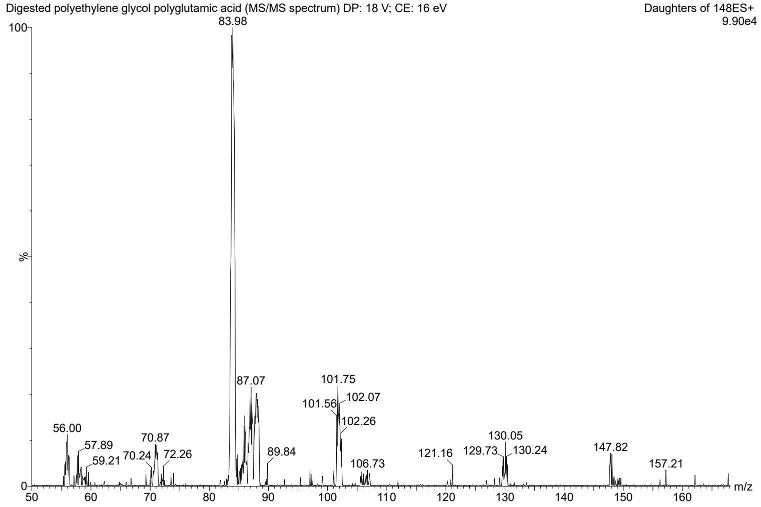

DP: declustering potential. CE: collision energy. N/A: not applicable. MS spectra were registered with the same DP as the one in the analytical method. MS/MS spectra were registered with the same DP and CE as the ones in the analytical method.

Supplementary Table 7. Mass spectrometry methodology and fragmentation pattern of the NCs' components

Substance	Method	Parent	Daughter	Ref.
DL-α-tocopherol	MRM, *m/z* 431.4 → 165.0, DP: 40 V, CE: 18 eV	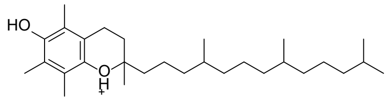	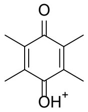	[1,2]
Benzethonium chloride	MRM, *m/z* 412.3 → 91.0, DP: 45 V, CE: 44 eV	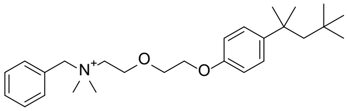	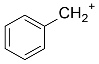	[3,4]
D-α-tocopherol polyethylene glycol 1000 succinate	MRM, *m/z* 557.4 → 99.0, DP: 50 V, CE: 30 eV	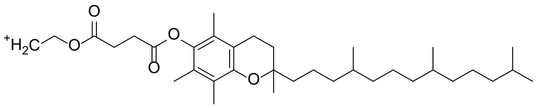	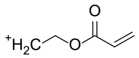	-
Hexadecyltrimethylammonium bromide	MRM, *m/z* 284.3 → 60.1, DP: 2 V, CE: 28 eV	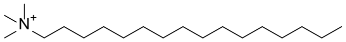	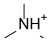	[5]
1,2-dioleoyl-3-trimethylammoniumpropane chloride	MRM, *m/z* 662.6 → 603.5, DP: 54 V, CE: 30 eV	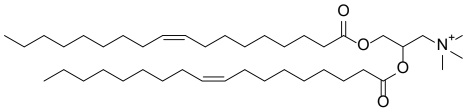	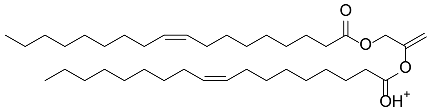	[6]
Miglyol® 812N	MRM, *m/z* 488.4 → 327.2, DP: 30 V, CE: 14 eV	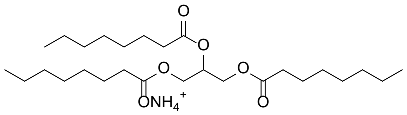	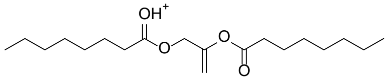	[7]
	MRM, *m/z* 516.4 → 355.3, DP: 32 V, CE: 16 eV	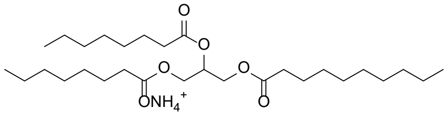	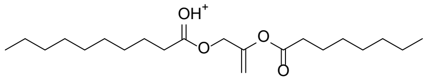	[7]
	MRM, *m/z* 544.5 → 355.3, DP: 34 V, CE: 18 eV	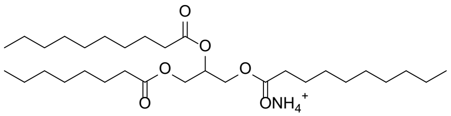	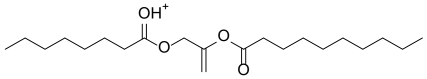	[7]
	MRM, *m/z* 572.5 → 383.3, DP: 36 V, CE: 18 eV	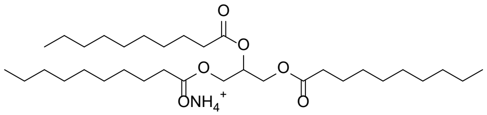	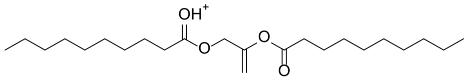	[7]

Supplementary Table 7. Mass spectrometry methodology and fragmentation pattern of the NCs' components (continuation)

Substance	Method	Parent	Daughter	Ref.
Phosphatidylcholines (Epikuron™ 145V)	MRM, *m/z* 756.6 → 184.1, DP: 50 V, CE: 30 eV	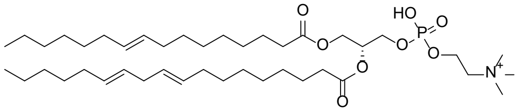 (*)	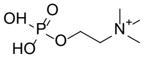	8
	MRM, *m/z* 758.6 → 184.1, DP: 50 V, CE: 30 eV	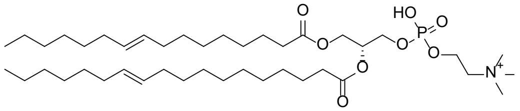 (*)		
	MRM, *m/z* 780.6 → 184.1, DP: 50 V, CE: 30 eV	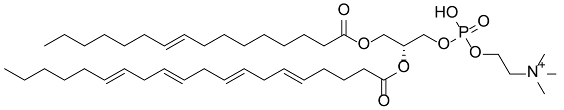 (*)		
	MRM, *m/z* 782.5 → 184.1, DP: 50 V, CE: 30 eV	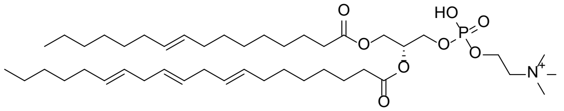 (*)		
Macrogol-15-hydroxystearate (Kolliphor® HS15)	PseudoMRM, *m/z* 309.3 → 309.3, DP: 90 V, CE: 5 eV	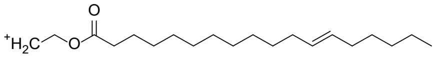	N/A	9
Polysorbate 80	MRM, *m/z* 804.6 → 309.3, DP: 30 V, CE: 40 eV	N/D	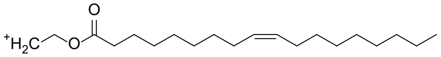	10
Digested hyaluronic acid	SIM, *m/z* 759.2, DP: 100 V	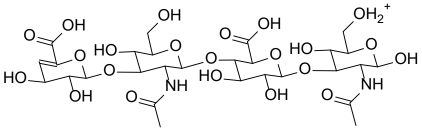	N/A	11–13
Digested polysialic acid	SIM, *m/z* 274.1, DP: 100 V	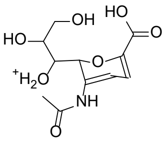	N/A	14,15
Digested polyethylene glycol poly-glutamic acid	MRM, *m/z* 148.1 → 84.1, DP: 18 V, CE: 16 eV	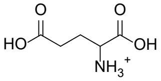	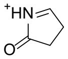 (*)	16

MRM: multiple reaction monitoring. SIM: Selected ion monitoring. DP: declustering potential. CE: collision energy. N/A: not applicable. N/D: not determined. (*): A possible structure; other molecules with the same m/z may co-exist.

Supplementary Table 8. Calibration curves and residual plots of all the quantified substances

Formulation	Molecule	Rep.	Residual plots and calibration curves
SD-*l*-PEG-HA	Benzethonium	1	Compound name: benzethonium<br>Correlation coefficient: r = 0.998880, r^2 = 0.997761<br>Calibration curve: 20062.6 * x + 84251.2<br>Response type: External Std, Area<br>Curve type: Linear, Origin: Exclude, Weighting: 1/x, Axis trans: None<br>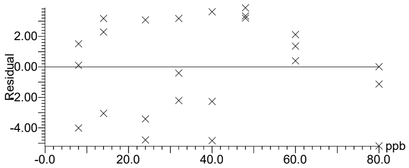 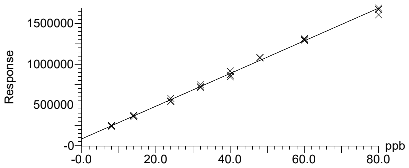
		2	Compound name: benzethonium<br>Correlation coefficient: r = 0.997675, r^2 = 0.995356<br>Calibration curve: 20667.3 * x + 109777<br>Response type: External Std, Area<br>Curve type: Linear, Origin: Exclude, Weighting: 1/x, Axis trans: None<br>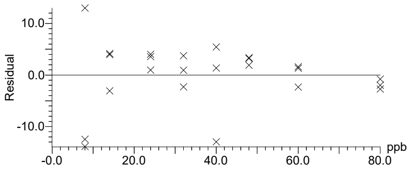 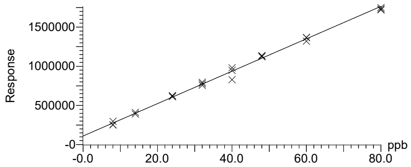
		3	Compound name: benzethonium<br>Correlation coefficient: r = 0.997362, r^2 = 0.994731<br>Calibration curve: 18278.7 * x + 118770<br>Response type: External Std, Area<br>Curve type: Linear, Origin: Exclude, Weighting: 1/x, Axis trans: None<br>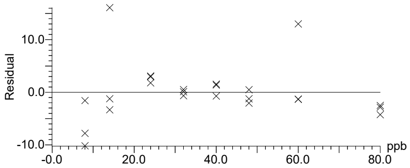 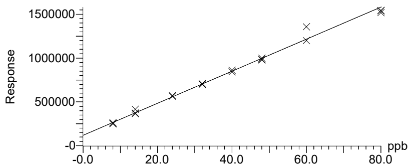
	D-a-tocopherol polyethylene glycol 1000 succinate	1	Compound name: D-α-tocopherol polyethylene glycol 1000 succinate<br>Correlation coefficient: r = 0.990478, r^2 = 0.981047<br>Calibration curve: 181.548 * x + 1133.68<br>Response type: External Std, Area<br>Curve type: Linear, Origin: Exclude, Weighting: 1/x, Axis trans: None<br>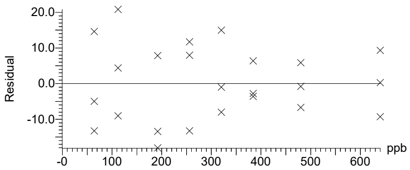 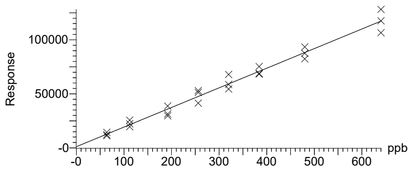
		2	Compound name: D-α-tocopherol polyethylene glycol 1000 succinate<br>Correlation coefficient: r = 0.993116, r^2 = 0.986279<br>Calibration curve: 190.369 * x + -843.895<br>Response type: External Std, Area<br>Curve type: Linear, Origin: Exclude, Weighting: 1/x, Axis trans: None<br>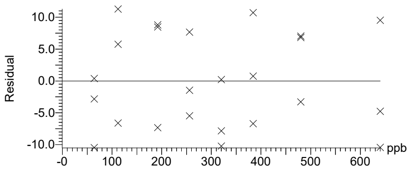 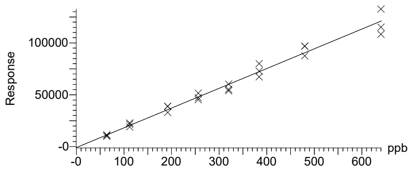
		3	Compound name: D-α-tocopherol polyethylene glycol 1000 succinate<br>Correlation coefficient: r = 0.994383, r^2 = 0.988797<br>Calibration curve: 398.723 * x + -2160.03<br>Response type: External Std, Area<br>Curve type: Linear, Origin: Exclude, Weighting: 1/x, Axis trans: None<br>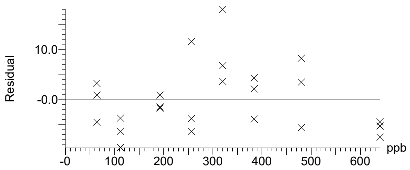 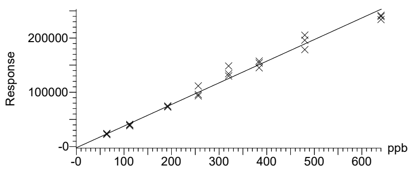

Supplementary Table 8. Calibration curves and residual plots of all the quantified substances (continuation)

Formulation	Molecule	Rep.	Residual plots and calibration curves
SD-*l*-PEG-HA (continuation)	D-α-tocopherol	1	Compound name: DL-α-tocopherol<br>Correlation coefficient: r = 0.996368, r^2 = 0.992749<br>Calibration curve: 161.6 * x + -621.845<br>Response type: External Std, Area<br>Curve type: Linear, Origin: Exclude, Weighting: 1/x, Axis trans: None<br>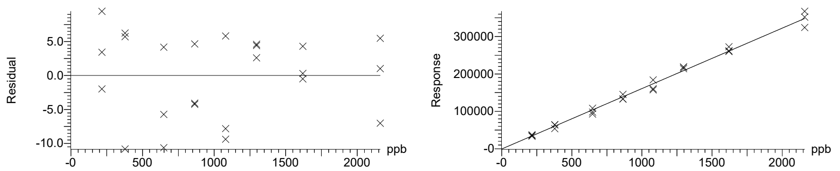
		2	Compound name: DL-α-tocopherol<br>Correlation coefficient: r = 0.996091, r^2 = 0.992198<br>Calibration curve: 134.842 * x + 259.387<br>Response type: External Std, Area<br>Curve type: Linear, Origin: Exclude, Weighting: 1/x, Axis trans: None<br>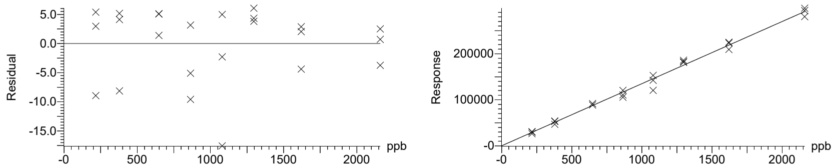
		3	Compound name: DL-α-tocopherol<br>Correlation coefficient: r = 0.999656, r^2 = 0.999312<br>Calibration curve: 126.766 * x + -621.553<br>Response type: External Std, Area<br>Curve type: Linear, Origin: Exclude, Weighting: 1/x, Axis trans: None<br>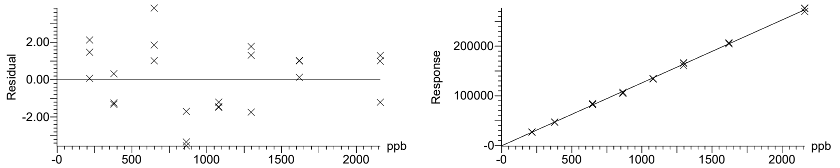
	Hyaluronic acid (whole)	1	Compound name: Digested hyaluronic acid<br>Correlation coefficient: r = 0.994386, r^2 = 0.988803<br>Calibration curve: 4130.13 * x + 9837.9<br>Response type: External Std, Area<br>Curve type: Linear, Origin: Exclude, Weighting: 1/x, Axis trans: None<br>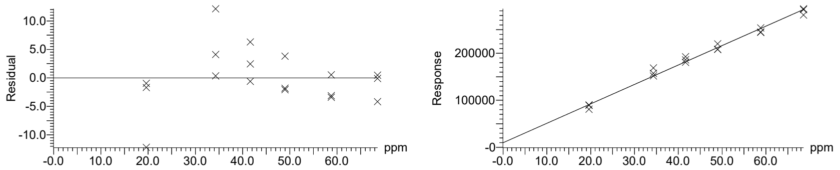
		2	Compound name: Digested hyaluronic acid<br>Correlation coefficient: r = 0.995225, r^2 = 0.990474<br>Calibration curve: 4373.25 * x + -7479.95<br>Response type: External Std, Area<br>Curve type: Linear, Origin: Exclude, Weighting: 1/x, Axis trans: None<br>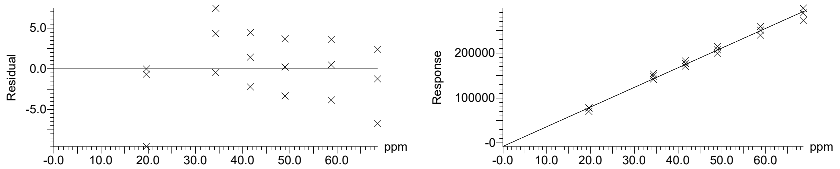
		3	Compound name: Digested hyaluronic acid<br>Correlation coefficient: r = 0.994960, r^2 = 0.989945<br>Calibration curve: 4461.42 * x + 1376.84<br>Response type: External Std, Area<br>Curve type: Linear, Origin: Exclude, Weighting: 1/x, Axis trans: None<br>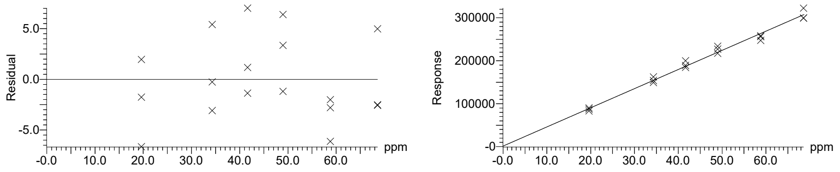

Supplementary Table 8. Calibration curves and residual plots of all the quantified substances (continuation)

Formulation	Molecule	Rep.	Residual plots and calibration curves
SD-*l*-PEG-HA (continuation)	Hyaluronic acid (supernatant)	1	Compound name: Digested hyaluronic acid<br>Correlation coefficient: r = 0.992396, r^2 = 0.984849<br>Calibration curve: 4199.58 * x + 6712.82<br>Response type: External Std, Area<br>Curve type: Linear, Origin: Exclude, Weighting: 1/x, Axis trans: None<br>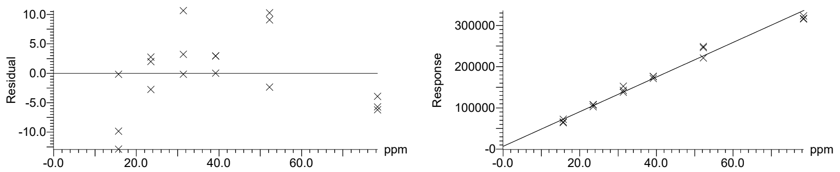
		2	Compound name: Digested hyaluronic acid<br>Correlation coefficient: r = 0.994621, r^2 = 0.989270<br>Calibration curve: 4207 * x + -11030.1<br>Response type: External Std, Area<br>Curve type: Linear, Origin: Exclude, Weighting: 1/x, Axis trans: None<br>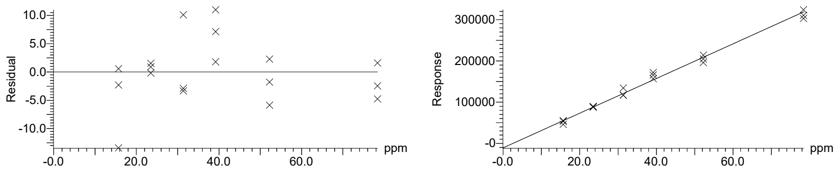
		3	Compound name: Digested hyaluronic acid<br>Correlation coefficient: r = 0.996289, r^2 = 0.992591<br>Calibration curve: 4373.41 * x + -583.121<br>Response type: External Std, Area<br>Curve type: Linear, Origin: Exclude, Weighting: 1/x, Axis trans: None<br>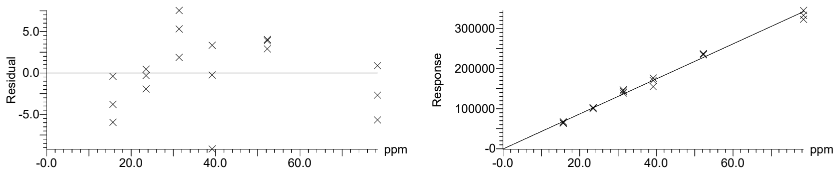
	Hyaluronic acid (infranatant)	1	Compound name: Digested hyaluronic acid<br>Correlation coefficient: r = 0.995210, r^2 = 0.990444<br>Calibration curve: 4956.23 * x + 2094.75<br>Response type: External Std, Area<br>Curve type: Linear, Origin: Exclude, Weighting: 1/x, Axis trans: None<br>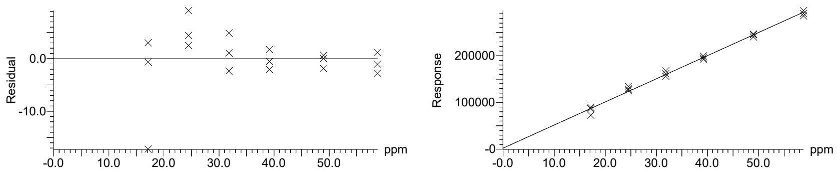
		2	Compound name: Digested hyaluronic acid<br>Correlation coefficient: r = 0.990110, r^2 = 0.980318<br>Calibration curve: 4668.12 * x + 1446.87<br>Response type: External Std, Area<br>Curve type: Linear, Origin: Exclude, Weighting: 1/x, Axis trans: None<br>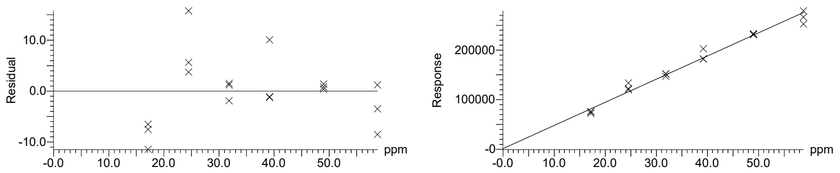
		3	Compound name: Digested hyaluronic acid<br>Correlation coefficient: r = 0.990600, r^2 = 0.981288<br>Calibration curve: 4955.03 * x + -8467.49<br>Response type: External Std, Area<br>Curve type: Linear, Origin: Exclude, Weighting: 1/x, Axis trans: None<br>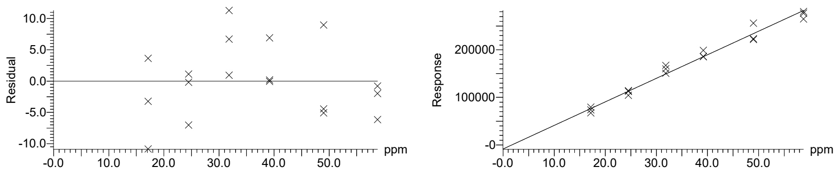

Supplementary Table 8. Calibration curves and residual plots of all the quantified substances (continuation)

Formulation	Molecule	Rep.	Residual plots and calibration curves
SD-noPEG-HA	CTAB	1	Compound name: hexadecyltrimethylammonium<br>Correlation coefficient: r = 0.998943, r^2 = 0.997887<br>Calibration curve: 4126.68 * x + 13983.3<br>Response type: External Std, Area<br>Curve type: Linear, Origin: Exclude, Weighting: 1/x, Axis trans: None<br>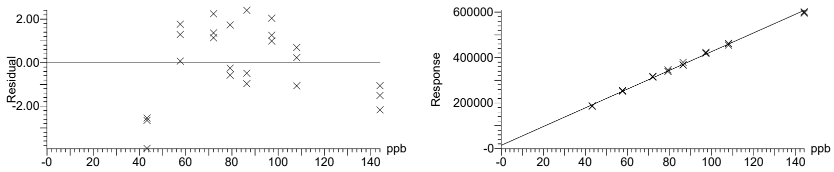
		2	Compound name: hexadecyltrimethylammonium<br>Correlation coefficient: r = 0.999078, r^2 = 0.998157<br>Calibration curve: 4266.63 * x + 22962.8<br>Response type: External Std, Area<br>Curve type: Linear, Origin: Exclude, Weighting: 1/x, Axis trans: None<br>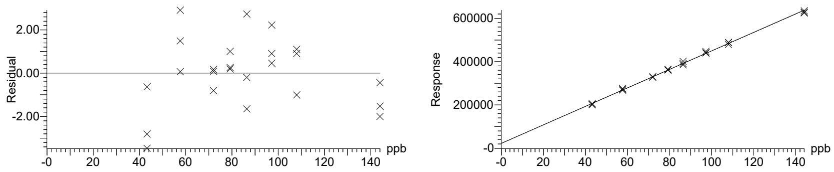
		3	Compound name: hexadecyltrimethylammonium<br>Correlation coefficient: r = 0.999769, r^2 = 0.999538<br>Calibration curve: 4265.8 * x + 15844.5<br>Response type: External Std, Area<br>Curve type: Linear, Origin: Exclude, Weighting: 1/x, Axis trans: None<br>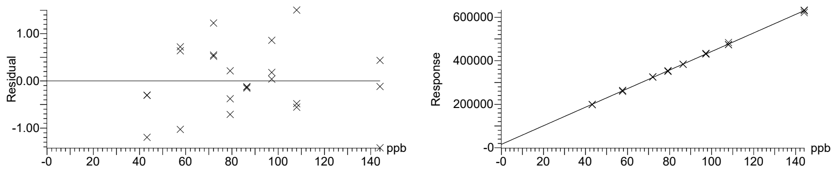
	C_8-C_8-C_8 Triglyceride	1	Compound name: C8-C8-C8 TG<br>Correlation coefficient: r = 0.994379, r^2 = 0.988791<br>Calibration curve: 2109.29 * x + 1.23567e+006<br>Response type: External Std, Area<br>Curve type: Linear, Origin: Exclude, Weighting: 1/x, Axis trans: None<br>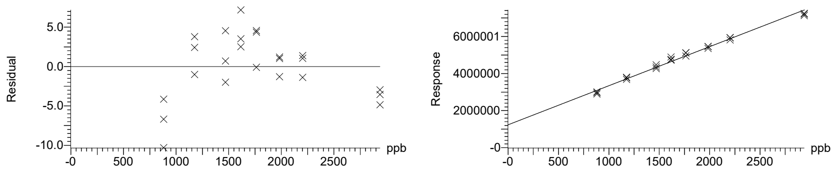
		2	Compound name: C8-C8-C8 TG<br>Correlation coefficient: r = 0.994465, r^2 = 0.988961<br>Calibration curve: 1111.31 * x + 527248<br>Response type: External Std, Area<br>Curve type: Linear, Origin: Exclude, Weighting: 1/x, Axis trans: None<br>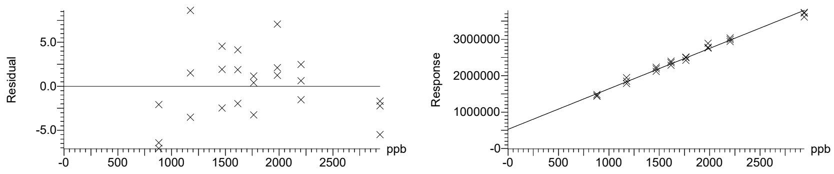
		3	Compound name: C8-C8-C8 TG<br>Correlation coefficient: r = 0.993040, r^2 = 0.986129<br>Calibration curve: 1850.95 * x + 977854<br>Response type: External Std, Area<br>Curve type: Linear, Origin: Exclude, Weighting: 1/x, Axis trans: None<br>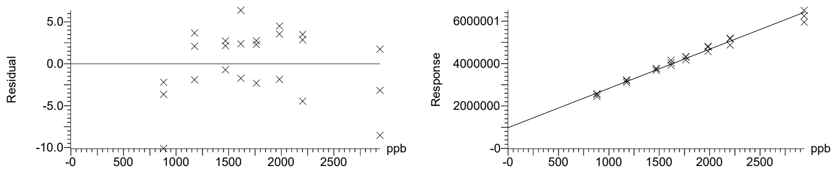

Supplementary Table 8. Calibration curves and residual plots of all the quantified substances (continuation)

Formulation	Molecule	Rep.	Residual plots and calibration curves
SD-noPEG-HA (continuation)	C_8-C_8-C_{10} Triglyceride	1	Compound name: C8-C8-C10 TG<br>Correlation coefficient: r = 0.991232, r^2 = 0.982542<br>Calibration curve: 2391.65 * x + 1.51379e+006<br>Response type: External Std, Area<br>Curve type: Linear, Origin: Exclude, Weighting: 1/x, Axis trans: None<br>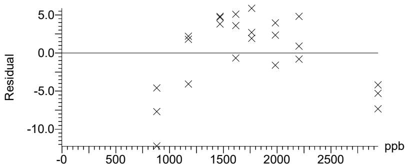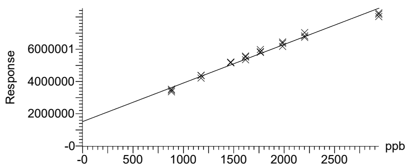
		2	Compound name: C8-C8-C10 TG<br>Correlation coefficient: r = 0.995155, r^2 = 0.990334<br>Calibration curve: 1508.44 * x + 679958<br>Response type: External Std, Area<br>Curve type: Linear, Origin: Exclude, Weighting: 1/x, Axis trans: None<br>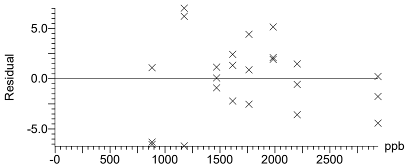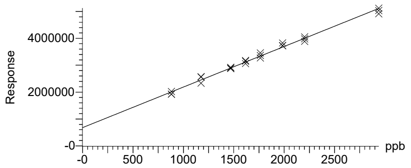
		3	Compound name: C8-C8-C10 TG<br>Correlation coefficient: r = 0.992451, r^2 = 0.984958<br>Calibration curve: 2200.96 * x + 1.22044e+006<br>Response type: External Std, Area<br>Curve type: Linear, Origin: Exclude, Weighting: 1/x, Axis trans: None<br>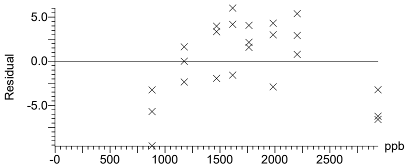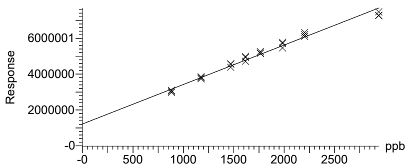
	C_8-C_{10}-C_{10} Triglyceride	1	Compound name: C8-C10-C10 TG<br>Correlation coefficient: r = 0.993350, r^2 = 0.986744<br>Calibration curve: 1618.69 * x + 866952<br>Response type: External Std, Area<br>Curve type: Linear, Origin: Exclude, Weighting: 1/x, Axis trans: None<br>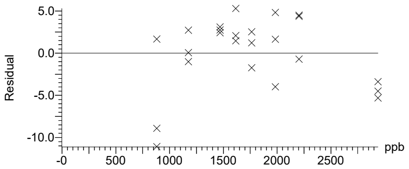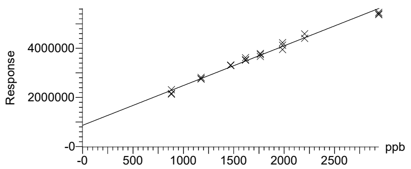
		2	Compound name: C8-C10-C10 TG<br>Correlation coefficient: r = 0.993628, r^2 = 0.987296<br>Calibration curve: 1101.43 * x + 479398<br>Response type: External Std, Area<br>Curve type: Linear, Origin: Exclude, Weighting: 1/x, Axis trans: None<br>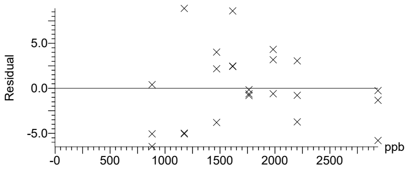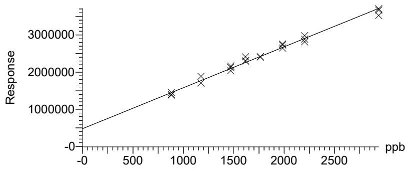
		3	Compound name: C8-C10-C10 TG<br>Correlation coefficient: r = 0.990811, r^2 = 0.981706<br>Calibration curve: 1562.36 * x + 603095<br>Response type: External Std, Area<br>Curve type: Linear, Origin: Exclude, Weighting: 1/x, Axis trans: None<br>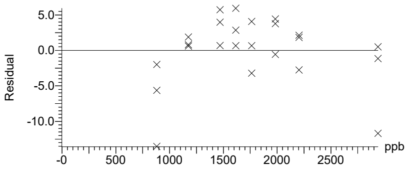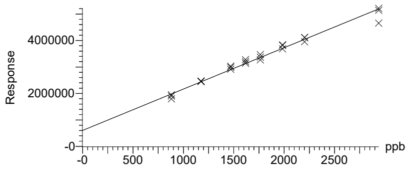

Supplementary Table 8. Calibration curves and residual plots of all the quantified substances (continuation)

Formulation	Molecule	Rep.	Residual plots and calibration curves
SD-noPEG-HA (continuation)	C_{10}-C_{10}-C_{10} Triglyceride	1	Compound name: C10-C10-C10 TG<br>Correlation coefficient: r = 0.991252, r^2 = 0.982581<br>Calibration curve: 520.173 * x + 215015<br>Response type: External Std, Area<br>Curve type: Linear, Origin: Exclude, Weighting: 1/x, Axis trans: None<br>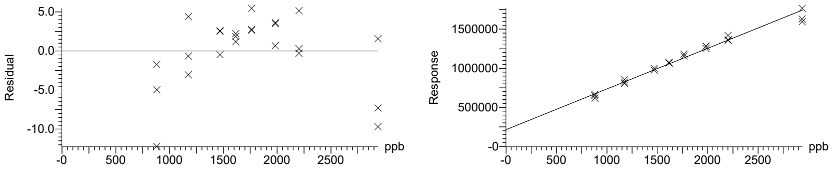
		2	Compound name: C10-C10-C10 TG<br>Correlation coefficient: r = 0.989802, r^2 = 0.979709<br>Calibration curve: 374.753 * x + 106561<br>Response type: External Std, Area<br>Curve type: Linear, Origin: Exclude, Weighting: 1/x, Axis trans: None<br>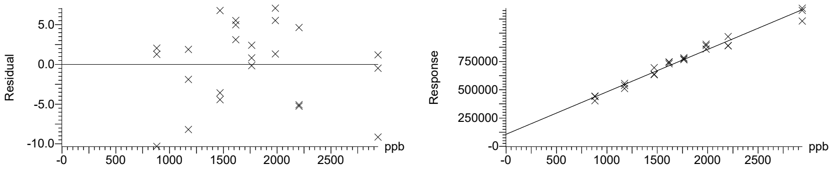
		3	Compound name: C10-C10-C10 TG<br>Correlation coefficient: r = 0.987630, r^2 = 0.975413<br>Calibration curve: 498.949 * x + 127026<br>Response type: External Std, Area<br>Curve type: Linear, Origin: Exclude, Weighting: 1/x, Axis trans: None<br>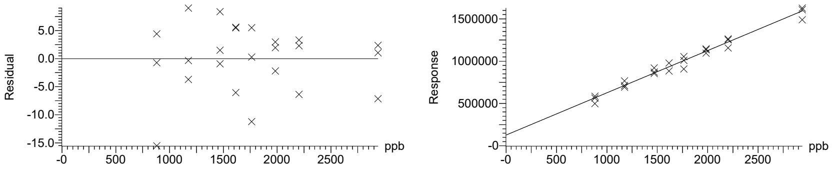
	Phosphatidylcholine 756.6 Da	1	Compound name: PC_756.5<br>Correlation coefficient: r = 0.997083, r^2 = 0.994175<br>Calibration curve: 62.1519 * x + -6144.89<br>Response type: External Std, Area<br>Curve type: Linear, Origin: Exclude, Weighting: 1/x, Axis trans: None<br>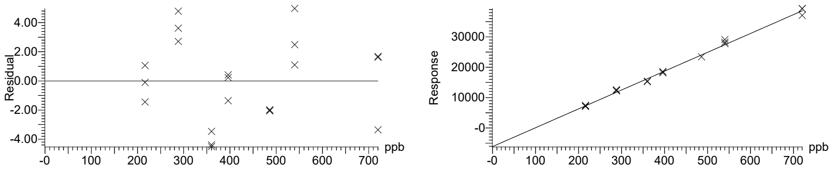
		2	Compound name: PC_756.5<br>Correlation coefficient: r = 0.996890, r^2 = 0.993789<br>Calibration curve: 403.916 * x + -57356.6<br>Response type: External Std, Area<br>Curve type: Linear, Origin: Exclude, Weighting: 1/x, Axis trans: None<br>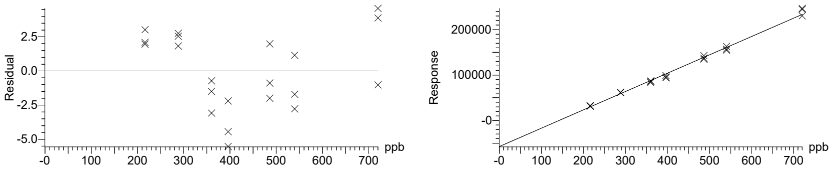
		3	Compound name: PC_756.5<br>Correlation coefficient: r = 0.992193, r^2 = 0.984448<br>Calibration curve: 73.7867 * x + -9540.06<br>Response type: External Std, Area<br>Curve type: Linear, Origin: Exclude, Weighting: 1/x, Axis trans: None<br>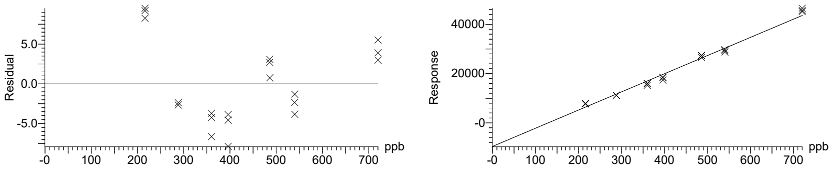

Supplementary Table 8. Calibration curves and residual plots of all the quantified substances (continuation)

Formulation	Molecule	Rep.	Residual plots and calibration curves
SD-noPEG-HA (continuation)	Phosphatidylcholine 758.6 Da	1	Compound name: PC_758.5<br>Correlation coefficient: r = 0.981541, r^2 = 0.963422<br>Calibration curve: 465.7 * x + -47279.4<br>Response type: External Std, Area<br>Curve type: Linear, Origin: Exclude, Weighting: 1/x, Axis trans: None<br>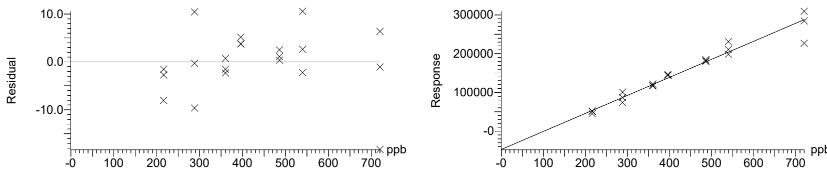
		2	Compound name: PC_758.5<br>Correlation coefficient: r = 0.996625, r^2 = 0.993262<br>Calibration curve: 427.877 * x + -61715.9<br>Response type: External Std, Area<br>Curve type: Linear, Origin: Exclude, Weighting: 1/x, Axis trans: None<br>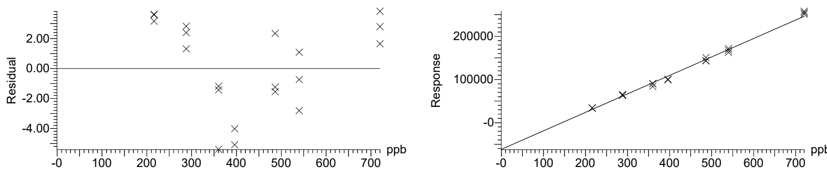
		3	Compound name: PC_758.5<br>Correlation coefficient: r = 0.989484, r^2 = 0.979078<br>Calibration curve: 579.431 * x + -77046.3<br>Response type: External Std, Area<br>Curve type: Linear, Origin: Exclude, Weighting: 1/x, Axis trans: None<br>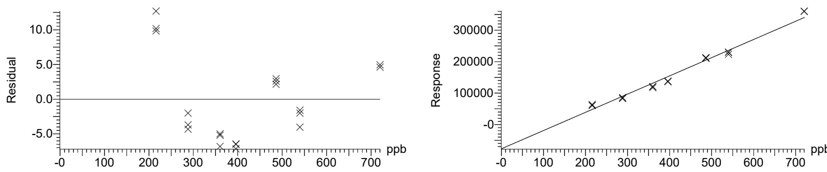
	Phosphatidylcholine 780.6 Da	1	Compound name: PC_780.5<br>Correlation coefficient: r = 0.996934, r^2 = 0.993877<br>Calibration curve: 249.502 * x + -22763.5<br>Response type: External Std, Area<br>Curve type: Linear, Origin: Exclude, Weighting: 1/x, Axis trans: None<br>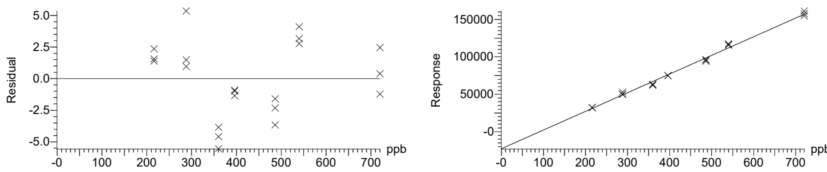
		2	Compound name: PC_780.5<br>Correlation coefficient: r = 0.998443, r^2 = 0.996889<br>Calibration curve: 1010.34 * x + -133621<br>Response type: External Std, Area<br>Curve type: Linear, Origin: Exclude, Weighting: 1/x, Axis trans: None<br>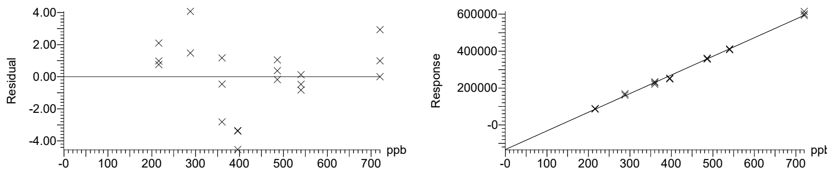
		3	Compound name: PC_780.5<br>Correlation coefficient: r = 0.993020, r^2 = 0.986089<br>Calibration curve: 279.912 * x + -34205.1<br>Response type: External Std, Area<br>Curve type: Linear, Origin: Exclude, Weighting: 1/x, Axis trans: None<br>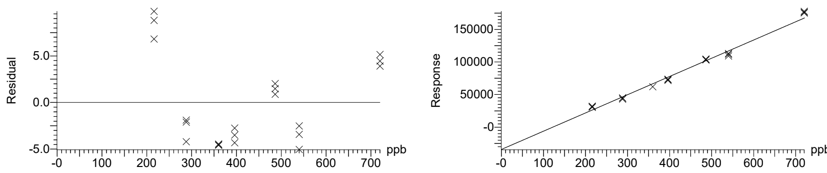

Supplementary Table 8. Calibration curves and residual plots of all the quantified substances (continuation)

Formulation	Molecule	Rep.	Residual plots and calibration curves
SD-noPEG-HA (continuation)	Phosphatidylcholine 782.5 Da	1	Compound name: PC_782.5<br>Correlation coefficient: r = 0.997218, r^2 = 0.994443<br>Calibration curve: 1226.51 * x + -122120<br>Response type: External Std, Area<br>Curve type: Linear, Origin: Exclude, Weighting: 1/x, Axis trans: None<br>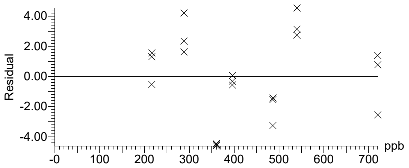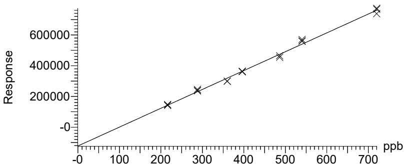
		2	Compound name: PC_782.5<br>Correlation coefficient: r = 0.997761, r^2 = 0.995526<br>Calibration curve: 1131.64 * x + -154034<br>Response type: External Std, Area<br>Curve type: Linear, Origin: Exclude, Weighting: 1/x, Axis trans: None<br>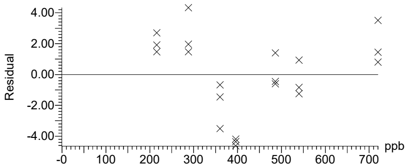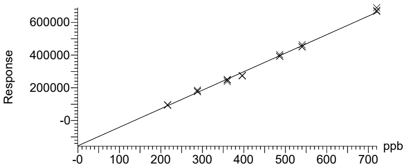
		3	Compound name: PC_782.5<br>Correlation coefficient: r = 0.990150, r^2 = 0.980398<br>Calibration curve: 1362.81 * x + -179537<br>Response type: External Std, Area<br>Curve type: Linear, Origin: Exclude, Weighting: 1/x, Axis trans: None<br>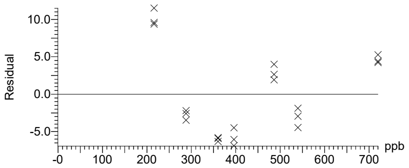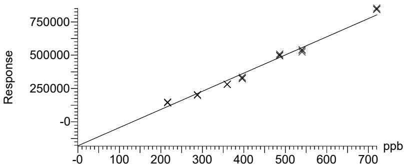
	Hyaluronic acid (whole)	1	Compound name: Digested hyaluronic acid<br>Correlation coefficient: r = 0.986828, r^2 = 0.973829<br>Calibration curve: 6523.57 * x + -15392.9<br>Response type: External Std, Area<br>Curve type: Linear, Origin: Exclude, Weighting: 1/x, Axis trans: None<br>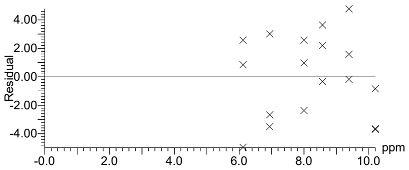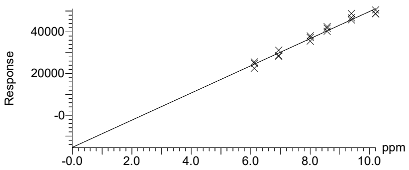
		2	Compound name: Digested hyaluronic acid<br>Correlation coefficient: r = 0.967398, r^2 = 0.935858<br>Calibration curve: 4864.8 * x + 1886.82<br>Response type: External Std, Area<br>Curve type: Linear, Origin: Exclude, Weighting: 1/x, Axis trans: None<br>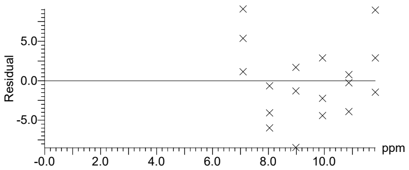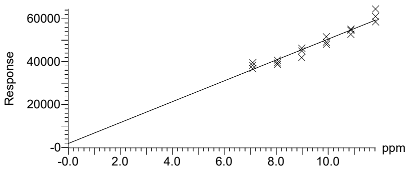
		3	Compound name: Digested hyaluronic acid<br>Correlation coefficient: r = 0.988969, r^2 = 0.978060<br>Calibration curve: 3370.43 * x + -22997<br>Response type: External Std, Area<br>Curve type: Linear, Origin: Exclude, Weighting: 1/x, Axis trans: None<br>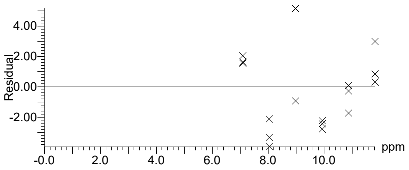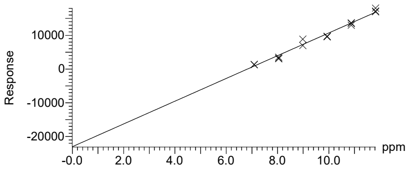

Supplementary Table 8. Calibration curves and residual plots of all the quantified substances (continuation)

<table>
<tr><th>Formulation</th><th>Molecule</th><th>Rep.</th><th>Residual plots and calibration curves</th></tr>
<tr><td>SD-noPEG-HA (continuation)</td><td>Hyaluronic acid (supernatant)</td><td>1</td><td>Compound name: Digested hyaluronic acid
Correlation coefficient: r = 0.992565, r^2 = 0.985185
Calibration curve: 4404.76 * x + -14916.4
Response type: External Std, Area
Curve type: Linear, Origin: Exclude, Weighting: 1/x, Axis trans: None
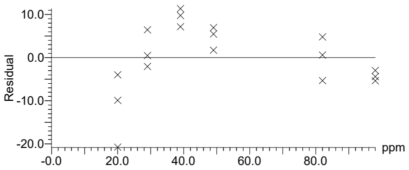

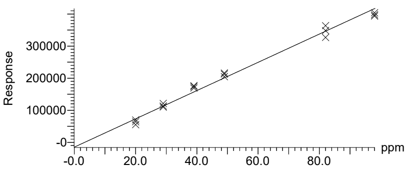
</td></tr>
<tr><td></td><td></td><td>2</td><td>Compound name: Digested hyaluronic acid
Correlation coefficient: r = 0.996035, r^2 = 0.992085
Calibration curve: 4135.47 * x + -31361.2
Response type: External Std, Area
Curve type: Linear, Origin: Exclude, Weighting: 1/x, Axis trans: None
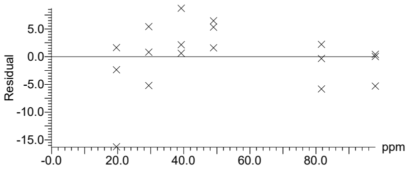

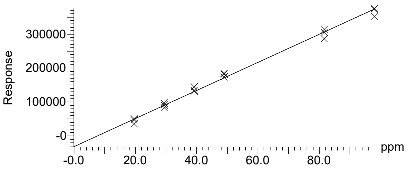
</td></tr>
<tr><td></td><td></td><td>3</td><td>Compound name: Digested hyaluronic acid
Correlation coefficient: r = 0.993772, r^2 = 0.987583
Calibration curve: 3391.73 * x + -39008.5
Response type: External Std, Area
Curve type: Linear, Origin: Exclude, Weighting: 1/x, Axis trans: None
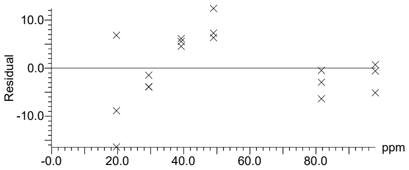

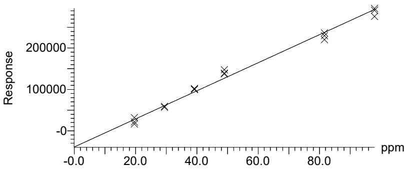
</td></tr>
<tr><td></td><td>Hyaluronic acid (infranatant)</td><td>1</td><td>Compound name: Digested hyaluronic acid
Correlation coefficient: r = 0.993810, r^2 = 0.987659
Calibration curve: 5893.24 * x + 1224.65
Response type: External Std, Area
Curve type: Linear, Origin: Exclude, Weighting: 1/x, Axis trans: None
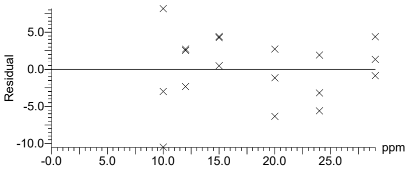

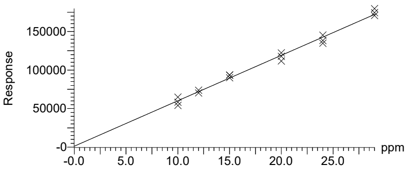
</td></tr>
<tr><td></td><td></td><td>2</td><td>Compound name: Digested hyaluronic acid
Correlation coefficient: r = 0.994331, r^2 = 0.988695
Calibration curve: 4370.09 * x + -9223.01
Response type: External Std, Area
Curve type: Linear, Origin: Exclude, Weighting: 1/x, Axis trans: None
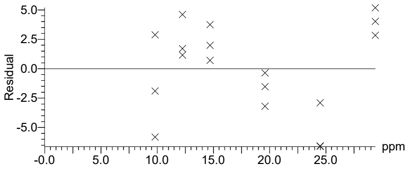

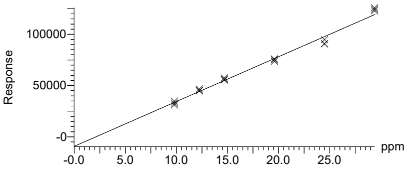
</td></tr>
<tr><td></td><td></td><td>3</td><td>Compound name: Digested hyaluronic acid
Correlation coefficient: r = 0.991640, r^2 = 0.983349
Calibration curve: 4115.71 * x + -21346.5
Response type: External Std, Area
Curve type: Linear, Origin: Exclude, Weighting: 1/x, Axis trans: None
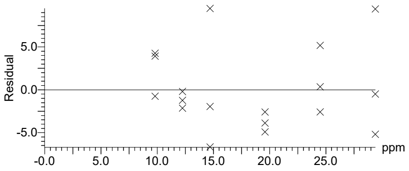

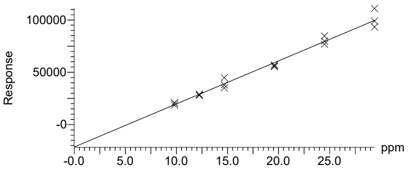
</td></tr>
</table>

Supplementary Table 8. Calibration curves and residual plots of all the quantified substances (continuation)

Formulation	Molecule	Rep.	Residual plots and calibration curves
SD-noPEG-PSA	CTAB	1	Compound name: hexadecyltrimethylammonium<br>Correlation coefficient: r = 0.997990, r^2 = 0.995984<br>Calibration curve: 3657.04 * x + 2984.29<br>Response type: External Std, Area<br>Curve type: Linear, Origin: Exclude, Weighting: 1/x, Axis trans: None<br>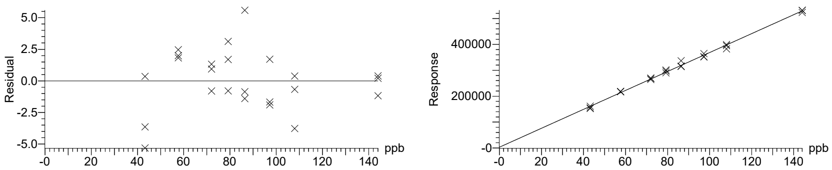
		2	Compound name: hexadecyltrimethylammonium<br>Correlation coefficient: r = 0.998108, r^2 = 0.996219<br>Calibration curve: 3114.3 * x + 39261.4<br>Response type: External Std, Area<br>Curve type: Linear, Origin: Exclude, Weighting: 1/x, Axis trans: None<br>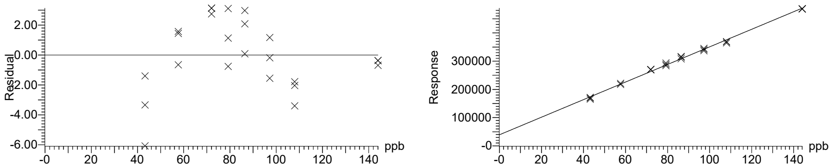
		3	Compound name: hexadecyltrimethylammonium<br>Correlation coefficient: r = 0.996723, r^2 = 0.993458<br>Calibration curve: 3447.94 * x + 20029.8<br>Response type: External Std, Area<br>Curve type: Linear, Origin: Exclude, Weighting: 1/x, Axis trans: None<br>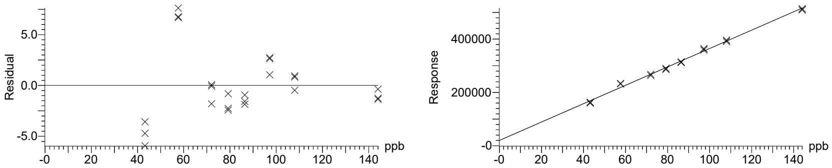
	C_8-C_8-C_8 Triglyceride	1	Compound name: C8-C8-C8 TG<br>Correlation coefficient: r = 0.989550, r^2 = 0.979210<br>Calibration curve: 1951.37 * x + 1.00607e+006<br>Response type: External Std, Area<br>Curve type: Linear, Origin: Exclude, Weighting: 1/x, Axis trans: None<br>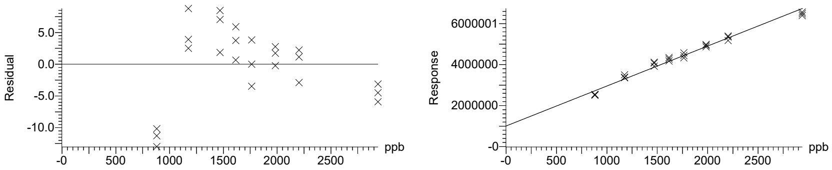
		2	Compound name: C8-C8-C8 TG<br>Correlation coefficient: r = 0.990411, r^2 = 0.980914<br>Calibration curve: 1452.6 * x + 1.77029e+006<br>Response type: External Std, Area<br>Curve type: Linear, Origin: Exclude, Weighting: 1/x, Axis trans: None<br>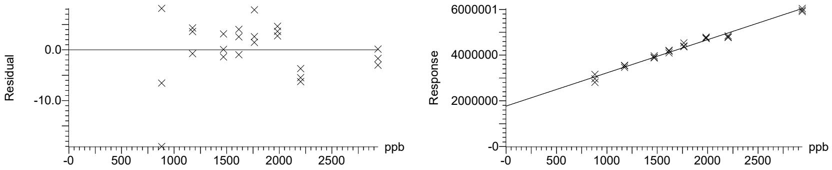
		3	Compound name: C8-C8-C8 TG<br>Correlation coefficient: r = 0.992065, r^2 = 0.984193<br>Calibration curve: 1676.78 * x + 1.24921e+006<br>Response type: External Std, Area<br>Curve type: Linear, Origin: Exclude, Weighting: 1/x, Axis trans: None<br>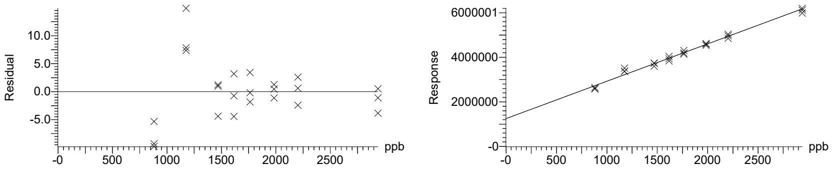

Supplementary Table 8. Calibration curves and residual plots of all the quantified substances (continuation)

Formulation	Molecule		Rep.	Residual plots and calibration curves
SD-noPEG-PSA (continuation)	C_8-C_8-C_{10} glyceride	Tri-	1	Compound name: C8-C8-C10 TG<br>Correlation coefficient: r = 0.989462, r^2 = 0.979035<br>Calibration curve: 2163.78 * x + 1.20952e+006<br>Response type: External Std, Area<br>Curve type: Linear, Origin: Exclude, Weighting: 1/x, Axis trans: None<br>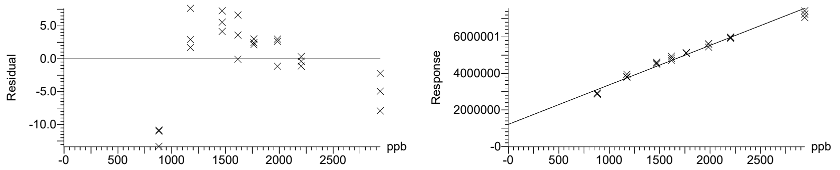
			2	Compound name: C8-C8-C10 TG<br>Correlation coefficient: r = 0.990996, r^2 = 0.982073<br>Calibration curve: 1569.23 * x + 1.92218e+006<br>Response type: External Std, Area<br>Curve type: Linear, Origin: Exclude, Weighting: 1/x, Axis trans: None<br>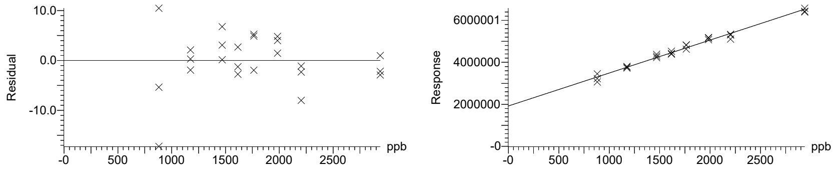
			3	Compound name: C8-C8-C10 TG<br>Correlation coefficient: r = 0.989581, r^2 = 0.979271<br>Calibration curve: 1854.36 * x + 1.51298e+006<br>Response type: External Std, Area<br>Curve type: Linear, Origin: Exclude, Weighting: 1/x, Axis trans: None<br>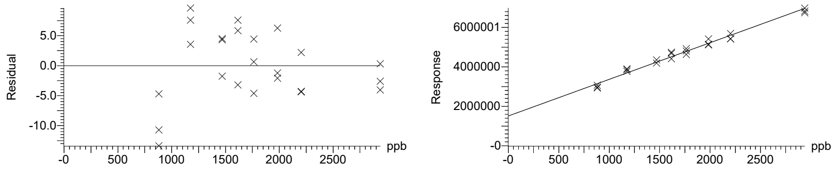
	C_8-C_{10}-C_{10} glyceride	Tri-	1	Compound name: C8-C10-C10 TG<br>Correlation coefficient: r = 0.992471, r^2 = 0.984999<br>Calibration curve: 1534.99 * x + 593742<br>Response type: External Std, Area<br>Curve type: Linear, Origin: Exclude, Weighting: 1/x, Axis trans: None<br>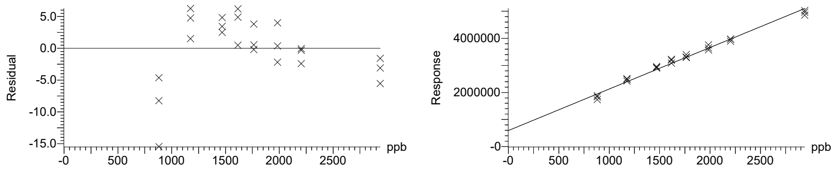
			2	Compound name: C8-C10-C10 TG<br>Correlation coefficient: r = 0.991897, r^2 = 0.983860<br>Calibration curve: 1112.18 * x + 1.04842e+006<br>Response type: External Std, Area<br>Curve type: Linear, Origin: Exclude, Weighting: 1/x, Axis trans: None<br>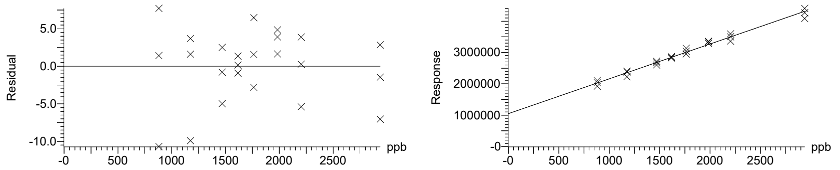
			3	Compound name: C8-C10-C10 TG<br>Correlation coefficient: r = 0.992729, r^2 = 0.985511<br>Calibration curve: 1274.23 * x + 866565<br>Response type: External Std, Area<br>Curve type: Linear, Origin: Exclude, Weighting: 1/x, Axis trans: None<br>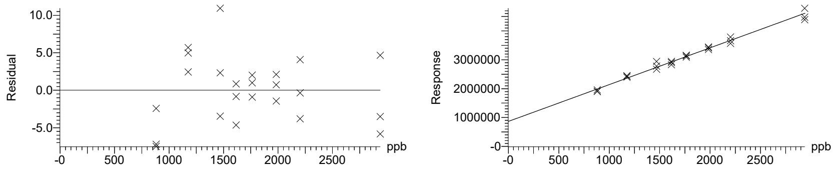

SUPPORTING INFORMATION | Quantification of the actual composition of polymeric nanocapsules. A quality control analysis

Supplementary Table 8. Calibration curves and residual plots of all the quantified substances (continuation)

Formulation	Molecule	Rep.	Residual plots and calibration curves
SD-noPEG-PSA (continuation)	C_{10}-C_{10}-C_{10} Triglyceride	1	Compound name: C10-C10-C10 TG<br>Correlation coefficient: r = 0.978117, r^2 = 0.956712<br>Calibration curve: 626.111 * x + -34775.9<br>Response type: External Std, Area<br>Curve type: Linear, Origin: Exclude, Weighting: 1/x, Axis trans: None<br>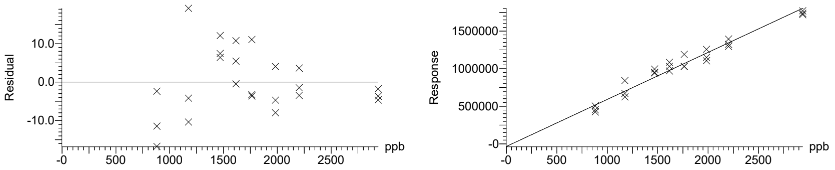
		2	Compound name: C10-C10-C10 TG<br>Correlation coefficient: r = 0.981912, r^2 = 0.964152<br>Calibration curve: 448.743 * x + 192818<br>Response type: External Std, Area<br>Curve type: Linear, Origin: Exclude, Weighting: 1/x, Axis trans: None<br>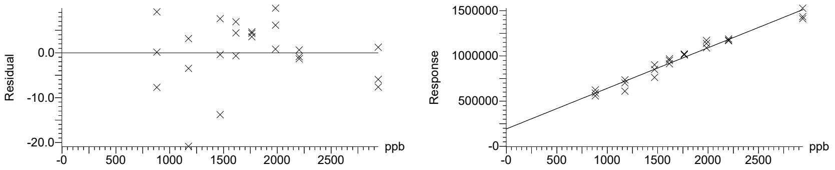
		3	Compound name: C10-C10-C10 TG<br>Correlation coefficient: r = 0.994138, r^2 = 0.988311<br>Calibration curve: 480.788 * x + 180271<br>Response type: External Std, Area<br>Curve type: Linear, Origin: Exclude, Weighting: 1/x, Axis trans: None<br>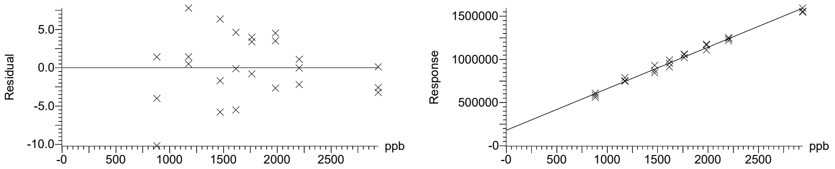
	Phosphatidylcholine 756.6 Da	1	Compound name: PC_756.5<br>Correlation coefficient: r = 0.981717, r^2 = 0.963769<br>Calibration curve: 47.4852 * x + -7090.4<br>Response type: External Std, Area<br>Curve type: Linear, Origin: Exclude, Weighting: 1/x, Axis trans: None<br>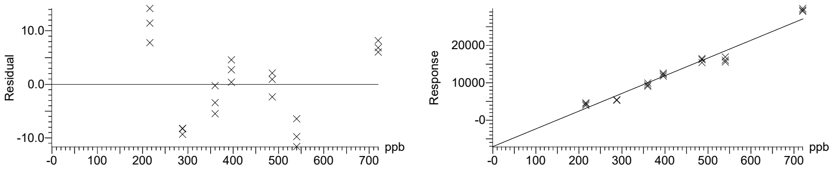
		2	Compound name: PC_756.5<br>Correlation coefficient: r = 0.971896, r^2 = 0.944581<br>Calibration curve: 43.2079 * x + -5281.14<br>Response type: External Std, Area<br>Curve type: Linear, Origin: Exclude, Weighting: 1/x, Axis trans: None<br>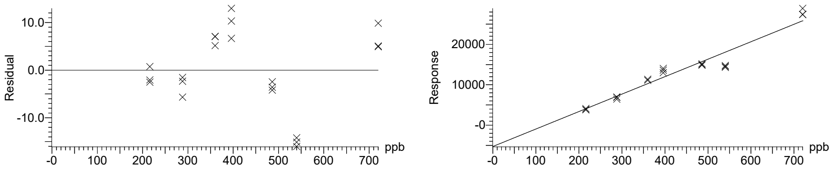
		3	Compound name: PC_756.5<br>Correlation coefficient: r = 0.986049, r^2 = 0.972293<br>Calibration curve: 59.1599 * x + -6493.77<br>Response type: External Std, Area<br>Curve type: Linear, Origin: Exclude, Weighting: 1/x, Axis trans: None<br>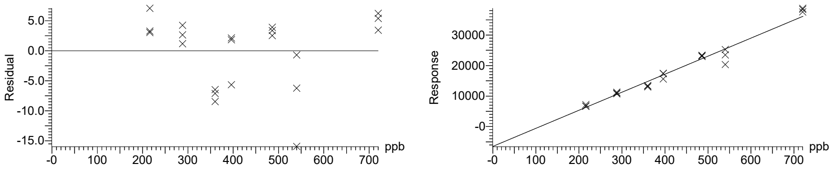

Supplementary Table 8. Calibration curves and residual plots of all the quantified substances (continuation)

Formulation	Molecule	Rep.	Residual plots and calibration curves
SD-noPEG-PSA (continuation)	Phosphatidylcholine 758.6 Da	1	Compound name: PC_758.5<br>Correlation coefficient: r = 0.978454, r^2 = 0.957372<br>Calibration curve: 360.953 * x + -52734.3<br>Response type: External Std, Area<br>Curve type: Linear, Origin: Exclude, Weighting: 1/x, Axis trans: None<br>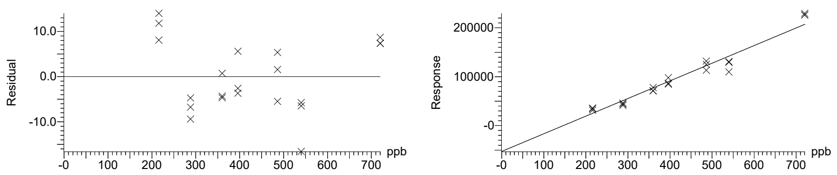
		2	Compound name: PC_758.5<br>Correlation coefficient: r = 0.971378, r^2 = 0.943575<br>Calibration curve: 341.841 * x + -39868.1<br>Response type: External Std, Area<br>Curve type: Linear, Origin: Exclude, Weighting: 1/x, Axis trans: None<br>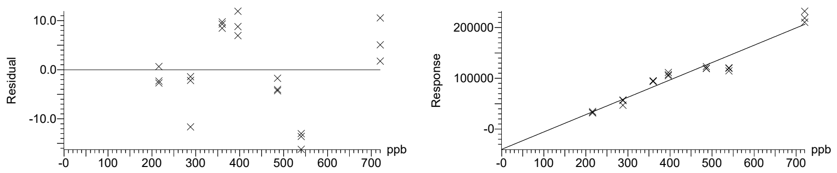
		3	Compound name: PC_758.5<br>Correlation coefficient: r = 0.978576, r^2 = 0.957611<br>Calibration curve: 482.411 * x + -49778.1<br>Response type: External Std, Area<br>Curve type: Linear, Origin: Exclude, Weighting: 1/x, Axis trans: None<br>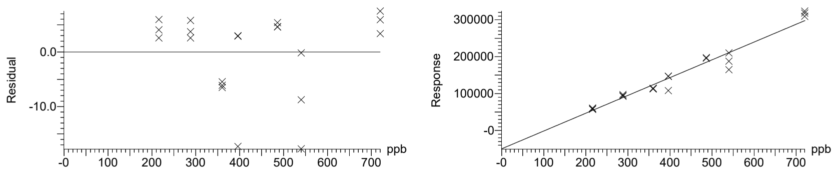
	Phosphatidylcholine 780.6 Da	1	Compound name: PC_780.5<br>Correlation coefficient: r = 0.991907, r^2 = 0.983880<br>Calibration curve: 191.822 * x + -28502.3<br>Response type: External Std, Area<br>Curve type: Linear, Origin: Exclude, Weighting: 1/x, Axis trans: None<br>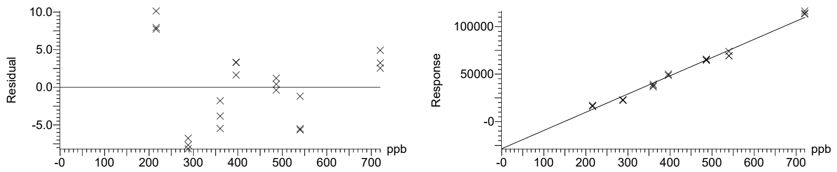
		2	Compound name: PC_780.5<br>Correlation coefficient: r = 0.975548, r^2 = 0.951693<br>Calibration curve: 162.687 * x + -19690.5<br>Response type: External Std, Area<br>Curve type: Linear, Origin: Exclude, Weighting: 1/x, Axis trans: None<br>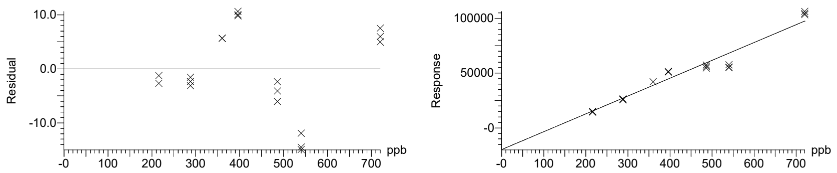
		3	Compound name: PC_780.5<br>Correlation coefficient: r = 0.989517, r^2 = 0.979145<br>Calibration curve: 219.467 * x + -25771.8<br>Response type: External Std, Area<br>Curve type: Linear, Origin: Exclude, Weighting: 1/x, Axis trans: None<br>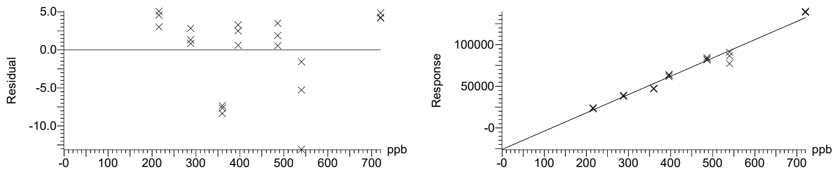

Supplementary Table 8. Calibration curves and residual plots of all the quantified substances (continuation)

Formulation	Molecule	Rep.	Residual plots and calibration curves
SD-noPEG-PSA (continuation)	Phosphatidylcholine 782.5 Da	1	Compound name: PC_782.5<br>Correlation coefficient: r = 0.982009, r^2 = 0.964341<br>Calibration curve: 895.531 * x + -134281<br>Response type: External Std, Area<br>Curve type: Linear, Origin: Exclude, Weighting: 1/x, Axis trans: None<br>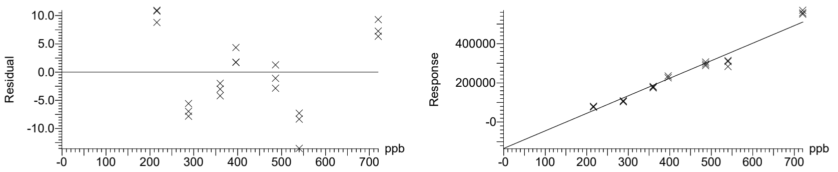
		2	Compound name: PC_782.5<br>Correlation coefficient: r = 0.974672, r^2 = 0.949985<br>Calibration curve: 815.527 * x + -100263<br>Response type: External Std, Area<br>Curve type: Linear, Origin: Exclude, Weighting: 1/x, Axis trans: None<br>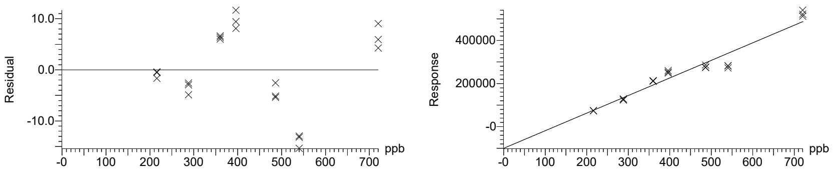
		3	Compound name: PC_782.5<br>Correlation coefficient: r = 0.986556, r^2 = 0.973292<br>Calibration curve: 1101.46 * x + -122320<br>Response type: External Std, Area<br>Curve type: Linear, Origin: Exclude, Weighting: 1/x, Axis trans: None<br>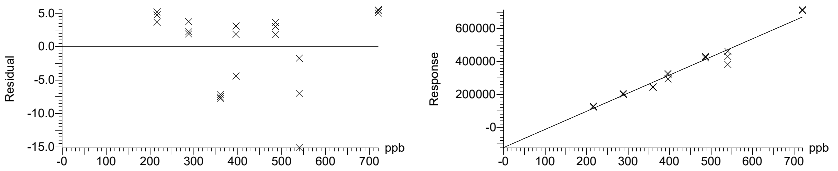
	Polysialic acid (whole)	1	Compound name: Digested polysialic acid<br>Correlation coefficient: r = 0.984542, r^2 = 0.969323<br>Calibration curve: 495.967 * x + -528.657<br>Response type: External Std, Area<br>Curve type: Linear, Origin: Exclude, Weighting: 1/x, Axis trans: None<br>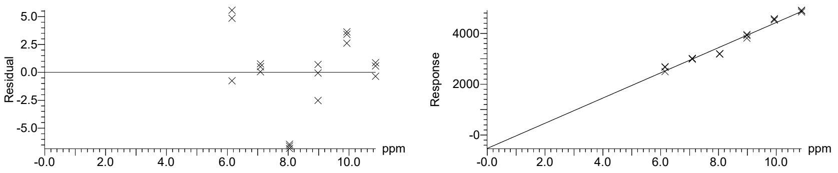
		2	Compound name: Digested polysialic acid<br>Correlation coefficient: r = 0.995783, r^2 = 0.991583<br>Calibration curve: 474.707 * x + -2.33855<br>Response type: External Std, Area<br>Curve type: Linear, Origin: Exclude, Weighting: 1/x, Axis trans: None<br>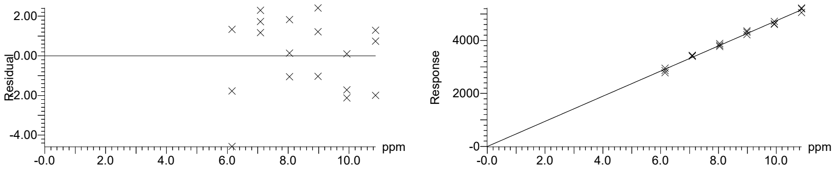
		3	Compound name: Digested polysialic acid<br>Correlation coefficient: r = 0.987737, r^2 = 0.975625<br>Calibration curve: 477.285 * x + -592.32<br>Response type: External Std, Area<br>Curve type: Linear, Origin: Exclude, Weighting: 1/x, Axis trans: None<br>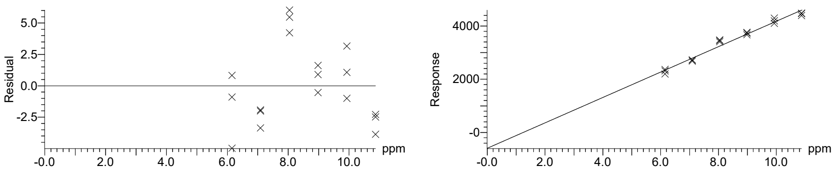

Supplementary Table 8. Calibration curves and residual plots of all the quantified substances (continuation)

Formulation	Molecule	Rep.	Residual plots and calibration curves
SD-noPEG-PSA (continuation)	Polysialic acid (supernatant)	1	Compound name: Digested polysialic acid<br>Correlation coefficient: r = 0.996813, r^2 = 0.993635<br>Calibration curve: 215.458 * x + -41.1843<br>Response type: External Std, Area<br>Curve type: Linear, Origin: Exclude, Weighting: 1/x, Axis trans: None<br>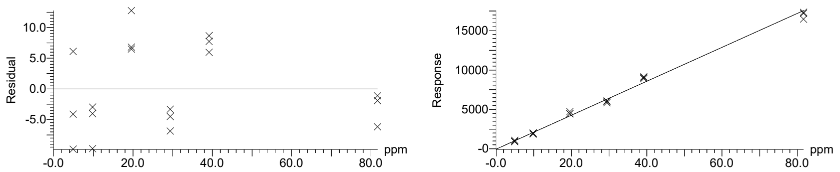
		2	Compound name: Digested polysialic acid<br>Correlation coefficient: r = 0.991058, r^2 = 0.982196<br>Calibration curve: 336.608 * x + -508.845<br>Response type: External Std, Area<br>Curve type: Linear, Origin: Exclude, Weighting: 1/x, Axis trans: None<br>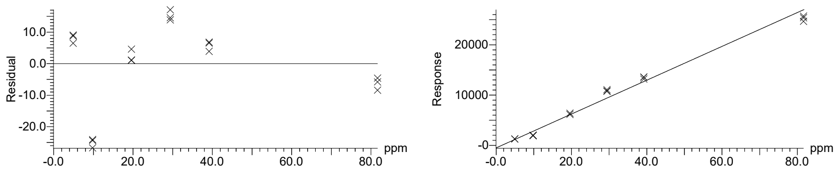
		3	Compound name: Digested polysialic acid<br>Correlation coefficient: r = 0.992138, r^2 = 0.984337<br>Calibration curve: 278.905 * x + -449.887<br>Response type: External Std, Area<br>Curve type: Linear, Origin: Exclude, Weighting: 1/x, Axis trans: None<br>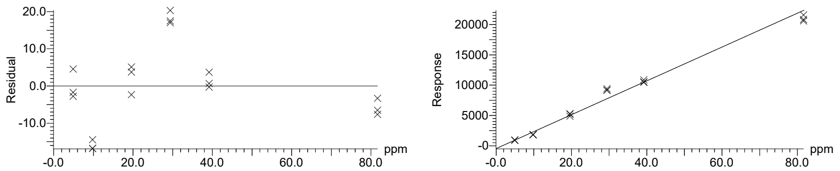
	Polysialic acid (infranatant)	1	Compound name: Digested polysialic acid<br>Correlation coefficient: r = 0.998050, r^2 = 0.996104<br>Calibration curve: 328.803 * x + 27.6584<br>Response type: External Std, Area<br>Curve type: Linear, Origin: Exclude, Weighting: 1/x, Axis trans: None<br>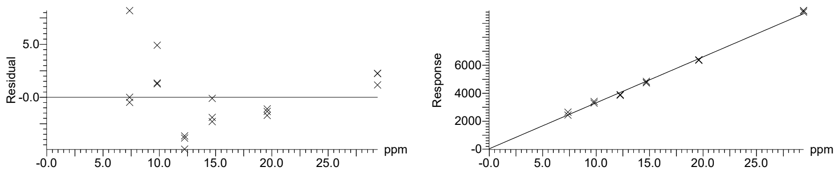
		2	Compound name: Digested polysialic acid<br>Correlation coefficient: r = 0.997997, r^2 = 0.995998<br>Calibration curve: 441.915 * x + -248.6<br>Response type: External Std, Area<br>Curve type: Linear, Origin: Exclude, Weighting: 1/x, Axis trans: None<br>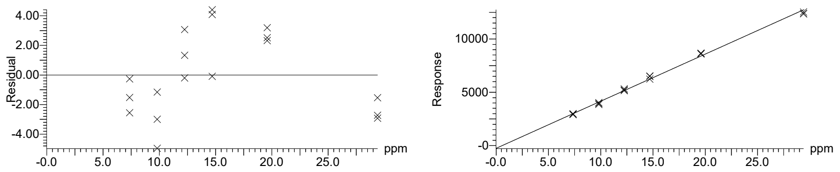
		3	Compound name: Digested polysialic acid<br>Correlation coefficient: r = 0.993285, r^2 = 0.986614<br>Calibration curve: 352.887 * x + 329.055<br>Response type: External Std, Area<br>Curve type: Linear, Origin: Exclude, Weighting: 1/x, Axis trans: None<br>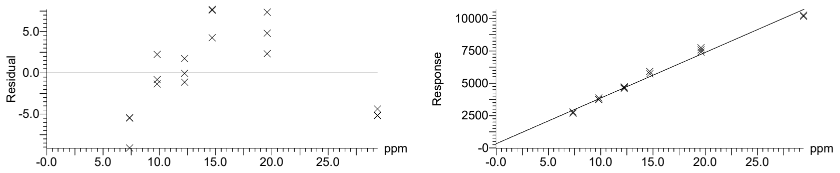

SUPPORTING INFORMATION | Quantification of the actual composition of polymeric nanocapsules. A quality control analysis

Supplementary Table 8. Calibration curves and residual plots of all the quantified substances (continuation)

Formulation	Molecule	Rep.	Residual plots and calibration curves
SE-*b*-PEG-HA	Benzethonium	1	Compound name: benzethonium<br>Correlation coefficient: r = 0.999809, r^2 = 0.999617<br>Calibration curve: 20837.2 * x + 8846.54<br>Response type: External Std, Area<br>Curve type: Linear, Origin: Exclude, Weighting: 1/x, Axis trans: None<br>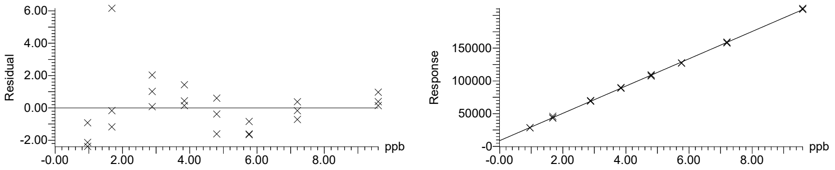
		2	Compound name: benzethonium<br>Correlation coefficient: r = 0.999915, r^2 = 0.999830<br>Calibration curve: 28497.7 * x + 9350.55<br>Response type: External Std, Area<br>Curve type: Linear, Origin: Exclude, Weighting: 1/x, Axis trans: None<br>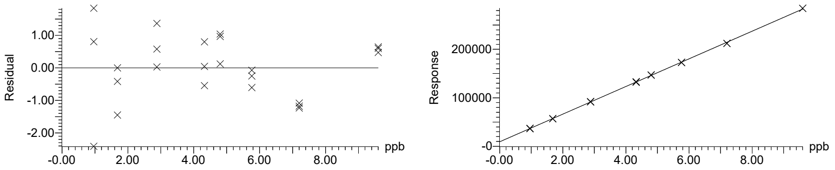
		3	Compound name: benzethonium<br>Correlation coefficient: r = 0.999769, r^2 = 0.999538<br>Calibration curve: 21075.3 * x + 18948<br>Response type: External Std, Area<br>Curve type: Linear, Origin: Exclude, Weighting: 1/x, Axis trans: None<br>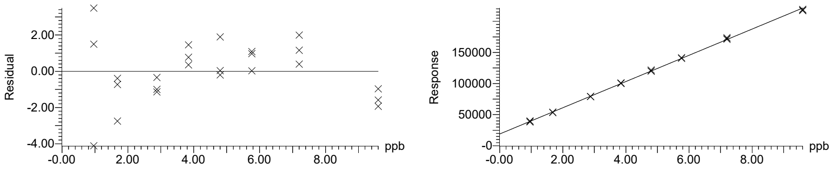
	Macrogol-15-hydroxystearate	1	Compound name: macrogol-15-hydroxystearate<br>Correlation coefficient: r = 0.999616, r^2 = 0.999233<br>Calibration curve: 490.537 * x + 621.962<br>Response type: External Std, Area<br>Curve type: Linear, Origin: Exclude, Weighting: 1/x, Axis trans: None<br>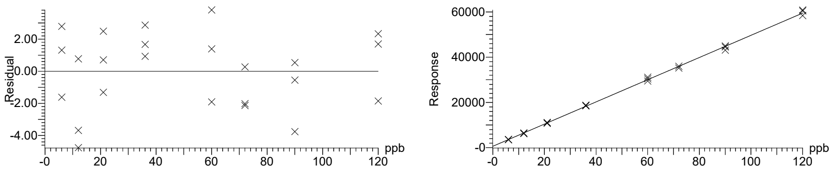
		2	Compound name: macrogol-15-hydroxystearate<br>Correlation coefficient: r = 0.999613, r^2 = 0.999226<br>Calibration curve: 552.297 * x + 1523.58<br>Response type: External Std, Area<br>Curve type: Linear, Origin: Exclude, Weighting: 1/x, Axis trans: None<br>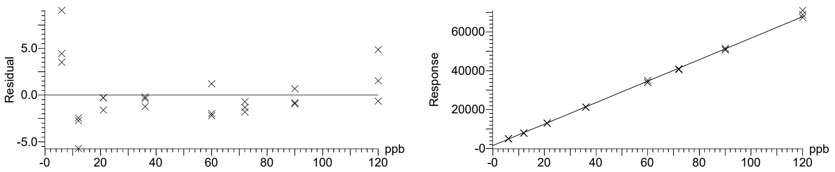
		3	Compound name: macrogol-15-hydroxystearate<br>Correlation coefficient: r = 0.999533, r^2 = 0.999066<br>Calibration curve: 492.173 * x + 1525.34<br>Response type: External Std, Area<br>Curve type: Linear, Origin: Exclude, Weighting: 1/x, Axis trans: None<br>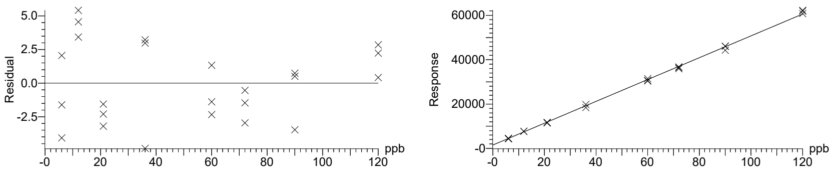

Supplementary Table 8. Calibration curves and residual plots of all the quantified substances (continuation)

Formulation	Molecule	Rep.	Residual plots and calibration curves
SE-*b*-PEG-HA (continuation)	Polysorbate 80	1	Compound name: polysorbate 80<br>Correlation coefficient: r = 0.999571, r^2 = 0.999142<br>Calibration curve: 21.7494 * x + -5484.45<br>Response type: External Std, Area<br>Curve type: Linear, Origin: Exclude, Weighting: 1/x, Axis trans: None<br>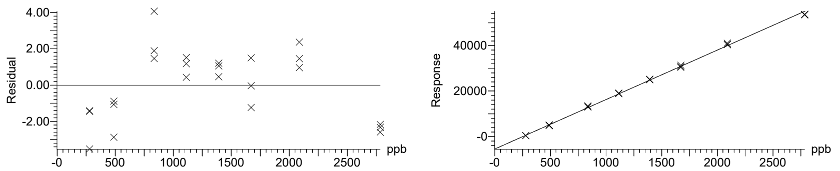
		2	Compound name: polysorbate 80<br>Correlation coefficient: r = 0.999600, r^2 = 0.999200<br>Calibration curve: 27.0221 * x + -7565.95<br>Response type: External Std, Area<br>Curve type: Linear, Origin: Exclude, Weighting: 1/x, Axis trans: None<br>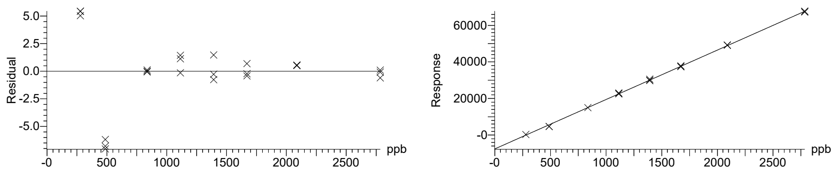
		3	Compound name: polysorbate 80<br>Correlation coefficient: r = 0.999386, r^2 = 0.998773<br>Calibration curve: 24.6304 * x + -4631.83<br>Response type: External Std, Area<br>Curve type: Linear, Origin: Exclude, Weighting: 1/x, Axis trans: None<br>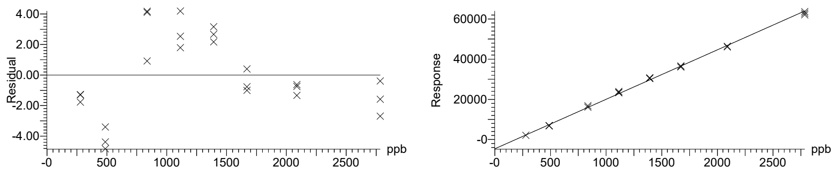
	C_8-C_8-C_8 Triglyceride	1	Compound name: C8-C8-C8 TG<br>Correlation coefficient: r = 0.998555, r^2 = 0.997112<br>Calibration curve: 1143.7 * x + 729914<br>Response type: External Std, Area<br>Curve type: Linear, Origin: Exclude, Weighting: 1/x, Axis trans: None<br>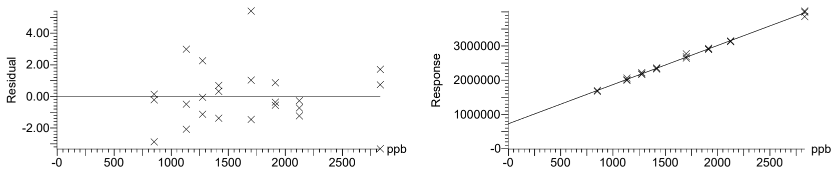
		2	Compound name: C8-C8-C8 TG<br>Correlation coefficient: r = 0.996395, r^2 = 0.992802<br>Calibration curve: 1161.24 * x + 857869<br>Response type: External Std, Area<br>Curve type: Linear, Origin: Exclude, Weighting: 1/x, Axis trans: None<br>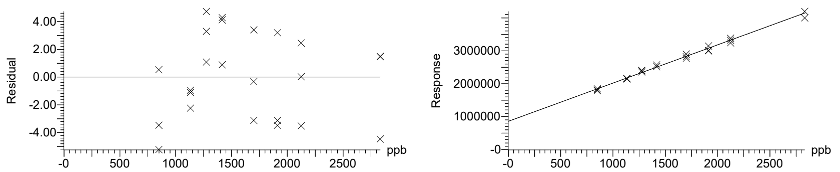
		3	Compound name: C8-C8-C8 TG<br>Correlation coefficient: r = 0.995926, r^2 = 0.991868<br>Calibration curve: 1236.69 * x + 836793<br>Response type: External Std, Area<br>Curve type: Linear, Origin: Exclude, Weighting: 1/x, Axis trans: None<br>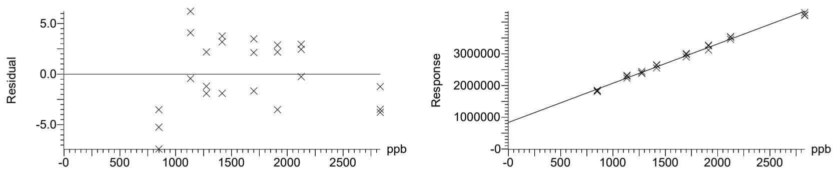

Supplementary Table 8. Calibration curves and residual plots of all the quantified substances (continuation)

Formulation	Molecule	Rep.	Residual plots and calibration curves
SE-*b*-PEG-HA (continuation)	C_8-C_8-C_{10} Triglyceride	1	Compound name: C8-C8-C10 TG<br>Correlation coefficient: r = 0.995759, r^2 = 0.991536<br>Calibration curve: 1340.75 * x + 1.08877e+006<br>Response type: External Std, Area<br>Curve type: Linear, Origin: Exclude, Weighting: 1/x, Axis trans: None<br>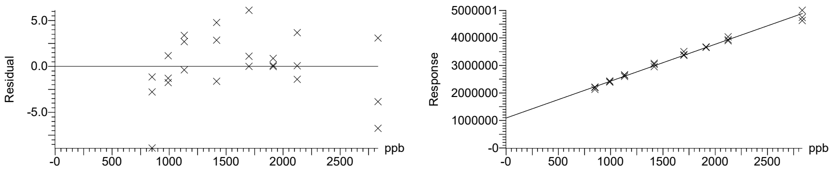
		2	Compound name: C8-C8-C10 TG<br>Correlation coefficient: r = 0.995234, r^2 = 0.990491<br>Calibration curve: 1399.21 * x + 1.14637e+006<br>Response type: External Std, Area<br>Curve type: Linear, Origin: Exclude, Weighting: 1/x, Axis trans: None<br>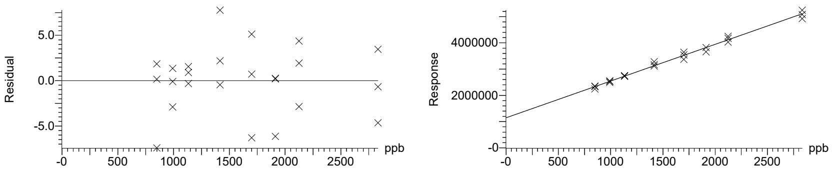
		3	Compound name: C8-C10-C10 TG<br>Correlation coefficient: r = 0.992999, r^2 = 0.986048<br>Calibration curve: 1067.63 * x + 847138<br>Response type: External Std, Area<br>Curve type: Linear, Origin: Exclude, Weighting: 1/x, Axis trans: None<br>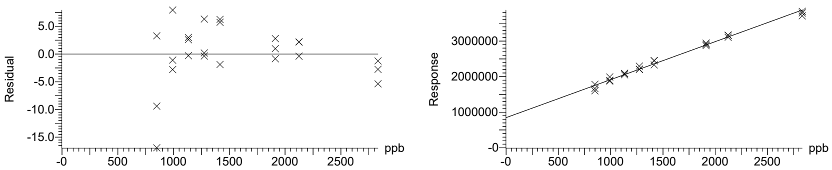
	C_8-C_{10}-C_{10} Triglyceride	1	Compound name: C8-C10-C10 TG<br>Correlation coefficient: r = 0.995371, r^2 = 0.990763<br>Calibration curve: 1042.33 * x + 675228<br>Response type: External Std, Area<br>Curve type: Linear, Origin: Exclude, Weighting: 1/x, Axis trans: None<br>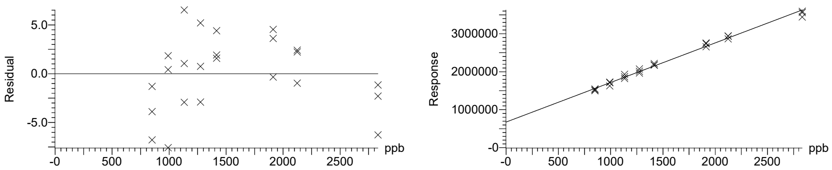
		2	Compound name: C8-C10-C10 TG<br>Correlation coefficient: r = 0.995793, r^2 = 0.991604<br>Calibration curve: 987.866 * x + 787402<br>Response type: External Std, Area<br>Curve type: Linear, Origin: Exclude, Weighting: 1/x, Axis trans: None<br>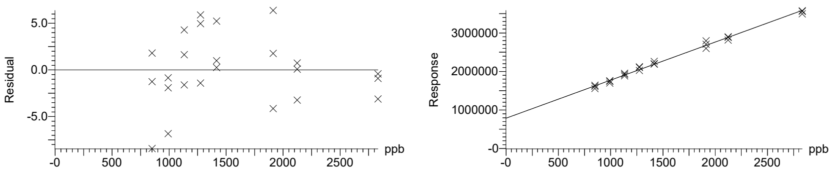
		3	Compound name: C8-C10-C10 TG<br>Correlation coefficient: r = 0.990811, r^2 = 0.981706<br>Calibration curve: 1562.36 * x + 603095<br>Response type: External Std, Area<br>Curve type: Linear, Origin: Exclude, Weighting: 1/x, Axis trans: None<br>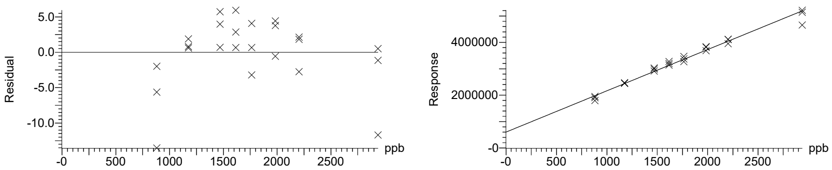

Supplementary Table 8. Calibration curves and residual plots of all the quantified substances (continuation)

Formulation	Molecule	Rep.	Residual plots and calibration curves
SE-*b*-PEG-HA (continuation)	C_{10}-C_{10}-C_{10} Triglyceride	1	Compound name: C10-C10-C10 TG<br>Correlation coefficient: r = 0.995952, r^2 = 0.991921<br>Calibration curve: 422.591 * x + 167207<br>Response type: External Std, Area<br>Curve type: Linear, Origin: Exclude, Weighting: 1/x, Axis trans: None<br>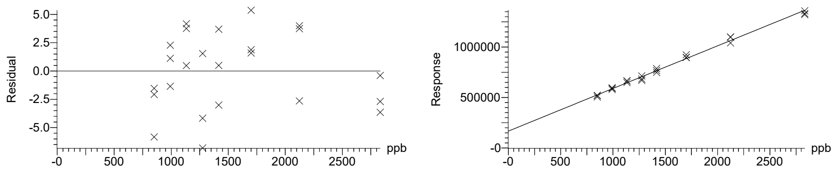
		2	Compound name: C10-C10-C10 TG<br>Correlation coefficient: r = 0.991788, r^2 = 0.983644<br>Calibration curve: 420.378 * x + 185286<br>Response type: External Std, Area<br>Curve type: Linear, Origin: Exclude, Weighting: 1/x, Axis trans: None<br>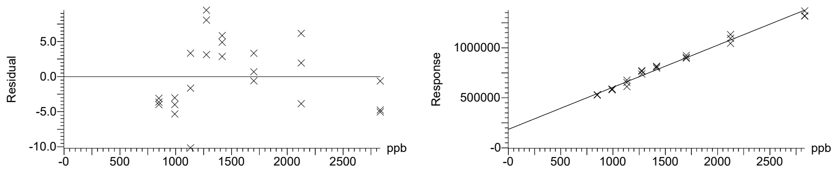
		3	Compound name: C10-C10-C10 TG<br>Correlation coefficient: r = 0.990394, r^2 = 0.980880<br>Calibration curve: 452.742 * x + 206773<br>Response type: External Std, Area<br>Curve type: Linear, Origin: Exclude, Weighting: 1/x, Axis trans: None<br>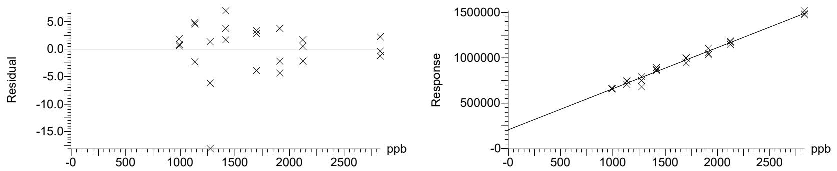
	Hyaluronic acid (whole)	1	Compound name: Digested hyaluronic acid<br>Correlation coefficient: r = 0.993235, r^2 = 0.986517<br>Calibration curve: 4187.56 * x + -16548<br>Response type: External Std, Area<br>Curve type: Linear, Origin: Exclude, Weighting: 1/x, Axis trans: None<br>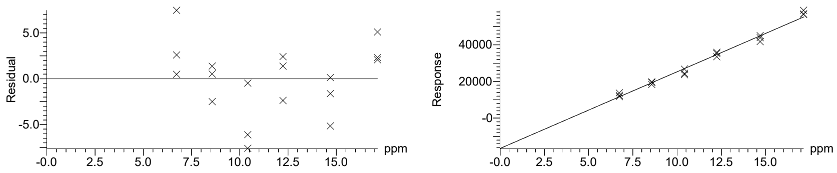
		2	Compound name: Digested hyaluronic acid<br>Correlation coefficient: r = 0.992919, r^2 = 0.985888<br>Calibration curve: 5760.23 * x + -22313.5<br>Response type: External Std, Area<br>Curve type: Linear, Origin: Exclude, Weighting: 1/x, Axis trans: None<br>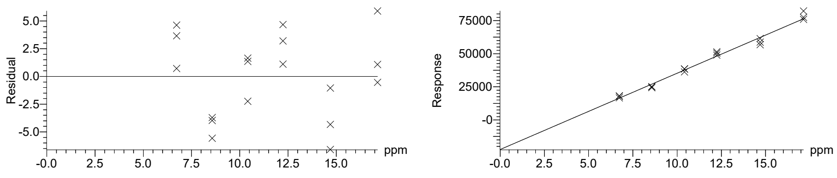
		3	Compound name: Digested hyaluronic acid<br>Correlation coefficient: r = 0.993213, r^2 = 0.986472<br>Calibration curve: 5010.39 * x + -12794.7<br>Response type: External Std, Area<br>Curve type: Linear, Origin: Exclude, Weighting: 1/x, Axis trans: None<br>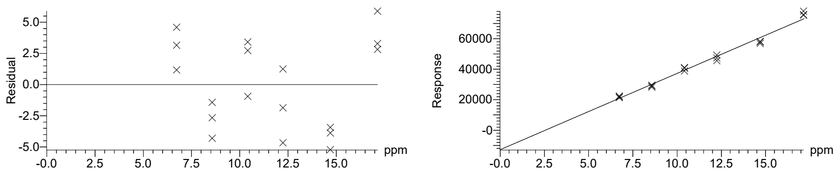

Supplementary Table 8. Calibration curves and residual plots of all the quantified substances (continuation)

Formulation	Molecule	Rep.	Residual plots and calibration curves
SE-*b*-PEG-HA (continuation)	Hyaluronic acid (retentate)	1	Compound name: Digested hyaluronic acid<br>Correlation coefficient: r = 0.984484, r^2 = 0.969209<br>Calibration curve: 2948.95 * x + -7474<br>Response type: External Std, Area<br>Curve type: Linear, Origin: Exclude, Weighting: 1/x, Axis trans: None<br>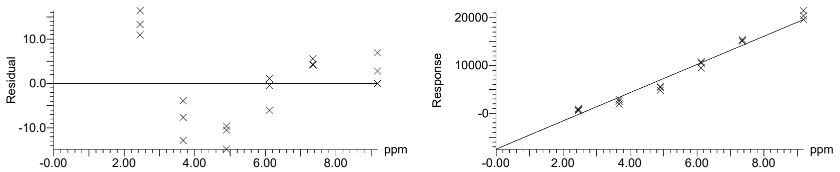
		2	Compound name: Digested hyaluronic acid<br>Correlation coefficient: r = 0.990093, r^2 = 0.980285<br>Calibration curve: 3985.69 * x + -7849.32<br>Response type: External Std, Area<br>Curve type: Linear, Origin: Exclude, Weighting: 1/x, Axis trans: None<br>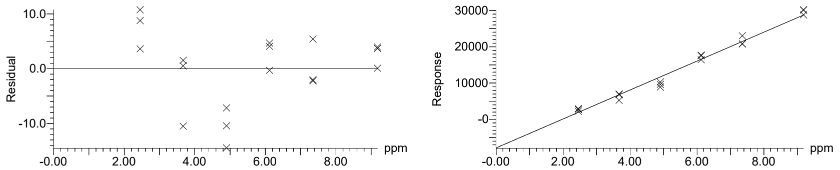
		3	Compound name: Digested hyaluronic acid<br>Correlation coefficient: r = 0.973670, r^2 = 0.948034<br>Calibration curve: 3972.57 * x + -8904.86<br>Response type: External Std, Area<br>Curve type: Linear, Origin: Exclude, Weighting: 1/x, Axis trans: None<br>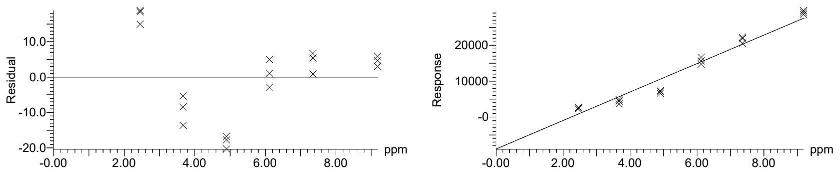
	Hyaluronic acid (permeate)	1	Compound name: Digested hyaluronic acid<br>Correlation coefficient: r = 0.990870, r^2 = 0.981823<br>Calibration curve: 2674.08 * x + -9315.04<br>Response type: External Std, Area<br>Curve type: Linear, Origin: Exclude, Weighting: 1/x, Axis trans: None<br>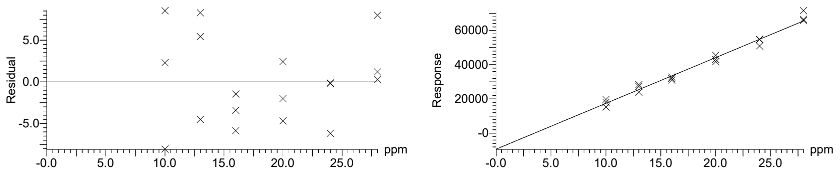
		2	Compound name: Digested hyaluronic acid<br>Correlation coefficient: r = 0.996754, r^2 = 0.993519<br>Calibration curve: 2756.76 * x + -7074.99<br>Response type: External Std, Area<br>Curve type: Linear, Origin: Exclude, Weighting: 1/x, Axis trans: None<br>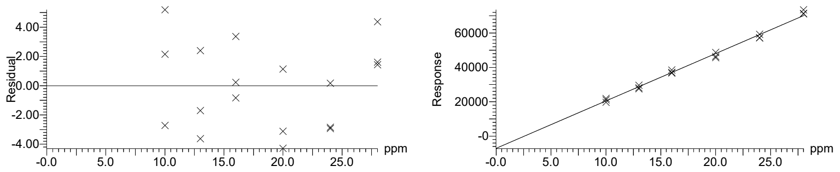
		3	Compound name: Digested hyaluronic acid<br>Correlation coefficient: r = 0.996494, r^2 = 0.993000<br>Calibration curve: 2247.5 * x + -6382.66<br>Response type: External Std, Area<br>Curve type: Linear, Origin: Exclude, Weighting: 1/x, Axis trans: None<br>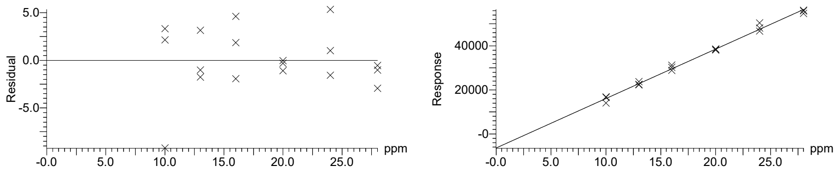

Supplementary Table 8. Calibration curves and residual plots of all the quantified substances (continuation)

Formulation	Molecule	Rep.	Residual plots and calibration curves
SD-noPEG-PEG·PGA	DL-α-tocopherol	1	Compound name: DL-α-tocopherol<br>Correlation coefficient: r = 0.997469, r^2 = 0.994945<br>Calibration curve: 49.4472 * x + -3461.7<br>Response type: External Std, Area<br>Curve type: Linear, Origin: Exclude, Weighting: 1/x, Axis trans: None<br>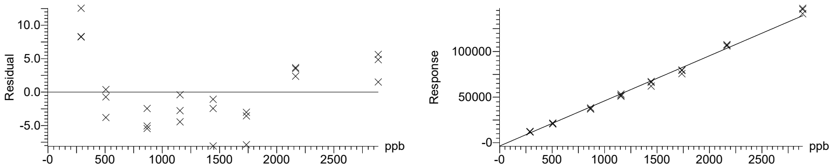
		2	Compound name: DL-α-tocopherol<br>Correlation coefficient: r = 0.998434, r^2 = 0.996871<br>Calibration curve: 46.7148 * x + -2340.53<br>Response type: External Std, Area<br>Curve type: Linear, Origin: Exclude, Weighting: 1/x, Axis trans: None<br>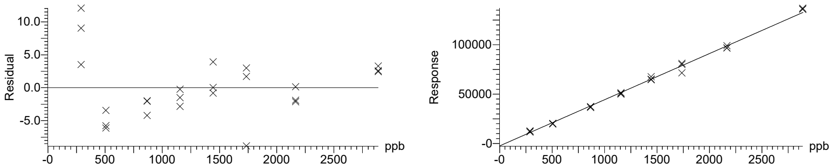
		3	Compound name: DL-α-tocopherol<br>Correlation coefficient: r = 0.997046, r^2 = 0.994100<br>Calibration curve: 48.3276 * x + -3138.24<br>Response type: External Std, Area<br>Curve type: Linear, Origin: Exclude, Weighting: 1/x, Axis trans: None<br>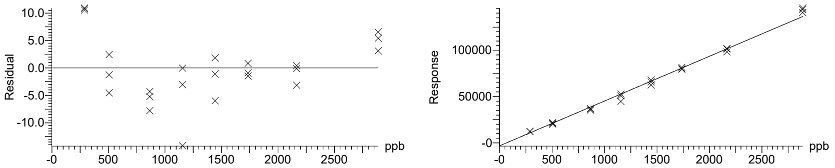
	DOTAP	1	Compound name: 1,2-dioleoyl-3-trimethylammoniumpropane<br>Correlation coefficient: r = 0.999393, r^2 = 0.998787<br>Calibration curve: 1851.7 * x + -1312.06<br>Response type: External Std, Area<br>Curve type: Linear, Origin: Exclude, Weighting: 1/x, Axis trans: None<br>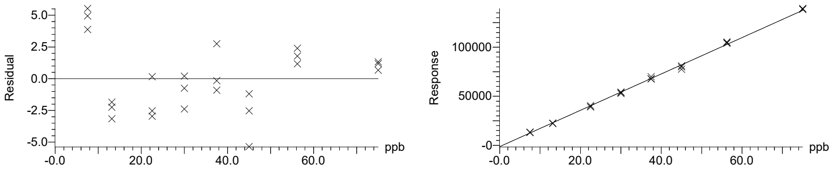
		2	Compound name: 1,2-dioleoyl-3-trimethylammoniumpropane<br>Correlation coefficient: r = 0.999553, r^2 = 0.999106<br>Calibration curve: 1673.68 * x + 86.2758<br>Response type: External Std, Area<br>Curve type: Linear, Origin: Exclude, Weighting: 1/x, Axis trans: None<br>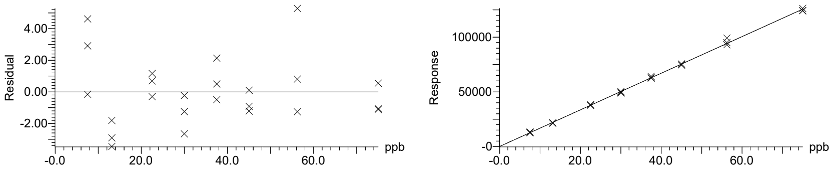
		3	Compound name: 1,2-dioleoyl-3-trimethylammoniumpropane<br>Correlation coefficient: r = 0.998635, r^2 = 0.997271<br>Calibration curve: 1530.23 * x + 253.64<br>Response type: External Std, Area<br>Curve type: Linear, Origin: Exclude, Weighting: 1/x, Axis trans: None<br>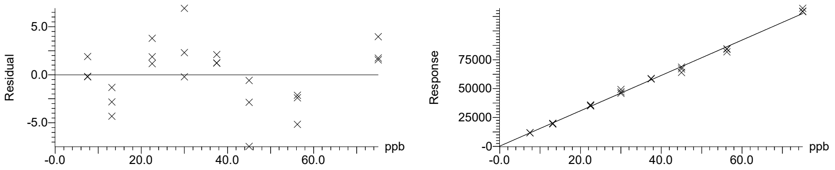

Supplementary Table 8. Calibration curves and residual plots of all the quantified substances (continuation)

Formulation	Molecule	Rep.	Residual plots and calibration curves
SD-noPEG-PEG·PGA (continuation)	PEG·PGA (whole)	1	Compound name: Digested polyethylene glycol polyglutamic acid<br>Correlation coefficient: r = 0.998487, r^2 = 0.996977<br>Calibration curve: 10575.9 * x + -30283.5<br>Response type: External Std, Area<br>Curve type: Linear, Origin: Exclude, Weighting: 1/x, Axis trans: None<br>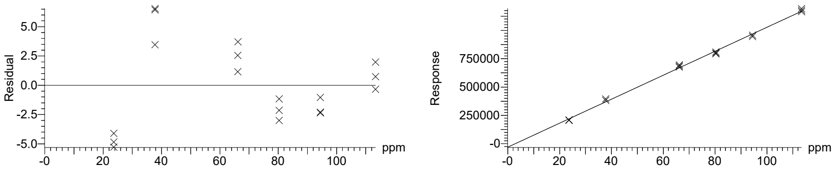
		2	Compound name: Digested polyethylene glycol polyglutamic acid<br>Correlation coefficient: r = 0.999504, r^2 = 0.999009<br>Calibration curve: 7449.11 * x + 22416.1<br>Response type: External Std, Area<br>Curve type: Linear, Origin: Exclude, Weighting: 1/x, Axis trans: None<br>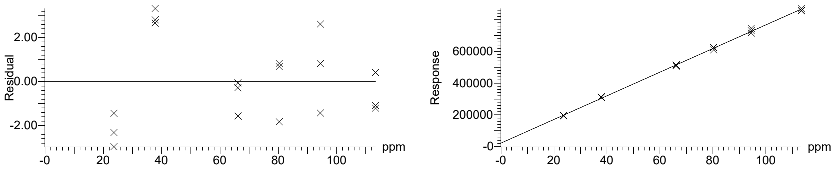
		3	Compound name: Digested polyethylene glycol polyglutamic acid<br>Correlation coefficient: r = 0.998669, r^2 = 0.997340<br>Calibration curve: 8812.53 * x + -7182.38<br>Response type: External Std, Area<br>Curve type: Linear, Origin: Exclude, Weighting: 1/x, Axis trans: None<br>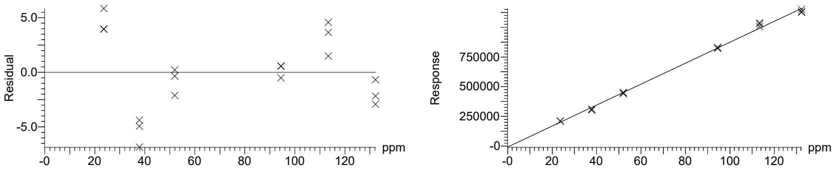
	PEG·PGA (supernatant)	1	Compound name: Digested polyethylene glycol polyglutamic acid<br>Correlation coefficient: r = 0.998201, r^2 = 0.996404<br>Calibration curve: 9707.07 * x + 13837.7<br>Response type: External Std, Area<br>Curve type: Linear, Origin: Exclude, Weighting: 1/x, Axis trans: None<br>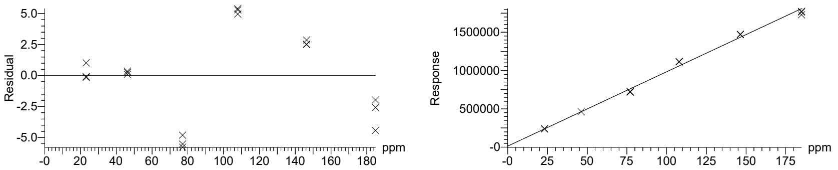
		2	Compound name: Digested polyethylene glycol polyglutamic acid<br>Correlation coefficient: r = 0.999767, r^2 = 0.999534<br>Calibration curve: 7914.54 * x + 12392.4<br>Response type: External Std, Area<br>Curve type: Linear, Origin: Exclude, Weighting: 1/x, Axis trans: None<br>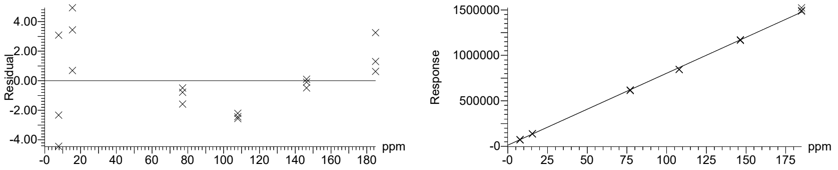
		3	Compound name: Digested polyethylene glycol polyglutamic acid<br>Correlation coefficient: r = 0.998509, r^2 = 0.997020<br>Calibration curve: 7881.39 * x + 20487.5<br>Response type: External Std, Area<br>Curve type: Linear, Origin: Exclude, Weighting: 1/x, Axis trans: None<br>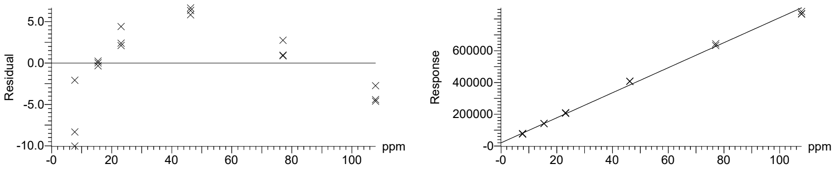

Supplementary Table 8. Calibration curves and residual plots of all the quantified substances (continuation)

Formulation	Molecule	Rep.	Residual plots and calibration curves
SD-noPEG-PEG·PGA (continuation)	PEG·PGA (infranatant)	1	Compound name: Digested polyethylene glycol polyglutamic acid<br>Correlation coefficient: r = 0.998736, r^2 = 0.997474<br>Calibration curve: 11304.1 * x + 8801.48<br>Response type: External Std, Area<br>Curve type: Linear, Origin: Exclude, Weighting: 1/x, Axis trans: None<br>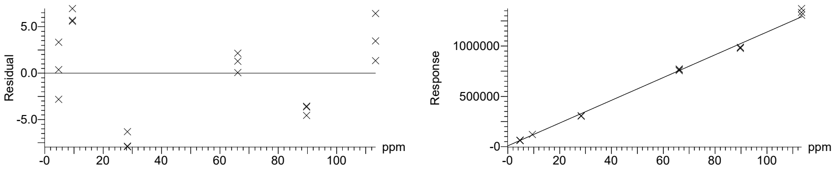
		2	Compound name: Digested polyethylene glycol polyglutamic acid<br>Correlation coefficient: r = 0.999069, r^2 = 0.998139<br>Calibration curve: 8114.2 * x + 3596.93<br>Response type: External Std, Area<br>Curve type: Linear, Origin: Exclude, Weighting: 1/x, Axis trans: None<br>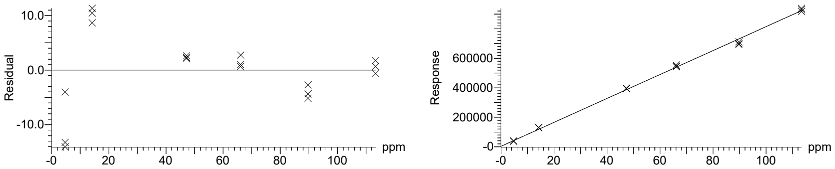
		3	Compound name: Digested polyethylene glycol polyglutamic acid<br>Correlation coefficient: r = 0.999902, r^2 = 0.999803<br>Calibration curve: 8230.45 * x + 11492.7<br>Response type: External Std, Area<br>Curve type: Linear, Origin: Exclude, Weighting: 1/x, Axis trans: None<br>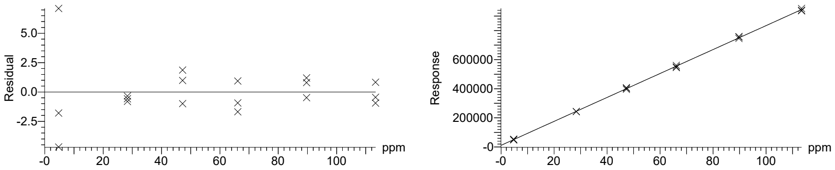

SD-*l*-PEG-HA: lineal-polyethylene glycol-containing hyaluronic acid nanocapsules formulated by solvent displacement. SD-noPEG-HA: non-PEGylated hyaluronic acid nanocapsules formulated by solvent displacement. SD-noPEG-PSA: non-PEGylated polysialic acid nanocapsules formulated by solvent displacement. SE-*b*-PEG-HA: Self-emulsifying branched-polyethylene glycol-containing hyaluronic acid nanocapsules. SD-noPEG-PEG·PGA: Solvent displacement nanocapsules not containing PEGylated surfactants and with an outer shell of polyethylene glycol polyglutamic acid. Residuals were plotted as percentual relative residuals (%RRES) vs. concentration, while calibration curves, response vs. concentration. In both cases, a weighting factor of 1/x was applied. In all cases, the mean of the triplicates of the %RRES for each concentration level was found to be in the range of – 20 % to 20 %, confirming the linearity of the calibration curve. In general, residuals followed a random distribution around the value %RRES = 0. Rep.: replicates.

Supplementary Table 9. Mass percentage detected in each fraction relative to the theoretical amount added to the formulation

Formulation	Substance	Recovery before isolation (%)	Supernatant / retentate recovery (%)	Infranatant / permeate recovery (%)	Sum of the fractions' recoveries (%)
SD-*l*-PEG-HA	Benzethonium	92 ± 10	39 ± 5	56 ± 4	95 ± 9
	DL-α-tocopherol	106 ± 10	72 ± 5	30 ± 5	102 ± 10
	D-α-tocopherol polyethylene glycol 1000 succinate	103 ± 10	58 ± 6	43 ± 6	101 ± 12
	Hyaluronic acid	100 ± 5	18 ± 3	86 ± 2	104 ± 5
SD-noPEG-HA	Hexadecyltrimethylammonium	99 ± 19	35 ± 8	42 ± 9	77 ± 16
	C_8-C_8-C_8 Triglyceride	113 ± 8	52 ± 3	24 ± 3	75 ± 6
	C_8-C_8-C_{10} Triglyceride	109 ± 7	53 ± 4	25 ± 4	78 ± 7
	C_8-C_{10}-C_{10} Triglyceride	109 ± 8	53 ± 4	24 ± 4	77 ± 7
	C_{10}-C_{10}-C_{10} Triglyceride	110 ± 10	54 ± 5	23 ± 4	77 ± 9
	Phosphatidylcholine 756.5 Da	96 ± 14	16 ± 3	27 ± 9	43 ± 11
	Phosphatidylcholine 758.5 Da	98 ± 18	16 ± 3	27 ± 9	43 ± 12
	Phosphatidylcholine 780.5 Da	97 ± 12	16 ± 3	25 ± 10	42 ± 13
	Phosphatidylcholine 782.5 Da	97 ± 14	16 ± 3	27 ± 9	44 ± 12
	Hyaluronic acid	105 ± 7	14 ± 2	80 ± 7	95 ± 8
SD-noPEG-PSA	Hexadecyltrimethylammonium	103 ± 11	31 ± 4	59 ± 6	90 ± 9
	C_8-C_8-C_8 Triglyceride	101 ± 10	68 ± 8	28 ± 5	95 ± 13
	C_8-C_8-C_{10} Triglyceride	100 ± 12	66 ± 8	28 ± 5	94 ± 13
	C_8-C_{10}-C_{10} Triglyceride	99 ± 11	66 ± 8	28 ± 4	94 ± 13
	C_{10}-C_{10}-C_{10} Triglyceride	98 ± 12	64 ± 7	30 ± 5	94 ± 12
	Phosphatidylcholine 756.5 Da	96 ± 8	17 ± 3	40 ± 8	57 ± 11
	Phosphatidylcholine 758.5 Da	94 ± 9	18 ± 3	40 ± 8	58 ± 11
	Phosphatidylcholine 780.5 Da	98 ± 9	18 ± 2	41 ± 9	59 ± 11
	Phosphatidylcholine 782.5 Da	97 ± 7	17 ± 3	40 ± 8	58 ± 11
	Polysialic acid	103 ± 2	14 ± 1	91 ± 3	104 ± 4
SE-*b*-PEG-HA	Polyethylene glycol	103 ± 9	< 10	96 ± 13	N/A
	Benzethonium	109 ± 4	51 ± 12	51 ± 8	102 ± 20
	Macrogol-15-hydroxystearate	99 ± 6	39 ± 5	54 ± 11	93 ± 16
	Polysorbate 80	111 ± 4	24 ± 9	63 ± 6	87 ± 15
	C_8-C_8-C_8 Triglyceride	106 ± 5	95 ± 7	< 10	N/A
	C_8-C_8-C_{10} Triglyceride	108 ± 6	95 ± 6	< 10	N/A
	C_8-C_{10}-C_{10} Triglyceride	107 ± 6	94 ± 6	< 10	N/A
	C_{10}-C_{10}-C_{10} Triglyceride	104 ± 10	89 ± 8	< 10	N/A
	Hyaluronic acid	91 ± 3	43 ± 4	53 ± 3	96 ± 7
SD-noPEG-PEG·PGA	DL-α-tocopherol	96 ± 4	95 ± 6	9 ± 3	103 ± 8
	1,2-dioleoyl-3-trimethylammoniumpropane	84 ± 8	89 ± 3	12 ± 3	101 ± 6
	PEG·PGA	109 ± 11	34 ± 9	67 ± 5	101 ± 15

SD-*l*-PEG-HA: lineal-polyethylene glycol-containing hyaluronic acid nanocapsules formulated by solvent displacement. SD-noPEG-HA: non-PEGylated hyaluronic acid nanocapsules formulated by solvent displacement. SD-noPEG-PSA: non-PEGylated polysialic acid nanocapsules formulated by solvent displacement. SE-*b*-PEG-HA: Self-emulsifying branched-polyethylene glycol-containing hyaluronic acid nanocapsules. N/A: not applicable. n = 3. Data are shown as mean ± standard deviation.

Supplementary Bibliography

(1) Inoue, T.; Tatemori, S.; Muranaka, N.; Hirahara, Y.; Homma, S.; Nakane, T.; Takano, A.; Nomi, Y.; Otsuka, Y. The Identification of Vitamin e Homologues in Medicinal Plant Samples Using ESI(+)-LC-MS3. *J. Agric. Food Chem.* **2012**, *60* (38), 9581–9588. https://doi.org/10.1021/jf302425z.

(2) Jiang, K.; Gachumi, G.; Poudel, A.; Shurmer, B.; Bashi, Z.; El-Aneed, A. The Establishment of Tandem Mass Spectrometric Fingerprints of Phytosterols and Tocopherols and the Development of Targeted Profiling Strategies in Vegetable Oils. *J. Am. Soc. Mass Spectrom.* **2019**, *30* (9), 1700–1712. https://doi.org/10.1007/s13361-019-02242-2.

(3) LeBouf, R. F.; Virji, M. A.; Ranpara, A.; Stefaniak, A. B. Air and Surface Sampling Method for Assessing Exposures to Quaternary Ammonium Compounds Using Liquid Chromatography Tandem Mass Spectrometry. *Ann. Work Expo. Heal.* **2017**, *61* (6), 724–736. https://doi.org/10.1093/annweh/wxx037.

(4) Dang, H.; Liu, D.; Hou, X.; Wu, Y.; Wang, B.; Dong, H.; Xian, Y. One-Step Extraction Prior to Two-Step Detection by Ultra High Performance Liquid Chromatography Tandem Mass Spectrometry (UHPLC-MS/MS) to Determine Different Groups of Antibacterial Agents in Infant Disposable Hygiene Products. *Anal. Methods* **2017**, *9* (3), 482–489. https://doi.org/10.1039/c6ay03220f.

(5) Heyde, B. J.; Barthel, A.; Siemens, J.; Mulder, I. A Fast and Robust Method for the Extraction and Analysis of Quaternary Alkyl Ammonium Compounds from Soil and Sewage Sludge. *PLoS One* **2020**, *15* (8 August), 1–16. https://doi.org/10.1371/journal.pone.0237020.

(6) Koeber, R.; Pappas, A.; Michaelis, U.; Schulze, B. Extraction and Quantification of the Cationic Lipid 1,2-Dioleoyl-3-Trimethylammonium Propane from Human Plasma. *Anal. Biochem.* **2007**, *363* (1), 157–159. https://doi.org/10.1016/j.ab.2007.01.001.

(7) McAnoy, A. M.; Wu, C. C.; Murphy, R. C. Direct Qualitative Analysis of Triacylglycerols by Electrospray Mass Spectrometry Using a Linear Ion Trap. *J. Am. Soc. Mass Spectrom.* **2005**, *16* (9), 1498–1509. https://doi.org/10.1016/j.jasms.2005.04.017.

(8) Molloy, B. J. LipidQuan-R for Diacyl-Phosphatidylcholines in Human Serum: A Rapid, Targeted UPLC-MS/MS Method for Lipidomic Research Studies. **2018**, 1–6.

(9) Bhaskar, V. V.; Middha, A.; Srivastava, P.; Rajagopal, S. Liquid Chromatography/Tandem Mass Spectrometry Method for Quantitative Estimation of Solutol HS15 and Its Applications. *J. Pharm. Anal.* **2015**, *5* (2), 120–129. https://doi.org/10.1016/j.jpha.2014.09.002.

(10) Hvattum, E.; Yip, W. L.; Grace, D.; Dyrstad, K. Characterization of Polysorbate 80 with Liquid Chromatography Mass Spectrometry and Nuclear Magnetic Resonance Spectroscopy: Specific Determination of Oxidation Products of Thermally Oxidized Polysorbate 80. *J. Pharm. Biomed. Anal.* **2012**, *62*, 7–16. https://doi.org/10.1016/j.jpba.2011.12.009.

(11) Kühn, A. V.; Raith, K.; Sauerland, V.; Neubert, R. H. H. Quantification of Hyaluronic Acid Fragments in Pharmaceutical Formulations Using LC-ESI-MS. *J. Pharm. Biomed. Anal.* **2003**, *30* (5), 1531–1537. https://doi.org/10.1016/S0731-7085(02)00544-7.

(12) Vigliano, M.; Bianchera, A.; Bettini, R.; Elviri, L. Determination of Hyaluronic Acid in a Chitosan-Based Formulation by RP C18 and HILIC LC-ESI-MS: An Evaluation of Matrix Effect. *Chromatographia* **2013**, *76* (23–24), 1761–1766. https://doi.org/10.1007/s10337-013-2533-4.

(13) Stern, R.; Kogan, G.; Jedrzejas, M. J.; Šoltés, L. The Many Ways to Cleave Hyaluronan. *Biotechnol. Adv.* **2007**, *25* (6), 537–557. https://doi.org/10.1016/j.biotechadv.2007.07.001.

(14) Toghi Eshghi, S.; Yang, W.; Hu, Y.; Shah, P.; Sun, S.; Li, X.; Zhang, H. Classification of Tandem Mass Spectra for Identification of N- and O-Linked Glycopeptides. *Sci. Rep.* **2016**, *6* (November), 1–8. https://doi.org/10.1038/srep37189.

(15) Jiang, H.; Desaire, H.; Butnev, V. Y.; Bousfield, G. R. Glycoprotein Profiling by Electrospray Mass Spectrometry. *J. Am. Soc. Mass Spectrom.* **2004**, *15* (5), 750–758. https://doi.org/10.1016/j.jasms.2004.01.009.

(16) Ma, X.; Dagan, S.; Somogyi, Á.; Wysocki, V. H.; Scaraffia, P. Y. Low Mass MS/MS Fragments of Protonated Amino Acids Used for Distinction of Their 13C- Isotopomers in Metabolic Studies. *J. Am. Soc. Mass Spectrom.* **2013**, *24* (4), 622–631. https://doi.org/10.1007/s13361-012-0574-9.
